# Systematics of the *Dendropsophus leucophyllatus* species complex (Anura: Hylidae): Cryptic diversity and the description of two new species

**DOI:** 10.1371/journal.pone.0171785

**Published:** 2017-03-01

**Authors:** Marcel A. Caminer, Borja Milá, Martin Jansen, Antoine Fouquet, Pablo J. Venegas, Germán Chávez, Stephen C. Lougheed, Santiago R. Ron

**Affiliations:** 1 Museo de Zoología, Escuela de Biología, Pontificia Universidad Católica del Ecuador, Quito, Ecuador; 2 National Museum of Natural Sciences, Spanish Research Council (CSIC), Madrid, Spain; 3 Senckenberg Gesellschaft für Naturforschung, Frankfurt am Main, Germany; 4 CNRS Guyane USR LEEISA, Centre de recherche de Montabo, Cayenne, French Guiana; 5 División de Herpetología-Centro de Ornitología y Biodiversidad (CORBIDI), Urb. Huertos de San Antonio, Surco, Lima-Perú; 6 Department of Biology, Queen’s University, Kingston, Ontario, Canada; Universitat Trier, GERMANY

## Abstract

Genetic data in studies of systematics of Amazonian amphibians frequently reveal that purportedly widespread single species in reality comprise species complexes. This means that real species richness may be significantly higher than current estimates. Here we combine genetic, morphological, and bioacoustic data to assess the phylogenetic relationships and species boundaries of two Amazonian species of the *Dendropsophus leucophyllatus* species group: *D*. *leucophyllatus* and *D*. *triangulum*. Our results uncovered the existence of five confirmed and four unconfirmed candidate species. Among the confirmed candidate species, three have available names: *Dendropsophus leucophyllatus*, *Dendropsophus triangulum*, and *Dendropsophus reticulatus*, this last being removed from the synonymy of *D*. *triangulum*. A neotype of *D*. *leucophyllatus* is designated. We describe the remaining two confirmed candidate species, one from Bolivia and another from Peru. All confirmed candidate species are morphologically distinct and have much smaller geographic ranges than those previously reported for *D*. *leucophyllatus* and *D*. *triangulum* sensu lato. *Dendropsophus leucophyllatus* sensu stricto occurs in the Guianan region. *Dendropsophus reticulatus* comb. nov. corresponds to populations in the Amazon basin of Brazil, Ecuador, and Peru previously referred to as *D*. *triangulum*. *Dendropsophus triangulum* sensu stricto is the most widely distributed species; it occurs in Amazonian Ecuador, Peru and Brazil, reaching the state of Pará. We provide accounts for all described species including an assessment of their conservation status.

## Introduction

The destruction and alteration of natural areas is resulting in unprecedented rates of extinctions [1]. Because many species remain undescribed, efforts to catalog and quantify biodiversity elements must be prioritized [2]. This is particularly needed in widespread taxa with pervasive taxonomic problems. The genus *Dendropsophus* Fitzinger, 1843 represents a prime example of this as there are numerous species complexes [3–5]. *Dendropsophus* contains 99 formally described species [6] currently classified into nine species groups according to Faivovich et al. [7]: *Dendropsophus columbianus*, *D*. *garagoensis*, *D*. *labialis*, *D*. *marmoratus*, *D*. *microcephalus*, *D*. *minimus*, *D*. *minutus*, *D*. *parviceps*, and *D*. *leucophyllatus* species group.

The *Dendropsophus leucophyllatus* species group is currently comprised of 10 nominal species [6–8] distributed in eastern Central America and Chocó region: *Dendropsophus ebraccatus* (Cope, 1874); Amazonia: *D*. *bifurcus* (Andersson, 1945), *D*. *leucophyllatus* (Beireis, 1783), *D*. *manonegra* Rivera-Correa and Orrico, 2013, *D*. *rossalleni* (Goin, 1957), *D*. *salli* Jungfer et al., 2010, *D*. *sarayacuensis* (Shreve, 1935), and *D*. *triangulum* (Günther, 1869); and Atlantic forest: *D*. *anceps* (Lutz, 1929) and *D*. *elegans* (Wied-Neuwied, 1824). The first taxonomic review of this group was based on morphological characters [9], and subsequently refined with molecular analysis [3,7,8,10–18], which were sometimes combined with bioacoustic data [4,19].

In Amazonia, two currently recognized species are widespread, *Dendropsophus leucophyllatus* and *D*. *triangulum*. Both are characterized by dark dorsal markings on a light background (or *vice versa*), flash colors on the hidden surfaces of thighs, groins, and interdigital webbings, and a large axillary membrane [7,9,20,21]. Most recent accounts [9,20–24] indicate that *Dendropsophus leucophyllatus* has a distinctive hour-glass-shaped dark brown dorsal mark on a creamy tan background, and a different pattern consists of all dorsal surfaces of the body and limbs, as well as the flanks, being brown with a fine network of creamy white lines. *Dendropsophus triangulum* has color morphs varying from uniformly yellow to yellow with abundant dark brown circles. Chek et al. [11] and Lougheed et al. [19] showed that some populations of *Dendropsophus leucophyllatus* were more closely related to *D*. *triangulum* than to other populations of *D*. *leucophyllatus* based on molecular and bioacoustics evidence. More recently, Jansen et al. [4] suggested that some Bolivian populations of *D*. *leucophyllatus* form a distinct genealogical lineage (“*D*. *leucophyllatus* A”) based on molecular, bioacoustic, and morphological evidence.

Herein we gather new genetic, morphological, and bioacoustic data to assess the phylogenetic relationships and species boundaries among populations of the *D*. *leucophyllatus-triangulum* complex using samples spanning Ecuador, Peru, Bolivia, Brazil, Suriname, Guyana and French Guiana. The results demonstrate the existence of nine candidate species of which five are confirmed. We describe two of them including their advertisement calls and variation in external morphology.

## Methods

### Ethics statement

Voucher specimens and tissue samples were obtained following ethical and technical protocols [25]. Vouchers were euthanized with commercial roxicaine (anesthetic spray), fixed in 10% buffered formalin and then later preserved in 70% ethanol. Field permits were issued by the Ecuadorian Ministry of Environment (001–11 IC-FAU-DNB/MA; 002-2012-CA-FAU-MAE-DPO-PNY; 005-12-IC-FAU-DNB/MA; 005-2009-INVESTIGACIÓN-B-DPMS/MAE; 008–09 IC-FAU-DNB/MA; 010-2013-FAU-MAE-DPAO-PNY), Ministerio de Desarrollo Sostenible, La Paz (Bolivia), and Servicio Nacional de Sanidad Agropecuaria e Inocuidad Alimentaria (SENASAG), Santa Cruz (Bolivia). This study was evaluated and approved by the DGA (Dirección General Académica) of the Pontificia Universidad Católica del Ecuador in accordance with the guidelines for environmental and social impacts of research projects. The Dirección General Académica committee individually evaluates each project to determine its observance of its norms for ethical scientific research. Genetic data for Ecuadorian specimens were obtained under Genetic Resources Access Contract No MAE-DNB-CM-2015-0025 issued by Ministerio de Ambiente del Ecuador to Pontificia Universidad Católica del Ecuador.

### Protocol for species delimitation

We used the characters, terminology, and format of Duellman [26]. Sex was determined based on gonads or by the presence of vocal sac folds in males. Measurements were made using digital calipers (± 0.01 mm). Snout-vent length is abbreviated as SVL. Examined specimens (listed in the type-series and S1 Appendix) are housed at the collections of the División de Herpetología, Centro de Ornitología y Biodiversidad (CORBIDI), Lima (Peru); Senckenberg Forschungsinstitut und Naturmuseum (SMF), Frankfurt (Germany); Museo de Historia Natural Noel Kempff Mercado (NKM), Santa Cruz (Bolivia); Museo de Zoología at Universidad de São Paulo (MZUSP), São Paulo (Brazil); National Museum of Natural History (USNM), Washington D. C. (USA); Muséum National d'Histoire Naturelle (MNHN), Paris (French); Antoine Fouquet CNRS-Guyane collection, Cayenne (French Guiana); and Museo de Zoología at Pontificia Universidad Católica del Ecuador (QCAZ), Quito (Ecuador). We also examined the type material of *Hyla triangulum* (holotype BMNH 1947.2.23.88), *Hyla reticulata* (holotype MNCN 3474), and *Hyla membranacea* (syntypes NHRM 1961) deposited at the British Museum of Natural History, the National Museum of Natural Sciences in Madrid, and the Swedish Museum of Natural History in Stockholm, respectively.

In the Diagnosis sections, coloration refers to preserved specimens unless otherwise noted. One quantitative and ten qualitative morphological characters were evaluated: (1) snout-vent length measurement, (2) axillary membrane, (3) webbing on fingers, (4) webbing on feet [i. present, ii. absent], (5) palmar tubercle, (6) pectoral patches [i. present, ii. absent], (7) dorsal coloration [shape and numbers of marks on the dorsum], (8) rounded spots or bands on the dorsal surface of the limbs [one to three, i. present, ii. absent], (9) reticulated dorsal pattern (which consists of a fine network of white or yellow lines on the dorsal region, sides of the head and body with a dark brown to brown background) [i. present, ii. absent], (10) ventral coloration, and (11) iris coloration. Coloration in life was obtained from digital color photographs.

Calls recorded by us were obtained with a Sennheiser K6–ME67 directional microphone and an Olympus LS-10 digital recorder. Calls described by Duellman and Pyles [27], Lougheed et al. [19], and Jansen et al. [4] were also included and reanalyzed. Spectrograms were generated with Raven 1.5 software [28] using a Fast Fourier Transformation (FFT) from a sample of 1024 points and a frequency resolution of 43.1 Hz. Calls of the members of the *D*. *leucophyllatus-triangulum* complex consist of two components, the first note (Type I) is a pulsed trill and the second note (Type II) is similar to Type I but shorter [19]. Call categorization (call types) follows Toledo et al. [29]. Call variables measured are defined in Table 1. If available, several calls or notes were analyzed per individual to calculate an individual average. Temporal variables were measured on the oscillogram, spectral variables on the power spectrum. Five call variables from the Type I note of the advertisement calls were used to run a Principal Components Analysis (PCA) in the program JMP^®^ 9.01 [30] to assess acoustic differentiation between eleven males of *D*. *triangulum* from Obidos, Santa Cecilia, Tambococha, and Chiroisla in Ecuador and thirty-one males of *Dendropsophus leucophyllatus* from Puerto Almacén, Beni, Buenavista, Los Lagos, and San Sebastián in Bolivia; Kaw mountain and Toponowini in French Guiana; Lorocachi, Santa Cecilia, Misahuallí and Estación Científica Yasuní PUCE in Ecuador; Río Branco, Alter do Chão, Redenção and Serra do Navio in Brazil; and Puerto Maldonado in Peru. Temperature can influence quantitative parameters of frog calls including dominant frequency, pulse rate, and call length [31]. However, variation in recording temperature was relatively low (< 3 C°) with the exception of clade A (range variation of 6.8 C°). Thus, temperature should not significantly influence our comparisons. Original recordings (see S1 Table) are deposited in the audio archive of the QCAZ (available at the AmphibiaWebEcuador website, http://zoologia.puce.edu.ec/Vertebrados/anfibios/), the Fonoteca Zoológica of MNCN (http://www.fonozoo.com/) and the Macaulay Library at the Cornell Lab of Ornithology (http://macaulaylibrary.org/).

**Table 1:** Call parameters of *Dendropsophus* spp. analyzed in this study.

Character	Description
Call duration	Time from the beginning of the first note to the end of the last note of the call
Number of notes per call	Number of notes in the call
Rise time of the call	Time from the beginning of the call to the point of its maximum amplitude
Number of pulses per call	Number of pulses in the call
Type I note duration	Time from the beginning to the end of the long note
Number of pulses of the Type I note	Number of pulses of the long note
Rise time of the Type I note	Time from the beginning of Type I note to the point of its maximum amplitude
Type II note duration	Time from the beginning to the end of the short note
Number of pulses of the Type II note	Number of pulses of the short note
Distance between notes	Time between the end of one note and the beginning of the following note
Average of the dominant frequency call	Average frequency of the harmonic with the maximum energy at the beginning, middle and end of the call
Frequency bandwidth	The higher frequency at any point of the call minus the lowest frequency at any point of the call
Distance between pulses	Time from the highest amplitude peak of a pulse to the highest amplitude peak of the following pulse

See text for details.

We also assessed the Red List status of each species according to the IUCN Red List criteria (IUCN 2001). Geographic ranges were estimated using minimum convex polygons in software ArcMap 10 [32]. We estimated the proportion of remaining natural vegetation within the distribution ranges with the layer of Amazon Deforestation 2010 (available at http://www.arcgis.com/home/item.html?id=8e10fae75bbf4a9fad1d190113f9d976). Vegetation types were based on WWF Ecoregions (available at http://wwf.panda.org/about_our_earth/ecoregions/ecoregion_list/) except for Ecuadorian localities, where we used the more detailed classification of Sierra et al. [33].

### DNA extraction, amplification, and sequencing

DNA sequences used in phylogenetic analyses were obtained as follows. Total DNA was extracted from muscle or liver tissue preserved in 95% ethanol or tissue storage buffer using either standard phenol-chloroform extraction protocols [34] or the Qiagen DNeasy extraction kit (QiagenTM, Valencia, CA) according to the manufacturer’s protocol. Polymerase chain reaction (PCR) was used to amplify the mitochondrial genes 12S rRNA, 16S rRNA, NADH dehydrogenase I (ND1) and cytochrome *c* oxidase I (CO1), as well as fragments of nuclear genes proopiomelanocortin (POMC), the recombination-activating gene 1 (RAG-1) and brain-derived neurotrophic factor (BDNF). PCR amplification was carried out using standard protocols, and primers are presented in S2 Table. Amplified products were sequenced by commercial laboratories (Macrogen Inc., Seoul, Korea and Secugen, Madrid, Spain).

### Phylogenetic analyses

To determine phylogenetic relationships between species of the *Dendropsophus leucophyllatus* species group we used the DNA sequences including sequences from GenBank. For the outgroup, we added samples of *Dendropsophus brevifrons*, *D*. *carnifex*, *D*. *parviceps* and *D*. *rhodopeplus* (based on Pyron and Wiens [16]). All samples used are listed in S3 Table. Sequence alignment was done with Geneious 5.4.4 software (GeneMatters Corp.) using the Geneious alignment algorithm. The matrix was imported into Mesquite (version 3.04; Maddison and Maddison [35]) and the alignment was adjusted by hand. Because it is likely that variation in each of our sampled genes (or codon positions within protein coding genes) were shaped by different evolutionary processes, we partitioned the data according to gene and codon position to analyze each partition under separate models of evolution. We used software PartitionFinder v. 1.1.1 [36] to simultaneously estimate both the best-fit model for each partition and the best partition strategy for our data.

Phylogenetic relationships were inferred separately for mitochondrial and nuclear genes using maximum-likelihood and Bayesian inference. Each Bayesian analysis consisted of eight parallel runs of the Metropolis coupled Monte Carlo Markov chain for 40 × 10^6^ generations. Each run had four chains with a temperature of 0.1. For each analysis, the chain was sampled every 1000 generations. The first 10% of sampled trees were discarded as burn-in and the remaining trees were used to estimate the Bayesian tree, posterior probabilities, and other model parameters. The prior for the rate matrix was a uniform Dirichlet distribution and all topologies were equally probable a priori. We considered to have achieved convergence to stationarity when the average standard deviation split frequencies were < 0.05 between runs. Tracer software version 1.5 [37] was used to confirm convergence and stationarity of the parameter estimates using an ESS threshold of 200. Phylogenetic analyses were carried out in MrBayes 3.2 [38]. Maximum likelihood analyses were conducted using GARLI 2.0 [39] with default settings. Analyses were terminated after 10 000 generations without an improvement in tree topology. We ran a total of 20 independent searches and used random starting addition (streefname = random) to reduce the probability of inferring a suboptimal likelihood solution. Support was evaluated using 100 bootstrap replicates with each replicate terminated after 5000 replications without improvement in topology. Uncorrected mean between-species genetic distances were calculated in MEGA 5 [40] for gene 16S given that this is generally used to compare divergence among Amphibian species (e.g. [3,4,41]).

### Species delineation

Species limits among populations of *Dendropsophus leucophyllatus*-*triangulum* were evaluated using three independent character sets: genetic, bioacoustic, and morphological. Following Fouquet et al. [3], we classified pairwise genetic uncorrected *p*-distances in the 16S rRNA gene as follows: low divergence, below 3%; moderate divergence, 3–5%; high divergence, above 5%; very high divergence, above 7%. We used the predefined 3% threshold for species delimitation (i.e., groups with distances > 3.0% were considered candidate species [3]). This is a conservative threshold because various studies have shown that sister species among hylids are frequently separated by lower distances (e.g. [8,41–43]). We classified genetic lineages into the following categories: (1) Confirmed Candidate Species (CCS) when there was covariation between genetic and bioacoustic or morphological data sets, (2) Deep Conspecific Lineages (DCL) when such covariation was not found, and (3) Unconfirmed Candidate Species (UCS) when bioacoustic and morphological data were unavailable. This approach was adapted from Vieites et al. [41].

### Nomenclatural acts

The electronic edition of this article conforms to the requirements of the amended International Code of Zoological Nomenclature [44], and hence the new names contained herein are available under that Code from the electronic edition of this article. This published work and the nomenclatural acts it contains have been registered in ZooBank, the online registration system for the ICZN. The ZooBank LSIDs (Life Science Identifiers) can be resolved and the associated information viewed through any standard web browser by appending the LSID to the prefix “http://zoobank.org/”. The LSID for this publication is: urn:lsid:zoobank.org:pub:6DF93C61-5696-4D54-AA5D-F88B0F3AA883. The electronic edition of this work was published in a journal with an ISSN, and has been archived and is available from the following digital repositories: PubMed Central, LOCKSS.

## Results

### Phylogenetic relationships

**Fig 1:**
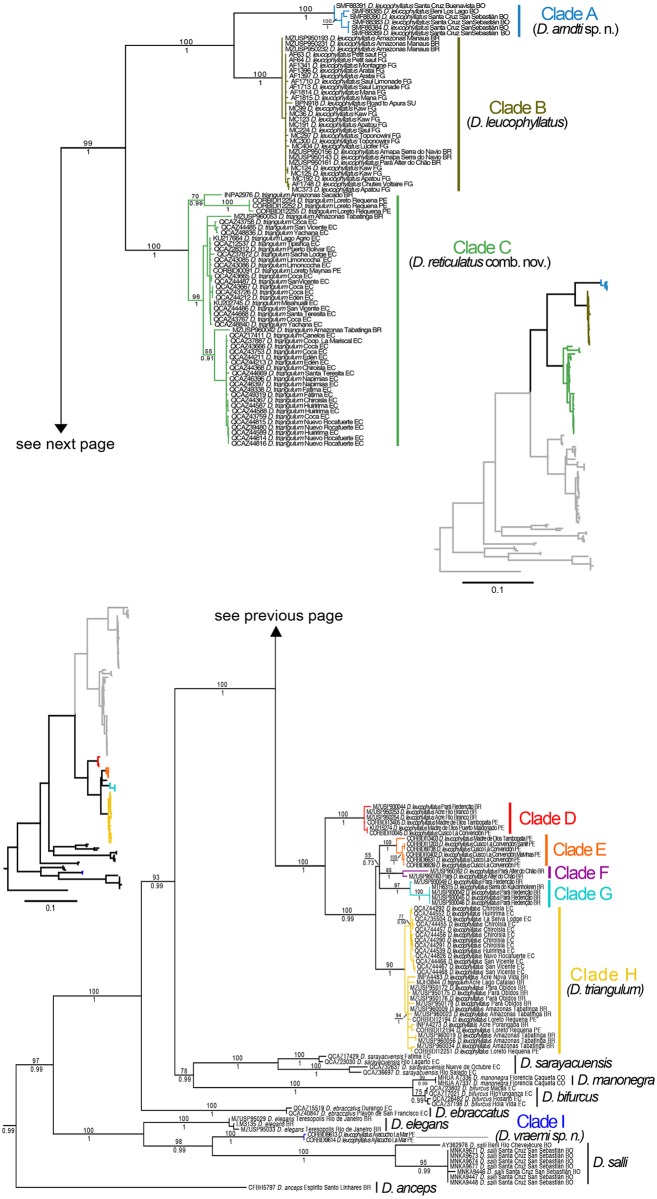
Maximum likelihood phylogram depicting relationships within the *Dendropsophus leucophyllatus* species group. The phylogram was derived from analysis of 3400 bp of mitochondrial DNA (gene fragments *12S*, *16S*, *ND1* and *CO1*). Museum catalog numbers and localities are shown for each sample. Bootstrap values are shown above the branches and Bayesian posterior probabilities are shown below; missing values indicate values below 50 (bootstrap) or 0.5 (posterior probability). Outgroup species (*Dendropsophus brevifrons*, *D*. *carnifex*, *D*. *parviceps* and *D*. *rhodopeplus*) are not shown. Abbreviations are: **BO** Bolivia, **CO** Colombia, **EC** Ecuador, **FG** French Guiana, **PE** Peru, **SU** Suriname.

The complete DNA sequence data matrix contained seven genes and 5,701 bp for 168 samples. For the analyses based on the mitochondrial genes only, PartitionFinder choose five partitions as the best strategy (best model in parentheses): 12S, 16S and ND1 1 ^st^ position (GTR + I + G), CO1 3 ^rd^ and ND1 3 ^rd^ position (GTR + I + G), CO1 1 ^st^ position (SYM + I + G), CO1 2 ^nd^ position (F81), ND1 2 ^nd^ position (HKY + I + G). For the analyses based on nuclear analysis, two partitions were chosen: RAG1, POMC and BDNF 1 ^st^ and 2 ^nd^ position for all genes (HKY), and 3 ^rd^ position for the same three genes (K80+G). The tree topologies for the Maximum likelihood and Bayesian phylogenies were similar except for weakly supported nodes (posterior probability, pp < 0.73 and bootstrap < 55). The phylogenetic relationships recovered from the analysis of the mitochondrial DNA sequences (Fig 1) were consistent with those reported by Rivera-Correa and Orrico [8] for the *D*. *leucophyllatus* group.

Specimens assigned to *Dendropsophus leucophyllatus* and *D*. *triangulum* grouped into nine clades (A to I; Fig 1). Each clade is well supported (pp > 0.98; bootstrap > 75). Samples of *D*. *leucophyllatus* from Ayacucho, Peru (Clade I) are more closely-related to a clade formed by *D*. *salli* and *D*. *elegans* than to other populations of *D*. *leucophyllatus*. All the other populations of *D*. *leucophyllatus* and *D*. *triangulum* form a large clade with high support, in which *D*. *triangulum* is nested within *D*. *leucophyllatus* (Fig 1). This major Amazonian clade comprises eight subclades divided in two major groups (A, B, C and D, E, F, G, H in Fig 1).

**Fig 2:**
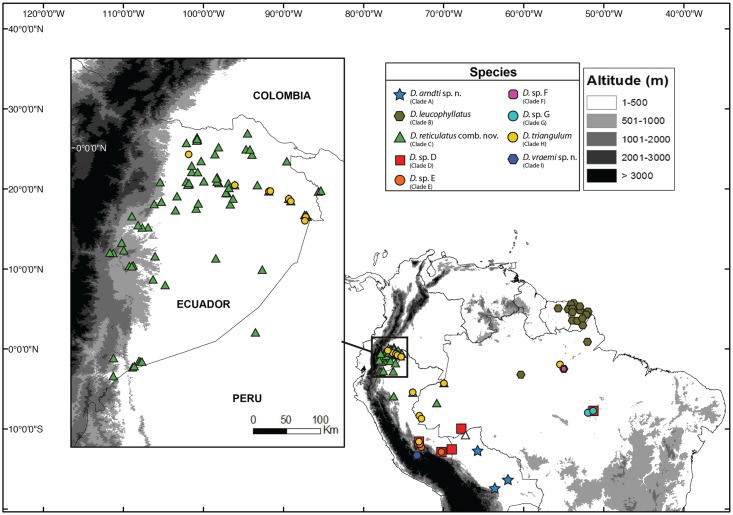
Distribution of species of the *Dendropsophus leucophyllatus* complex. Localities are based of specimens deposited at Museo de Zoología of Pontificia Universidad Católica del Ecuador, Museo de Zoología of Universidad de São Paulo, the Herpetology collection of Senckenberg Forschungsinstitut und Naturmuseum and Centro de Ornitología y Biodiversidad CORBIDI. Locality data from the literature Moravec and Aparicio (2004) is shown in a hollow triangle.

In the first major group, clades A and B are sister to clade C. Clade A includes specimens from Bolivia (departments of Beni and Santa Cruz); the sister clade B is distributed in northeastern Brazil (states of Amazonas and Pará north of the Amazon River and the state of Amapá), Suriname and French Guiana; clade C comprises specimens from Ecuador traditionally assigned to *D*. *triangulum* (provinces of Sucumbíos, Napo, Orellana, Pastaza and Morona Santiago), northwestern Brazil (State of Amazonas) and northeastern Peru (Region of Loreto) (Fig 2).

In the second major group (D–H in Fig 1), there is a well-supported (pp = 1; bootstrap = 100) basal divergence between clade D and the remaining clades. Clade D is distributed in Brazil (states of Acre and Pará) and southeastern Peru (regions of Cusco and Madre de Dios); clade E occurs in southeastern Peru (regions of Cusco and Madre de Dios); clades F and G are distributed in northeastern Brazil (State of Pará); clade H occurs in Ecuador (provinces of Orellana and Sucumbíos), northwestern, northeastern Brazil (states of Acre, Pará and Amazonas), north, and southeastern Peru (regions of Loreto and Cusco) (Fig 2).

**Fig 3:**
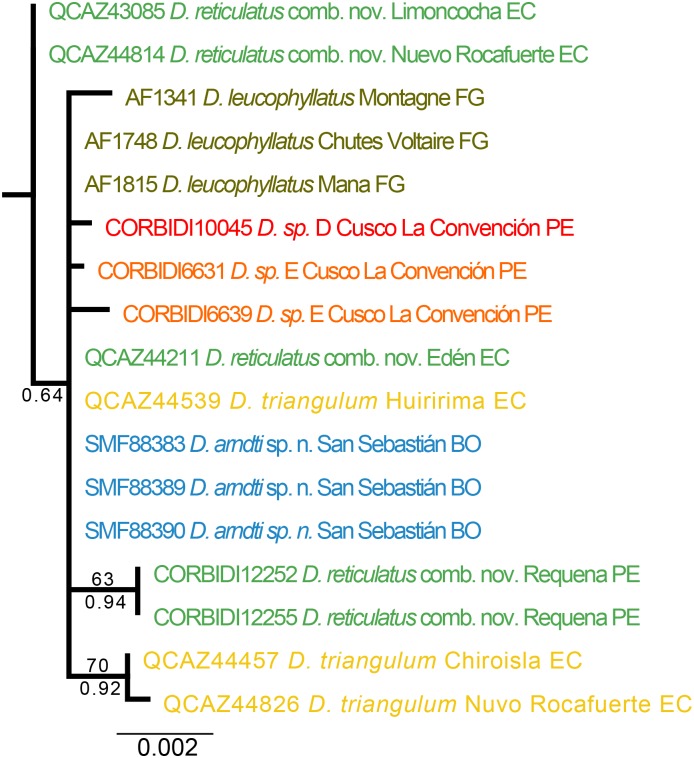
Maximum likelihood phylogram depicting relationships within the *Dendropsophus leucophyllatus-triangulum* complex. The phylogram was derived from analysis of 1605 bp of nuclear DNA (gene fragments *RAG-1*, *BDNF* and *POMC*). Museum catalog numbers and localities are shown for each sample. Bootstrap values are shown above the branches and Bayesian posterior probabilities are shown below; missing values indicate values below 50 (bootstrap) or 0.5 (posterior probability). Colors and binomen names refer to clades identified using mtDNA (see Fig 1).

The Maximum Likelihood and Bayesian consensus trees, derived from the RAG1, POMC, and BDNF nuclear genes have low resolution and weak support for most nodes (Fig 3).

### Species delimitation

Our analyses revealed that the binomen *Dendropsophus leucophyllatus* and *D*. *triangulum* actually comprise nine candidate species. Sequence divergence (uncorrected *p*-distance for 16S) ranges from 2.5 to 15.8% among clades and from 0 to 1.1% within clades (Table 2). Although the genetic distance between clades F and H is below the 3% threshold for species delimitation (2.5%), this appears to be an artifact of the short length of the 16S sequence for clade F (506 bp). We treat clade F as a tentative candidate species based on the branch lengths that separate it from its closest relatives in the phylogeny, clades E and G (Fig 1).

**Table 2:** Pairwise genetic distances (uncorrected p) of 16S DNA sequences among members of the *Dendropsophus leucophyllatus-triangulum* complex.

	*D*. *arndti* sp. n. (Clade A)	*D*. *leucophyllatus* (Clade B)	*D*. *reticulatus* comb. nov. (Clade C)	*D*. sp. D	*D*. sp. E	*D*. sp. F	*D*. sp. G	*D*. *triangulum* (Clade H)	*D*. *vraemi* sp. n.
***D*. *arndti* sp. n. (Clade A)**	0.002 ± 0.001 (0–0.005)	35	52	10	12	8	11	34	8
***D*. *leucophyllatus* (Clade B)**	0.057 ± 0.001 (0.052–0.058)	0 ± 0.0007 (0–0.002)	75	33	35	31	34	57	31
***D*. *reticulatus* comb. nov. (Clade C)**	0.096 ± 0.003 (0.084–0.1)	0.067 ± 0.002 (0.059–0.075)	0.007 ± 0.006 (0–0.026)	50	52	48	51	74	48
***D*. sp. D (Clade D)**	0.106 ± 0.001 (0.101–0.104)	0.106 ± 0.048 (0.101–0.108)	0.096 ± 0.002 (0.091–0.101)	0.003 ± 0.001 (0–0.005)	10	6	9	32	6
***D*. sp. E (Clade E)**	0.128 ± 0.002 (0.121–0.131)	0.132 ± 0.0006 (0.127–0.131)	0.108 ± 0.044 (0.098–0.117)	0.068 ± 0.002 (0.065–0.071)	0.004 ± 0.003 (0–0.008)	8	11	34	8
***D*. sp. F (Clade F)**	0.094 ± 0.001 (0.088–0.094)	0.095 ± 0.00007 (0.0912–0.0916)	0.075 ± 0.005 (0.065–0.084)	0.064 ± 0.003 (0.059–0.068)	0.057 ± 0.006 (0.049–0.065)	0.011	7	30	4
***D*. sp. G (Clade G)**	0.109 ± 0.004 (0.094–0.111)	0.109 ± 0.003 (0.097–0.107)	0.094 ± 0.007 (0.075–0.103)	0.061 ± 0.005 (0.049–0.065)	0.051 ± 0.003 (0.043–0.055)	0.038 ± 0.007 (0.02–0.044)	0.007 ± 0.008 (0–0.017)	33	7
***D*. *triangulum* (Clade H)**	0.096 ± 0.003 (0.085–0.101)	0.098 ± 0.002 (0.091–0.107)	0.08 ± 0.005 (0.081–0.091)	0.054 ± 0.005 (0.046–0.065)	0.056 ± 0.006 (0.046–0.068)	0.025 ± 0.005 (0.02–0.037)	0.037 ± 0.007 (0.017–0.047)	0.007 ± 0.004 (0.–0.017)	30
***D*. *vraemi* sp. n. (Clade I)**	0.12 ± 0.001 (0.119–0.122)	0.135 ± 0.0006 (0.131–0.135)	0.115 ± 0.003 (0.107–0.124)	0.138 ± 0.001 (0.133–0.136)	0.136	0.142 ± 0.0003	0.158 ± 0.006 (0.142–0.157)	0.133 ± 0.004 (0.124–0.138)	2

Mean ± SD is given with range in parentheses (below diagonal). Number of individuals compared is shown above diagonal. Diagonal shows intra-clade genetic distances.

Table 3 summarizes comparisons of males’ SVL. There are significant differences in SVL between most clades pairs. Members of clades C and I are smaller than those of A, B and H (all *P* values for *t* tests < 0.001); members of clade I are larger than those of C (*t* = 6.52, df = 9, *P* < 0.001); and members of clade H are larger than those of A (*t* = 2.71, df = 25, *P* = 0.01) and B (*t* = 4.18, df = 19, *P* < 0.001).

**Table 3:** Snout-vent length of *Dendropsophus arndti* sp. n., *D*. *leucophyllatus*, *D*. *reticulatus* comb. nov., *D*. sp. D, *D*. sp. E, *D*. sp. F, *D*. sp. G, *D*. *triangulum* and *D*. *vraemi* sp. n.

Species	SVL males	SVL females
*D*. *arndti* sp. n. (Clade A)	30 ± 1.5 (28–32.4); n = 13	33.2; n = 1
*D*. *leucophyllatus* (Clade B)	29.7 ± 1.7 (26.8–32.3); n = 12	37.9 ± 2.7 (36–39.8); n = 2
*D*. *reticulatus* comb. nov. (Clade C)	23.8 ± 1.5 (20–29.6); n = 185	33.1 ± 2.9 (28–39.7); n = 66
*D*. sp. D (Clade D)	30.7 ± 0.9 (29.8–31.5); n = 3	29.3 ± 1.7 (26.8–32.3); n = 12
*D*. sp. E (Clade E)	27.3 ± 2.1 (25.2–29.4); n = 3	36.9; n = 1
*D*. sp. F (Clade F)	29.9 ± 0.3 (29.7–30.2); n = 2	—
*D*. sp. G (Clade G)	30.5 ± 0.9 (29.5–31.6); n = 5	—
*D*. *triangulum* (Clade H)	31.7 ± 1.5 (28.6–34.4); n = 26	40 ± 1.76 (37.5–41.9); n = 5
D. *vraemi* sp. n. (Clade I)	26 ± 0.9 (25.1–27.6); n = 8	—

Mean ± SD is given with range in parentheses. The *n* values indicate the number of individuals analyzed.

**Fig 4:**
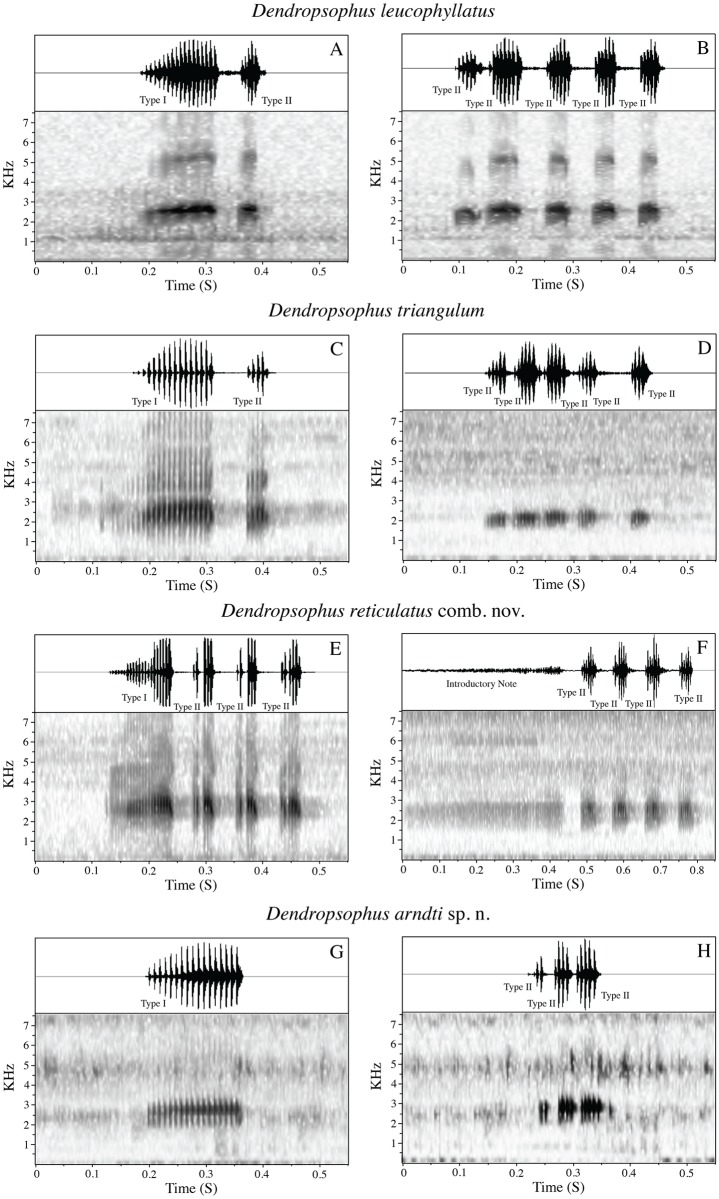
Sound spectrogram and corresponding oscillogram (above) of the calls of *Dendropsophus leucophyllatus* complex. **A–B**: *D*. *leucophyllatus* (MZUSP 950161) from Alter do Chão, State of Pará; **C–D**: *D*. *triangulum* (QCAZA 44290) from Chiroisla, Province Orellana; **E–F**: *D*. *reticulatus* comb. nov. (QCAZA 369 and 49174) from Misahuallí and Maxus, Napo and Orellana provinces; **G–H**: *D*. *arndti* sp. n. (SMF 88390) from Yucuma, Province Beni. **A**, **C**, **E**, **G** are advertisement call and **B**, **D**, **F**, **H** aggressive calls.

We found two types of calls in the *D*. *leucophyllatus-triangulum* complex. Both types were composed of one or more pulsed notes, but with differences in their physical structure, and the social contexts where they were emitted. The first type appears to be advertisement calls. Males in choruses produced these calls repeatedly and antiphonally. The call consists of one pulsed trill note (Type I) sometimes followed by other secondary notes (Type II) with shorter duration and fewer pulses (Fig 4A, 4C, 4E and 4G). The second type appears to correspond to aggressive calls. They were emitted at high rates in dense choruses. The number of notes for this call varies from two to seven (Type II; Fig 4B, 4D, 4F and 4H). Further studies are necessary to corroborate these classifications.

**Fig 5:**
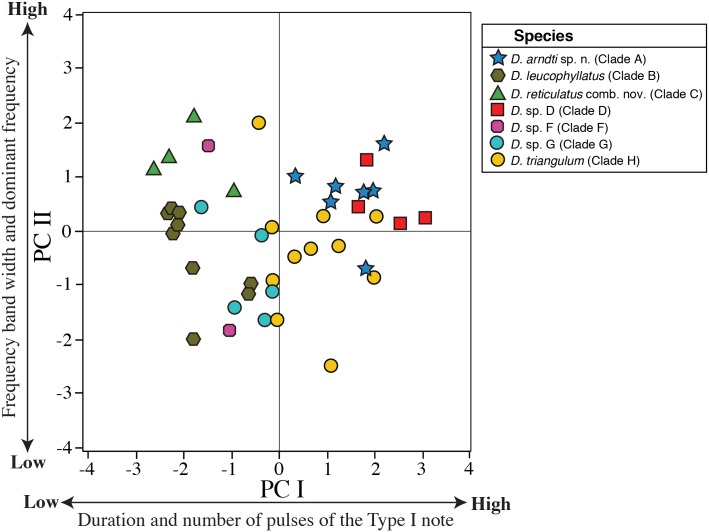
Axes I and II from Principal Components Analysis based on five acoustic variables from the advertisement calls of males of the *Dendropsophus leucophyllatus-triangulum* complex. *Dendropsophus arndti* sp. n. (7 males), *D*. *leucophyllatus* (9), *D*. *triangulum* (11), *D*. *reticulatus* comb. nov. (4), *D*. sp. D (4), *D*. sp. F (2), and *D*. sp. G (5). See Table 4 for character loadings on each component.

Our PCA of advertisement calls from 42 males resulted in two PCs with eigenvalues > 1.0. These two PCs accounted for 76.44% of the total variation. PC I (51.82% of the variance) had high loadings on number of pulses and duration of the Type I note; PC II (26.62% of the variance) had high loadings on frequency bandwidth and dominant frequency (Table 4). The acoustic space (as represented by PC I and PC II; Fig 5) showed significant differences between clades B, C, F and G relative to A, D and H (all *P* values for *t* tests < 0.001). PC II scores were significantly different between clade B and C (*t* = 4.33, df = 8, *P* = 0.002), A (*t* = -4.01, df = 12, *P* = 0.001) and D (*t* = 2.46, df = 9, *P* = 0.03); while clade A and C differed significantly from H and G (all *P* values for *t* tests < 0.02).

**Table 4:** Character loadings, eigenvalues, and percentage of explained variance for Principal Components (PC) I–II of specimens of the *D*. *leucophyllatus-triangulum* complex.

	Character Loading	
Variable	PC I	PC II
Type I note duration	**0.56**	0.25
Number of pulses of the Type I note	**0.53**	0.31
Rise time of the Type I note	0.43	0.2
Frequency bandwidth	–0.28	**0.68**
Dominant frequency	–0.35	**0.57**
Eigenvalue	2.59	1.23
%	51.82	24.62

The analysis was based on five acoustic variables from the advertisement calls of *Dendropsophus arndti* sp. n., *D*. *leucophyllatus*, *D*. *triangulum*, *D*. *reticulatus* comb. nov., *D*. sp. D, *D*. sp. F, and *D*. sp. G. Bold figures indicate the highest loadings.

Clades A–C and H have unique morphological and bioacoustic features (see species diagnosis sections), suggesting that each clade represents a confirmed candidate species (CCS). In contrast, lack of complete morphological and bioacoustic data for clades D, E, F, and G preclude us from determining whether they are conspecific to clade H (*D*. *triangulum* sensu stricto) or represent up to four valid species. Clades D–G have a color pattern similar to clade H suggesting that they are conspecific (see S1 Fig). However, sample sizes for clades D and E are insufficient to draw conclusions. Therefore, under the available evidence, we consider clades D–G unconfirmed candidate species (UCS).

Samples from Ayacucho (clade I) are sister to *D*. *salli* from Bolivia. Morphologic and genetic differences with *D*. *salli* (uncorrected *p*-distance range 10.1–10.4% in gen 16S) show that clade I represents a Confirmed Candidate Species.

We also found deep intraspecific divergences within populations of *D*. *sarayacuensis* in Ecuador and *D*. *salli* in Bolivia. For *D*. *sarayacuensis*, uncorrected *p*-distances average between the populations of Fátima and Río Largo (provinces of Pastaza and Tungurahua) vs. Nueve de Octubre and Río Salado (provinces of Morona Santiago and Napo) is 6%; For *D*. *salli*, *p*-distances average between Río Chevejécure (Department of Beni) and San Sebastián (Department of Santa Cruz) is 3% (Fig 1). These divergent lineages are left as UCS until additional information is available to assess their status.

### Taxonomic review

The available names for the candidate species sampled in our phylogenies are *Dendropsophus leucophyllatus* (Beireis, 1783), *Hyla frontalis* Daudin, 1800, *Dendropsophus triangulum* (Günther, 1869), *Hyla reticulata* Jiménez de la Espada, 1870, *Hyla favosa* Cope, 1885, *Hyla membranacea* Andersson, 1945, *Hyla laynei* Goin, 1957, and *Hyla oliveae* Cochran and Goin, 1970. Examination of two of the holotypes and published descriptions from the literature of the remaining types allowed us to assign the available names to clades B, C and H (Fig 1). We document those assignments in the following section.

#### Taxonomic status of *D*. *leucophyllatus*

**Fig 6:**
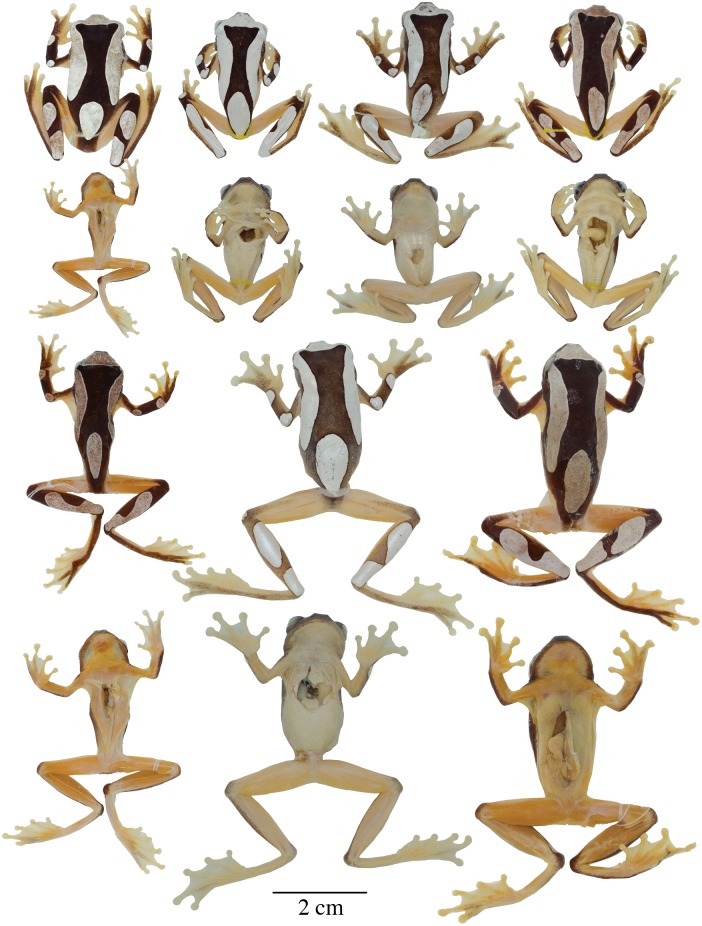
Adult preserved specimens of *Dendropsophus leucophyllatus* showing variation in dorsal and ventral coloration of preserved specimens. From left to right, first and second rows: MNHN2015.129 (neotype), MNHN2015.132, MNHN2015.134, MNHN2015.133 (males); third and fourth rows: MNHN2015.127 (male), MNHN2015.131, MNHN2015.128 (females). See S1 Appendix for locality data. All specimens are shown at the same scale.

The holotype of *Dendropsophus leucophyllatus* could not be examined because it is lost [6,45]. Nevertheless, the descriptions of the holotype of *D*. *leucophyllatus* by Beireis [46] and Böhme [45] indicate that *D*. *leucophyllatus* is conspecific to clade B (Fig 1). We base this conclusion on: (1) the external morphology of the holotype, which resembles that of clade B; the holotype was described as having a leaf-shaped blotch on the posterior end of the back, a character present in clade B. (Fig 6); (2) Type locality: The available data suggest that Clade B is the only lineage of the *D*. *leucophyllatus* species group occurring in Suriname (Fig 2), the country where the holotype was collected. Furthermore, specimens of *D*. *leucophyllatus* from the Guianas are characterized by having low morphological and genetic variation (Figs 1 and 6). With the purpose of clarifying the status of *D*. *leucophyllatus* for future taxonomic reviews, we designate a neotype from Sinnamary, French Guiana, a locality near Suriname.

The morphology of *Dendropsophus leucophyllatus* sensu stricto (Clade B) differs from Duellman’s [9] characterization who describes an hour-glass-shaped dark brown dorsal mark as distinctive of the species. The holotype of *D*. *leucophyllatus* and specimens from Clade B do not have that mark but have instead a leaf-shaped mark in the sacral region. Most specimens reported by Duellman [9] as “*D*. *leucophyllatus*” are from Amazonian Brazil (Belém), Colombia, Ecuador, and Peru and resemble individuals from clades D, E, F, G and H.

#### Taxonomic status of *D*. *triangulum* and *Hyla reticulata*

**Fig 7:**
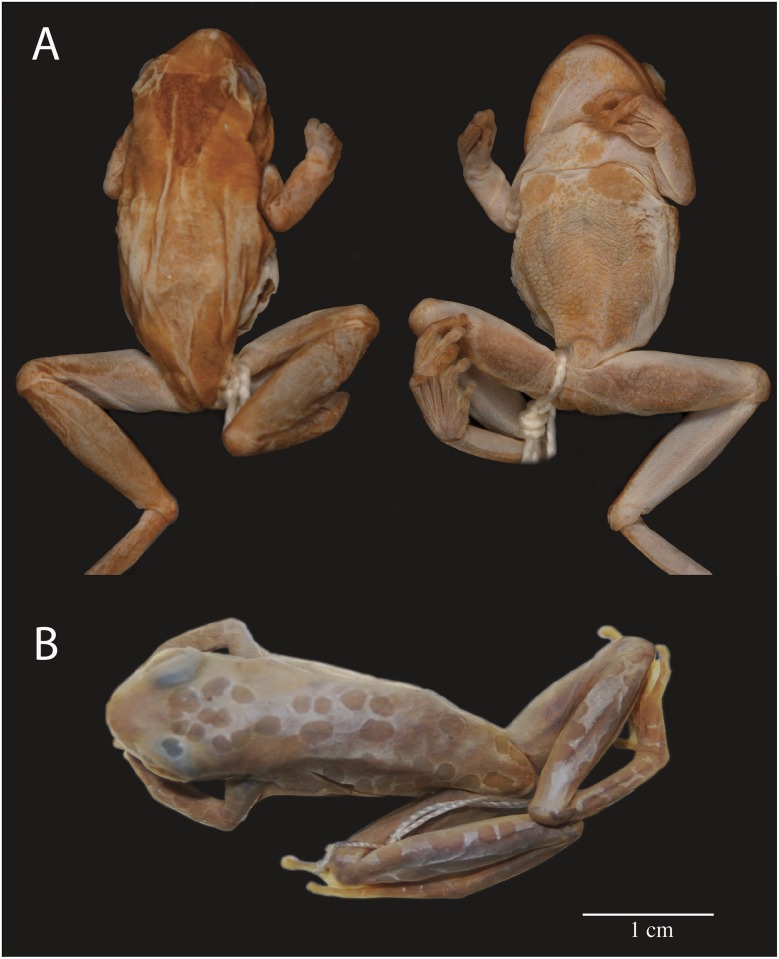
Holotypes examined. **A)** Dorsal and ventral views of the holotype of *Hyla triangulum* (holotype BMNH 68.11.15.2); **B)** dorsal view of the holotype of *Hyla reticulata* (holotype MNCN 3474).

**Fig 8:**
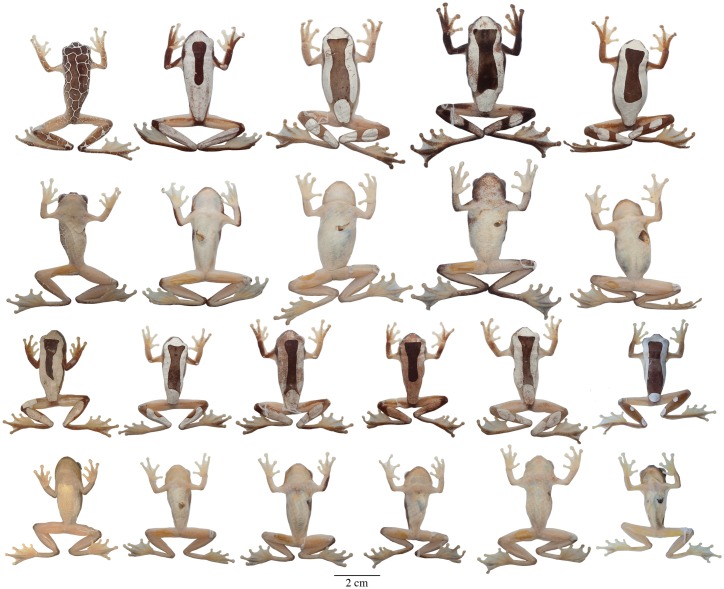
Adult preserved specimens of *Dendropsophus triangulum* showing variation in dorsal and ventral coloration. From left to right, first and second rows: QCAZA 44539 (male), 35504, 44293, 44458, 44552 (females); third and fourth rows: CORBIDI 12194, QCAZA 44290, 44466, 44467, 44471, CORBIDI 11204 (males). See S1 Appendix for locality data. All specimens are shown at the same scale.

**Fig 9:**
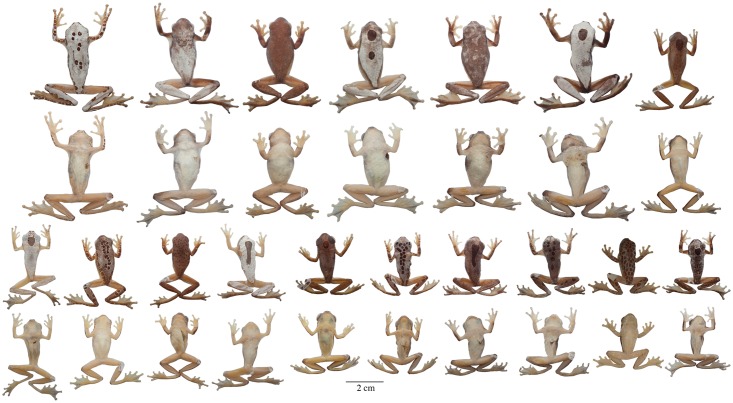
Adult preserved specimens of *Dendropsophus reticulatus* comb. nov. showing variation in dorsal and ventral coloration. From left to right, first and second rows: QCAZA 43767, 44487, 14834, 43085, 43758, 44588 (females), 30955 (male); third and fourth rows: QCAZA 46396, 30956, 14836, 44589, 7909, 20408, 44668, 44669, CORBIDI 12253, QCAZA 43665 (males). See S1 Table for locality data. All specimens are shown at the same scale.

The external morphology of the holotype of *Dendropsophus triangulum* and its type locality indicate that it belongs to clade H. The holotype of *D*. *triangulum* is an adult of unknown sex with SVL = 25 mm (BMNH 1947.2.23.88, reported as “BMNH 68.11.15.2” by Condit [47]; Fig 7A). It closely resembles individuals from clades C and H, which also have a brown mark on the head and nape (Figs 8 and 9). In the species description by Günther [48] the type locality is vaguely stated as “Brazil”, which is consistent with the distribution of clades C and H (Fig 2). We assign the binomen *D*. *triangulum* to clade H based on the presence of a triangular mark (an isosceles triangle with its base in the interorbital area and its apex on the mid-dorsum) which we only observed in clade H. Clade C has been traditionally referred as “*D*. *triangulum*” (e.g. [3,4,9–12,15,16,19–21,49,50]; but see [7,8,14,17,18]). However, clade C differs from the holotype of *D*. *triangulum* in having one to several small and round dorsal marks instead of a single, large triangular mark (Figs 7A vs. 9).

*Hyla reticulata* Jiménez de la Espada, 1870 was synonymized under *Dendropsophus triangulum* (Günther, 1869) by Duellman [9]. The holotype (Fig 7B) is an adult male (MNCN 3474, reported as “MNCN 329” by Duellman [51]) with two mid-dorsal rows of small, round marks and similar rows on the flanks and dorsal surfaces of the limbs (Fig 7B). This color pattern resembles individuals from clade C (Fig 9) and is absent in *D*. *leucophyllatus* (Clade B) and *D*. *triangulum* (Clade H). The type locality of *Hyla reticulata* is “Río Napo, Provincia Napo” lies within the distribution of clades C and H (Fig 2). Based on this evidence, we remove the name *Hyla reticulata* from its synonymy with *D*. *triangulum* and assign it to clade C with the binomen *Dendropsophus reticulatus* comb. nov.

#### Taxonomic status of *Hyla frontalis*, *Hyla favosa*, *Hyla membranacea*, *Hyla laynei* and *Hyla oliveae*

*Hyla frontalis* Daudin, 1800 and *H*. *favosa* Cope, 1885 were synonymized under *Dendropsophus leucophyllatus* (Beireis, 1783) by Daudin [52] and Titus and Duellman [24], respectively. The holotype of *Hyla frontalis* (MNHNP 4868) could not be examined; nevertheless its type locality “Surinam” suggests that it belongs to *D*. *leucophyllatus* sensu stricto, which appear to be the only species of the *D*. *leucophyllatus* species group present in Surinam. In contrast, the distribution of *D*. *leucophyllatus* sensu stricto does not overlap with the type locality “Pebas, Upper Amazon” of *H*. *favosa*. Some specimens from clades A, B, E, and H resemble the description of *Hyla favosa* (a fine network of lines over the entire dorsum), but localities of clade H (*Dendropsophus triangulum*) are the closest to the type locality (Region of Loreto; Fig 2). Thus, we remove it from its synonymy with *D*. *leucophyllatus* and consider it a junior synonym of *D*. *triangulum*.

**Fig 10:**
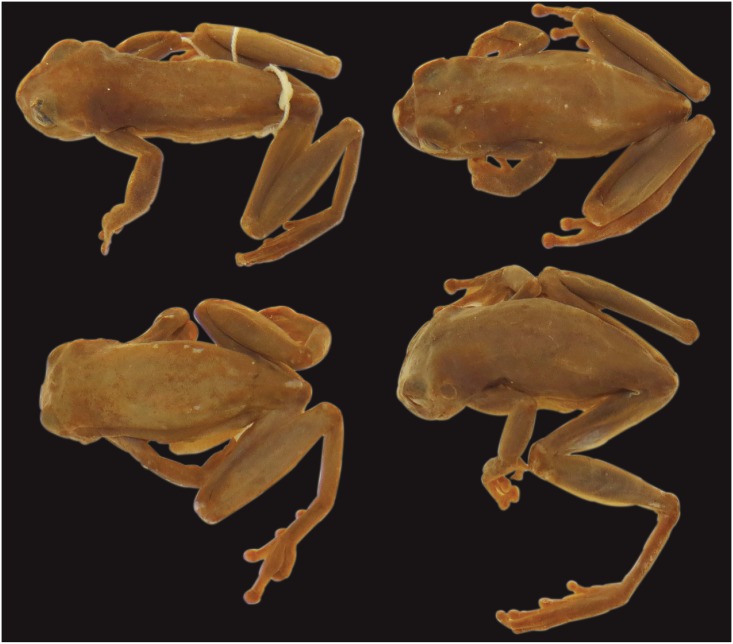
Dorsal views of the syntypes of *Hyla membranacea* (NHRM 1961; females).

*Hyla membranacea* Andersson, 1945 and *Hyla laynei* Goin, 1957 were synonymized under “*Hyla triangulum*” (= *Dendropsophus reticulatus*) by Duellman [9]. The syntypes of *Hyla membranacea* (NHRM 1961; four females with uniform brown dorsum; Fig 10) and the descriptions and pictures of *H*. *laynei* by Goin [53] resemble *D*. *reticulatus* comb. nov. (clade C; Fig 9). The type localities of *Hyla membranacea* “Río Pastaza, Watershed” and *H*. *laynei* “near Leticia, Amazonas Comisaria, Colombia” support this designation because they overlap with the distribution of *D*. *reticulatus* comb. nov. (clade C; Fig 2). Therefore, we consider *Hyla membranacea* and *H*. *laynei* junior synonyms of *Dendropsophus reticulatus* (Jiménez de la Espada, 1870) comb. nov.

*Hyla oliveae* Cochran and Goin, 1970 was considered a junior synonym of *Dendropsophus triangulum* by Duellman [9]. The holotype of *Hyla oliveae* (FSM 8555; type locality “Leticia, Amazonas, Colombia”) could not be examined. According to the original description by Cochran and Goin [54], *H*. *oliveae* is characterized by having bright gold spots on the dorsum, which disappeared in preservative. This character distinguishes it from *Dendropsophus leucophyllatus*, *D*. *triangulum*, *D*. *reticulatus* comb. nov. and the new species described herein. Thus, it may represent a valid species but examination of the type material and collections at the type locality are needed to determine its status.

### Systematic accounts

#### *Dendropsophus leucophyllatus* (Beireis 1783)

*Rana leucophyllata* Beireis, 1783:182. Type material not designated and likely lost according to Böhme [45]. Type locality “Surinam”.

*Hyla frontalis* Daudin, 1800. Holotype MNHNP 4868 by original designation. Type locality “Surinam”.

**Common names**. Standard English name: Beireis’ treefrog [55]. Proposed standard Spanish name: ranita de hoja blanca.

**Neotype** (Fig 6). MNHN 2015.129 (field no. AF 0606), adult male from French Guiana, Municipality of Sinnamary (5.3734° N, 53.0975° W), 49 m above sea level, collected by Antoine Fouquet and Jean-Pierre Vacher on 10 March 2012.

**Fig 11:**
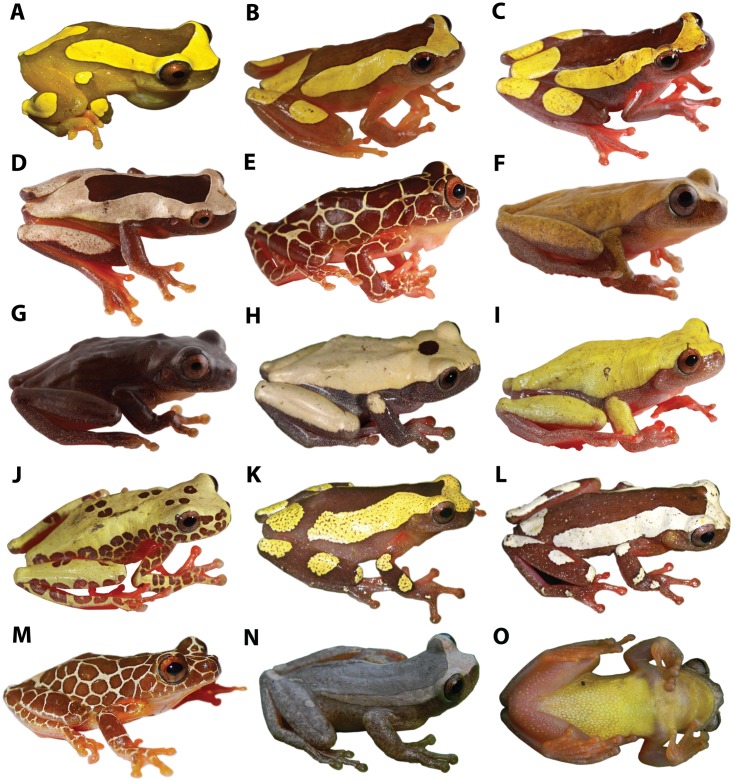
Dorsolateral and ventral views of the *D*. *leucophyllatus-triangulum* complex. **A** *D*. *leucophyllatus*, MNHN2015.134 (SVL = 26.8 mm), adult male; **B–E** *D*. *triangulum*, QCAZA 44291 (SVL = 31.3 mm), adult male, QCAZ 44293 (SVL = 41.3 mm), QCAZA 44456 (SVL = 40.2 mm), adult females, QCAZA 44539 (SVL = 33.3 mm), adult male; **F–J** *D*. *reticulatus* comb. nov., QCAZA 48711 (SVL = 26.5 mm), QCAZA 48701 (SVL = 26.5 mm), adult males, QCAZA 43084 (SVL = 31.6 mm), QCAZA 43758 (SVL = 34.2 mm), QCAZA 43767 (SVL = 35.7 mm), adult females; **K–M** *D*. *arndti* sp. n., SMF 88389 (holotype, SVL = 28.8 mm), SFM 88388 (SVL = 30.7 mm), SFM 88391 (SVL = 31.5 mm), adult males; **N–O** *D*. vraemi sp. n., CORBIDI 9614 (holotype, SVL = 26.6 mm), adult male. See S1 Appendix for locality data.

**Diagnosis**. *Dendropsophus leucophyllatus* (Figs 11A and 6) is characterized by: (1) mean SVL 29.7 mm in males (range 26.8–32.3; *n* = 12), 37.9 mm in females (range 36–39.8; *n* = 2); (2) axillary membrane reaching arm halfway to elbow; (3) basal webbing on fingers; (4) webbing on feet; (5) palmar tubercle single; (6) pectoral patches moderate; (7) dorsal coloration varying from brown to dark brown with white or bright yellow (in life) dorsolateral bands that extend to the tip of the snout; a white or bright yellow (in life) elliptical sacral mark shaped like an elongated leaf; (8) two white to bright yellow (in life) long bands on the dorsal surfaces of the shanks (sometimes fused and covering the shank completely); (9) some individuals with a reticulated color pattern; (10) in life, webbing and ventral surfaces from red to orange or pink; (11) in life, iris dull bronze or coppery bronze.

**Description of the neotype**. Adult male, SVL 29.2 mm, foot length 12.8 mm, head length 8.6 mm, head width 10.3 mm, eye diameter 3.3 mm, tympanum diameter 1.6 mm, tibia length 15 mm, femur length 15.4 mm, arm length 6.5 mm, eye-nostril distance 2.5 mm, body about as wide as head, head broader than long; snout short and rounded in dorsal view, truncate in profile; distance from nostril to eye shorter than diameter of eye; canthus rostralis scarcely distinct, rounded; loreal region plain; internarial region subtly depressed; nostrils slightly protuberant, directed posterolaterally; interorbital area flat; eye large, protuberant; diameter of eye 2.1 times diameter of tympanic annulus; tympanum concealed beneath skin; tympanic annulus visible below skin, ovoid, longer dorsoventrally and concealed dorsally by supratympanic fold, separated from eye by ca. 100% of its diameter; faint supratympanic fold, extending posteriorly from posterior corner of eye to anterior border of arm insertion. Arm slender, axillary membrane reaching halfway to elbow; relative length of fingers I < II < IV < III; fingers bearing large oval discs, that of third finger about three fourths of tympanum diameter; subarticular tubercles prominent, round to ovoid, single; distal tubercle on finger IV bifid; supernumerary tubercles present; palmar tubercle indistinct; prepollical tubercle large, flat, elliptical; prepollex elliptical, enlarged; nuptial excrescences absent; webbing formula of fingers I basal II1^1^/_2_−2^1^/_2_III2^2^/_3_−2^-^IV. Hindlimbs moderately long; toes bearing discs slightly wider than long, smaller than those of fingers; relative length of toes I < II < V < III < IV; outer metatarsal tubercle poorly defined, small, round; inner metatarsal tubercle large, elongated and elliptical; subarticular tubercles single, round, flat; supernumerary tubercles restricted to the soles; webbing formula of toes I1–1^1^/_2_II1^+^—2III1^+^—2IV2^+^—1^+^V. Two glandular patches on the chest posterior to the clavicle, separated from each other by about half their width. Skin on dorsum, head, and dorsal surfaces of limbs smooth; skin on flanks smooth with weak longitudinal wrinkles posterior to the arm; skin on venter coarsely granular except for the pectoral patches which are finely granular; skin on ventral surfaces of head and thighs granular, except for the vocal sac in the gular area which has longitudinal wrinkles; skin of shanks smooth. Cloacal opening at the level of upper edges of thighs; short, simple cloacal sheath covering cloacal opening. Tongue broadly cordiform, free laterally and posteriorly, widely attached to mouth floor; vomerine odontophores situated between choanae, in two slightly angled series, not in contact with each other, about as wide as choanae, each bearing 2 vomerine teeth; choanae round.

*Color of neotype in preservative* (Fig 6). Dorsum dark brown with white dorsolateral bands on each side of the body reaching the anterior portion of the sacrum; the dorsolateral bands converge on a white triangle on the tip of the head with its base in the interorbital area and its apex between the nostrils; a white elliptical leaf-shaped mark, separated from the dorsolateral bands, is present on the sacrum; the tip of the snout, sides of the head, supra cloacal region, flanks, and dorsal surfaces of the limbs are dark brown; two white rounded spots are present on the dorsal surfaces of the forearm, two white long ovoid bands on the shank; venter creamy white; ventral surfaces of limbs and webbing yellowish white; pectoral patches cream.

**Variation**. Coloration in this section refers to preserved specimens unless otherwise noticed. Variation in dorsal and ventral coloration of preserved specimens is shown in Fig 6. Background dorsal coloration varies from brown (e.g., MNHN2015.131, MNHN2015.134) to dark brown (e.g., MNHN2015.127–28) with a white triangle on the tip of the head with its base in the interorbital area and its apex between the nostrils. Dorsolateral white broad bands are present on each side of the body, reaching the anterior portion of the sacrum (e.g., MNHN2015.132–34). A white elliptical leaf-shaped sacral mark is present, which is separated from the dorsolateral bands; in some individuals a perpendicular brown fine line crosses the sacral mark (e.g., MNHN2015.134). The dorsal surfaces of the limbs have white rounded spots (one or two on each forearm) and long ovoid bands (one to three on each shank); in some specimens the bands are fused and cover the dorsal surface of the shank (e.g., MNHN2015.127–28, MNHN2015.132, MNHN2015.134). The thighs are immaculate with or without a thin brown stripe along the dorsal surface. Some specimens have a very different pattern. It consists of dark round marks over the entire dorsum, sides of the head, and dorsal surfaces of the limbs on a white or bright yellow (in life) background. The light background forms a contrasting reticulum. This reticulated pattern was observed only in populations of the eastern part of the Guianas, east of the Approuagues River (e.g., localities of Serra do Navio, Macapa, Trois-Sauts and Toponowini). Ventral areas, webbing and discs are creamy white (e.g., MNHN2015.131) or yellowish (e.g., MNHN2015.127–28) with cream pectoral patches (e.g., MNHN2015.134).

**Coloration in life** (based on digital photographs; Fig 11A). Dorsal coloration is the same as in preserved specimens (previous section) except for the color of the clear areas, which varies from white to bright yellow. The tip of the nose, sides of the head, supracloacal region and flanks vary from brown to dark brown. At night, the ventral surfaces of the limbs, anterior and posterior surfaces of the thighs and webbing are pink; these surfaces changes to red in the day. The vocal sac is yellow, the belly is creamy white and the iris is dull bronze to coppery bronze.

**Fig 12:**
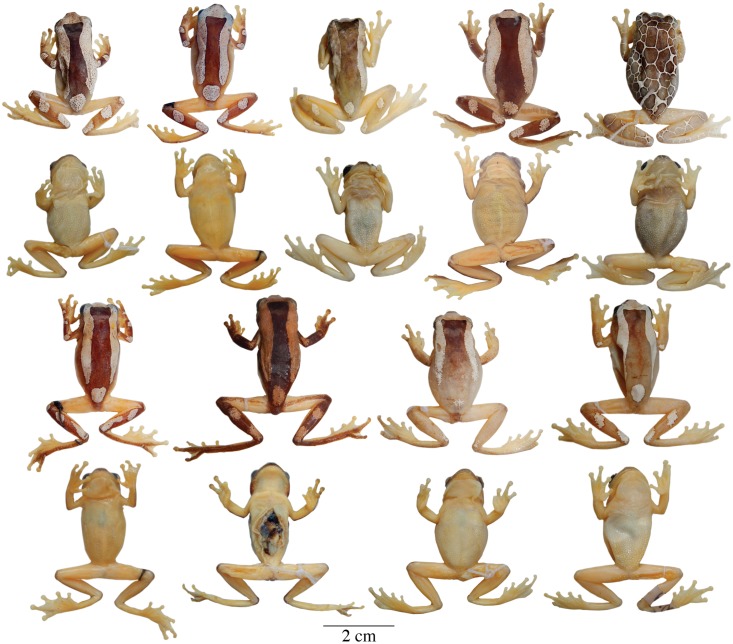
Adult preserved specimens of *Dendropsophus arndti* sp. n. showing variation in dorsal and ventral coloration. From left to right, first and second rows: SMF 88389 (holotype), 88387, 88392, 88383, 88391 (males); third and fourth rows: SMF 88388, 88386, 88385, 88390 (males). See type-series for locality data. All specimens are shown at the same scale.

**Comparisons with other species**. *Dendropsophus leucophyllatus* is most similar to *D*. *arndti* sp. n. and *D*. *triangulum*. It differs from both species by the presence of a clear elliptical leaf-shaped sacral mark (round to ovoid clear mark with irregular edges and scattered small spots in *D*. *arndti* (Fig 12); round clear mark connected to dorsolateral clear bands in *D*. *triangulum*) and by having shorter Type I notes in the advisement call (Table 5). *Dendropsophus leucophyllatus* differs from *D*. *reticulatus* comb. nov. by its larger size (mean male SVL = 29.7, SD = 1.7, *n* = 12; *D*. *reticulatus* mean male SVL = 23.8, SD = 1.5, *n* = 185; differences are significant: *t* = -10.77, df = 12, *P* < 0.001; Table 3) and in advertisement call (lower frequency bandwidth and dominant frequency). In addition, *D*. *leucophyllatus* has clear dorsolateral bands that extend to the tip of the snout and a distinctive leaf-shaped sacral mark (uniform dorsal color pattern, sometimes punctuated by one or several small round marks in *D*. *reticulatus*).

**Table 5:** Descriptive statistics for advertisement calls parameters of species of the *D*. *leucophyllatus-triangulum* complex.

Species	Call duration	Number of notes per call	Rise time of the call	Number of pulses per call	Type I note duration	Number of pulses of the Type I note	Rise time of Type I note	Type II note duration	Number of pulses of the Type II note	Distance between notes	Average of the dominant frequency call	Frequency bandwidth
***D*. *arndti* sp. n.** (n = 7)	0.19 ± 0.02 (0.16–0.23)	1	0.12 ± 0.08 (0.03–0.29)	17.44 ± 1.23 (16–19)	0.2 ± 0.02 (0.16–0.23)	17.44 ± 1.23 (15.6–19.0)	0.12 ± 0.08 (0.03–0.29)	–	–	–	2655.37 ± 169.42 (2416.3–2876.1)	487.67 ± 75.06 (353.1–570.6)
***D*. *leucophyllatus*** (n = 9)	0.17 ± 0.02 (0.16–0.22)	2.10 ± 0.16 (2–3)	0.08 ± 0.04 (0.05–0.15)	16.49 ± 1.94 (14–21)	0.1 ± 0.01 (0.08–0.11)	12.19 ± 0.91 (10.8–13.8)	0.06 ± 0.01 (0.04–0.08)	0.027 ± 0.007 (0.01–0.04)	3.85 ± 0.45 (3.60–4.25)	0.046 ± 0.006 (0.03–0.05)	2748.69 ± 162.89 (2453.9–2914.1)	530.32 ± 113.7 (301.4–663.2)
***D*. *reticulatus* comb. nov.** (n = 4)	0.35 ± 0.05 (0.27–0.39)	4.05 ± 0.33 (3–5)	0.18 ± 0.01 (0.18–0.19)	30.39 ± 2.75 (26–33)	0.1 ± 0.01 (0.08–0.12)	13.55 ± 1.18 (12.6–17)	0.08 ± 0.02 (0.06–0.1)	0.039 ± 0.004 (0.03–0.04)	5.56 ± 0.47 (5–6)	0.042 ± 0.004 (0.03–0.04)	2992.39 ± 100.8 (2888.3–3128.6)	705.98 ± 112.5 (574.2–843.7)
***D*. sp D** (n = 4)	0.21 ± 0.02 (0.19–0.24)	1.10 ± 0.20 (1–2)	0.15 ± 0.01 (0.13–0.16)	18.43 ± 1.21 (17–21)	0.21 ± 0.02 (0.19–0.24)	18.03 ± 0.45 (17.5–18.6)	0.15 ± 0.01 (0.13–0.16)	0.004 ± 0.008 (0–0.016)	0.4 ± 0.80 (0–1.6)	0.001 ± 0.003 (0–0.006)	2493.64 ± 197.32 (2213.1–2652.9)	483.69 ± 101.13 (335.9–565.2)
***D*. sp F** (n = 2)	0.22 ± 0.02 (0.21–0.24)	2.50	0.09	17	0.11 ± 0.01 (0.10–0.12)	12.88 ± 1.59 (11.7–14)	0.08	0.03 ± 0.002 (0.02–0.03)	3.50	0.047 ± 0.005 (0.04–0.05)	2660.63 ± 48.05 (2626.6–2694.6)	581.39 ± 395.86 (301.4–861.3)
***D*. sp G** (n = 5)	0.28 ± 0.05 (0.25–0.35)	3.28 ± 0.50 (2–4)	0.09 ± 0.02 (0.06–0.11)	24.40 ± 2.87 (21–28)	0.11 ± 0.01 (0.10–0.12)	13.92 ± 0.66 (13.2–14.6)	0.08 ± 0.02 (0.06–0.10)	0.033 ± 0.003 (0.034–0.037)	4.5 ± 0.35 (4.13–4.93)	0.041 ± 0.004 (0.03–0.04)	2570.90 ± 99.68 (2431.8–2703.4)	471.16 ± 145.27 (361.7–684.7)
***D*. *triangulum*** (n = 11)	0.26 ± 0.04 (0.22–0.36)	2.33 ± 0.52 (2–4)	0.13 ± 0.03 (0.09–0.17)	20.92 ± 3.39 (17–29)	0.15 ± 0.02 (0.12–) 0.18	15.72 ± 2.24 (13–20.2)	0.11 ± 0.02 (0.08–0.16)	0.034 ± 0.007 (0.023–0.042)	3.75 ± 0.52 (3–4.5)	0.051 ± 0.007 (0.04–0.06)	2456.43 ± 278.91 (2101–3070.7)	478.34 ± 113.54 (281.2–646)

The *n* values indicate the number of males analyzed. Mean ± SD is given with range in parentheses. See Table 1 for a description of each parameter. Call vouchers: *Dendropsophus arndti* sp. n. (SMF 88384, 88388–90, 88393), *D*. *leucophyllatus* (MZUSP 950152–53, 950156–58, 950161), *D*. *reticulatus* comb. nov. (KU 126484–85, QCAZA 367–69), *D*. sp. D (MZUSP 950253–54), *D*. sp. F (MZUSP 950163–64), *D*. sp. G (MZUSP 930042, 930045–47, 930050) and *D*. *triangulum* (MZUSP 950187, 950192, KU 126421, 143152–54, QCAZA 44290).

*Dendropsophus leucophyllatus* males (26.8–32.3 mm) are larger (non-overlapping ranges) than males of *D*. *manonegra* (22.7–25.1 mm [8]) and *D*. *rossalleni* (19–22.3 mm [56]). *Dendropsophus leucophyllatus* can be further distinguished by having dorsolateral wide bands (dorsolateral bands are thin in *D*. *manonegra*, *D*. *sarayacuensis*, and *D*. *vraemi* sp. n., and absent in *D*. *rossalleni*). *Dendropsophus manonegra* has, in life, bluish-black coloration on digits, webbing, axillary membranes, groin and hidden surfaces of arms and legs [8] (pink or red in *D*. *leucophyllatus*); *D*. *rossalleni* has pale creamy white spots anteromedially on the upper eyelids and adjacent head [56] (pale mark on the head extends to the snout, the scapular marks are larger and more diagonal, and always depart from behind the orbits in *D*. *leucophyllatus*, Fig 6); *D*. *sarayacuensis* has frayed dorsolateral bands and rounded spots with irregular borders on the dorsal surfaces of the limbs (borders are smooth and well defined in *D*. *leucophyllatus*); *D*. *bifurcus* lacks long ovoid bands on the shanks (present in *D*. *leucophyllatus*). *Dendropsophus leucophyllatus* differs from the holotype of *D*. *salli* (MNK-A 8445; see Discussion section) in having a medial leaf-shaped clear sacral mark (medial rhomboidal sacral mark in *D*. *salli*; Fig. 1 in [10]).

**Calls** (Fig 4A; Table 5). The advertisement call of *Dendropsophus leucophyllatus* consists of one pulsed trill note (Type I, mean duration of 0.10 s and 10–14 pulses/note; Table 5) followed by one or two secondary notes (Type II) with less duration and pulses than the Type I note (mean 0.027 s and 3–5 pulses/note). The advertisement call has a mean duration of 0.17 s (SD = 0.02; range = 0.16–0.22 s; *n* = 9) with an average dominant frequency of 2748.6 Hz (SD = 162.89), mean rise time of 0.08 s (SD = 0.04) and mean frequency bandwidth of 530.3 Hz (SD = 113.7). Other call parameters are listed in Table 5.

The aggressive call (Fig 4B; Table 6) consists of 3 to 5 Type II notes with more duration (mean 0.25 s, SD = 0.05) than the advertisement call (duration mean 0.17 s, SD = 0.002). The rise time and mean distance between notes are 0.15 s (SD = 0.15) and 0.036 s (SD = 0.004) respectively. The average dominant frequency is 2615.8 Hz (SD = 196.39) and the frequency bandwidth is 423.2 Hz (SD = 64.73). Other call parameters are listed in Table 6.

**Table 6:** Descriptive statistics for aggressive calls parameters of species of the *D*. *leucophyllatus-triangulum* complex.

Species	Call duration	Number of notes per call	Rise time of the call	Number of pulses per call	Type II note duration	Number of pulses of the Type II note	Distance between notes	Average of the dominant frequency call	Frequency bandwidth
***D*. *arndti* sp. n.** (n = 7)	0.14 ± 0.01 (0.12–0.16)	3.03 ± 0.08 (3–4)	0.07 ± 0.02 (0.03–0.1)	11.60 ± 1.13 (12–13)	0.035 ± 0.006 (0.024–0.042)	3.84 ± 0.40 (3.13–4.33)	0.02 ± 0.011 (0.011–0.045)	2668.76 ± 111.64 (2485.2–2830.3)	423.26 ± 64.73 (335.9–516.8)
***D*. *leucophyllatus*** (n = 5)	0.25 ± 0.05 (0.18–0.34)	3.96 ± 0.64 (3–5)	0.15 ± 0.15 (0.04–0.4)	19.96 ± 4.14 (13–25)	0.033 ± 0.004 (0.027–0.038)	5.01 ± 0.36 (4.6–5.5)	0.036 ± 0.004 (0.029–0.040)	2615.82 ± 196.39 (2326.6–2811.9)	548.96 ± 101.28 (469.4–689.1)
***D*. *reticulatus* comb. nov.** (n = 5)	0.59 ± 0.11 (0.48–0.71)	4.11 ± 0.72 (3–5)	0.38 ± 0.13 (0.24–0.51)	22.11 ± 5.71 (16–29)	0.038 ± 0.003 (0.035–0.041)	5.32 ± 0.53 (4.5–5.8)	0.05 ± 0.004 (0.045–0.055)	2832.05 ± 106.24 (2675.2–2924.1)	735.46 ± 120.51 (600–875)
***D*. sp D** (n = 5)	0.16 ± 0.01 (0.14–0.18)	3.48 ± 0.5 (3–4)	0.10 ± 0.01 (0.09–0.12)	14.01 ± 0.74 (12–15)	0.035 ± 0.005 (0.031–0.042)	4.11 ± 0.57 (3.5–4.9)	0.014 ± 0.002 (0.01–0.017)	2387.89 ± 157.4 (2141–2538.7)	301.04 ± 52.11 (215.3–355.3)
***D*. sp G** (n = 2)	0.44 ± 0.1 (0.37–0.52)	6.42 ± 1.53 (5–8)	0.13 ± 0.04 (0.10–0.16)	29.42 ± 4.36 (26–33)	0.036 ± 0.003 (0.034–0.039)	4.64 ± 0.44 (4.3–4.9)	0.037	2524.94 ± 172.33 (2403–2646.7)	386.72 ± 8.89 (380.4–393)
***D*. *triangulum*** (n = 5)	0.24 ± 0.03 (0.19–0.27)	4.25 ± 0.23 (4–5)	0.14 ± 0.02 (0.11–0.16)	19.81 ± 1.66 (17–22)	0.036 ± 0.005 (0.034–0.041)	4.66 ± 0.35 (4.3–5.1)	0.027 ± 0.006 (0.018–0.033)	2411.74 ± 349.62 (2085–2968.3)	427.4 ± 187.34 (281.2–689.1)

The *n* values indicate the number of males analyzed. Mean ± SD is given with range in parentheses. See Table 1 for a description of each parameter. Call vouchers: *Dendropsophus arndti* sp. n. (SMF 88384, 88388–90), *D*. *leucophyllatus* (MZUSP 950152, 950161), *D*. *reticulatus* comb. nov. (KU 126484, 143145, 143179, QCAZA 49174), *D*. sp. D (MZUSP 950253–54), *D*. sp. G (MZUSP 930045, 930050) and *D*. *triangulum* (MZUSP 950192).

**Distribution and ecology**. *Dendropsophus leucophyllatus* has been recorded (based on DNA sequences and specimens listed in the S1 Appendix and S3 Table) in northern Brazilian Amazonia, Suriname, Guyana and French Guiana (Fig 2). Known localities range in elevation from sea level (Kaw) to 400 m (Grande Montagne Tortue).

Specimens of *Dendropsophus leucophyllatus* were found in permanent or semipermanent ponds along roads or in pristine forest, large coastal swamps, and at the edges of forest savannas, perching from a few cm above the water to several meters high.

Vegetation types at known localities include Guianan Moist Forest for Suriname and French Guiana localities, and Madeira-Tapajós Moist Forest, Uatuma-Trombetas Moist Forests and Japurá-Solimões-Negro Moist Forests for the Brazilian localities (vegetation types according to World Wildlife Fund [57]).

**Conservation status**. The distribution polygon of *Dendropsophus leucophyllatus* has 382,451 km^2^. Because its geographic range is large and includes extensive areas of undisturbed forest and protected areas, we recommend that *D*. *leucophyllatus* is assigned to the Red List category Least Concern.

#### *Dendropsophus triangulum* (Günther, 1869)

*Hyla triangulum* Günther, 1869:489. Holotype BMNH 1947.2.23.88. Type locality “Brazil”.

*Hyla favosa* Cope, 1885:95. Holotype ANSP 11483, an adult male from “Pebas, Upper Amazon”, Region of Loreto, Peru.

**Common name**. Proposed standard English name: Triangle treefrog [55]. Proposed standard Spanish name: ranita triangular.

**Diagnosis**. A member of the genus *Dendropsophus* characterized by: (1) mean SVL 31.7 mm in males (range 28.6–34.4; *n* = 26), 40 mm in females (range 37.5–41.9; *n* = 5); (2) axillary membrane reaching arm halfway to elbow; (3) basal webbing on fingers; (4) webbing on feet; (5) palmar tubercle single; (6) pectoral patches moderate; (7) dorsal coloration white or bright yellow (in life) with a distinctive hour-glass-shaped dark brown mark on the back, in some specimens only half of the hourglass is present; (8) white or bright yellow (in life) variation between long ovoid bands and rounded spots on the dorsal surfaces of the limbs (one to two on the forearm and one to three on the shank); (9) some individuals with a reticulated color pattern; (10) in life, webbing and ventral surfaces varying from pale orange or yellowish orange; (11) in life, iris dull bronze to orange bronze.

**Variation**. Coloration in this section refers to preserved specimens unless otherwise stated. Variation in dorsal and ventral coloration of preserved specimens is shown in Fig 8. A dark brown mid-dorsal area is always present but varies extensively in size (Fig 8). In some individuals, the dark area is reduced to an isosceles triangle on the nape or to a longitudinal band from the head to half the body length (e.g., QCAZA 35504, 44467, CORBIDI 12194). The tip of the nose, sides of the head, supra-cloacal region, flanks, and dorsal surfaces of the limbs are brown to dark brown. Background dorsal coloration of the limbs varies from brown (e.g., QCAZA 44293) to dark brown (e.g., QCAZA 44458) with white rounded spots on the dorsal surfaces of the forearms (one or two for each one) and similar spots or long ovoid bands on the shanks (two discrete or fused bands that may cover the shank completely; e.g., QCAZA 35504). The thighs are immaculate with or without a thin brown stripe along the dorsal surface. Some specimens have a reticulated pattern (a fine network of white lines on a dark brown to brown background; e.g., QCAZA 44539). The interspaces are as large or larger than the eye, except on the sides of the head and body, where they are smaller. Ventral areas, webbing and discs vary from creamy white to yellowish white with cream pectoral patches.

**Coloration in life** (based on digital photographs; Fig 11B–11E). Dorsal and lateral coloration is the same as in preserved specimens (previous section) except for the color of the clear areas, which varies from white to bright yellow. In some individuals there are scattered minute bright yellow spots on the dark dorsal areas (e.g., QCAZA 44292). At night the ventral surfaces of the limbs, anterior and posterior surfaces of thighs and webbing are yellowish orange; these surfaces are pale orange in the day. The vocal sac is yellowish orange (e.g., QCAZA 55396), the belly is creamy white (e.g., QCAZA 44290) to yellowish orange (e.g., QCAZA 44293) and the iris is dull bronze to orange bronze.

**Comparisons with other species**. *Dendropsophus triangulum* is most similar to *D*. *arndti* sp. n. and *D*. *leucophyllatus*, but it can be distinguished by having a brown hour-glass-shape or isosceles triangular mark on the nape (Fig 8). Some individuals have a white or bright yellow rounded sacral mark but differ because in *D*. *leucophyllatus* the mark is leaf-shaped and in *D*. *arndti* sp. n. is rounded with irregular edges and scattered small spots.

*Dendropsophus triangulum* differs from *D*. *reticulatus* comb. nov. in advertisement call (Fig 4C vs. 4E) and by its larger size (mean male SVL = 31.7, SD = 1.5, *n* = 26; *D*. *reticulatus* mean male SVL = 23.8, SD = 1.5, *n* = 185; differences are significant: *t* = 24.9, df = 33, *P* < 0.001; Table 3). Most specimens of *D*. *reticulatus* have a uniform dorsal color pattern, sometimes punctuated by one or several brown marks. The reticulated color morph is shared by *D*. *triangulum*, *D*. *leucophyllatus*, *D*. *arndti* sp. n. and *D*. *reticulatus*, but *D*. *triangulum* differs from *D*. *reticulatus* and *D*. *leucophyllatus* in the size of the interspaces (narrow and rounded in *D*. *reticulatus*; compared QCAZA 44539, Fig 8 vs. CORBIDI 12253, Fig 9).

*Dendropsophus triangulum* males (28.6–34.4 mm) are larger than males of *D*. *bifurcus* (23–28 mm [21]), *D*. *manonegra* (22.7–25.1 mm [8]), *D*. *rossalleni* (19–22.3 mm [56]) and *D*. *vraemi* sp. n. (25.1–27.6 mm). The species can be distinguished from congeners by its dorsolateral wide bands (dorsolateral thin bands in *D*. *manonegra*, *D*. *sarayacuensis*, and *D*. *vraemi* sp. n., and absent in *D*. *rossalleni*). *Dendropsophus bifurcus* lacks extensive ovoid bands on the shanks (present in *D*. *triangulum*); *D*. *manonegra* has, in life, bluish-black coloration on digits, webbing, axillary membranes, groin and hidden surfaces of arms and legs [8] (yellowish orange or pale orange in *D*. *triangulum*); *D*. *rossalleni* has pale creamy white spots on the anteromedial parts of the upper eyelids and adjacent part of the head [56] (a pale triangular mark on the tip of the head with its base in the interorbital area and its apex between the nostrils in *D*. *triangulum*; Fig 8); and *D*. *sarayacuensis* has frayed dorsolateral bands and rounded spots with irregular borders on the dorsal surfaces of the limbs (smooth and well defined in *D*. *triangulum*). The holotype of *D*. *salli* (MNK-A 8445; see Discussion section) differs from *D*. *triangulum* in having a medial rhomboidal sacral mark (Fig. 1 in [10]) (clear rounded mark in *D*. *triangulum*).

**Calls** (Fig 4C; Table 5). The advertisement call consists of one pulsed trill note (Type I, mean duration of 0.15 s and 13–21 pulses/note) followed by one to three secondary notes (Type II) with less duration and pulses (mean 0.034 s; and 3–5 pulses/note). Mean duration of the call is 0.26 s (SD = 0.04; range = 0.22–0.36 s; *n* = 11) with an average dominant frequency of 2456.43 Hz (SD = 278. 91), mean rise time of 0.13 s (SD = 0.03) and mean frequency bandwidth of 494 Hz (SD = 146.95).

The aggressive call (Fig 4D; Table 6) consists of 4–5 Types II pulsed notes with less distance between notes (mean 0.027, SD = 0.006) and more notes/call (mean 4.25, SD = 0.23) than the advertisement call (mean distances between notes 0.051, SD = 0.007; and mean 2.33 notes/call, SD = 0.52). The aggressive calls have a mean duration of 0.24 s (SD = 0.03) with an average dominant frequency of 2411.74 Hz (SD = 349.62), mean rise time of 0.14 s (SD = 0.02) and mean frequency bandwidth of 427.4 Hz (SD = 187.34). Other call parameters are listed in Tables 5 and 6.

**Distribution and ecology**. This species occurs in the Amazon basin of Ecuador (Orellana and Sucumbíos provinces), Peru (regions of Loreto and Cusco) and Brazil (states of Acre, Amazonas and Pará) (Fig 2). Localities with known elevation range vary between 34 and 387 m above sea level. The elevation of Malvinas (387 m) is the highest known, whereas Obidos (34 m) is the lowest. *Dendropsophus triangulum* has been reported by Moravec and Aparicio [58] in Nacebe, Bolivia; however the collected specimens according to the description resemble *D*. *reticulatus* comb. nov. (dorsal surfaces cream to yellow with the presence or absence of a small spot in interorbital position).

Specimens were found at night in pools in secondary forest, lakes, and swamps, perching on vegetation 100 to 420 cm above the ground. Vegetation types for Ecuadorian localities are: (1) Amazonian Lowland Evergreen Forest, characterized by high plant α-diversity and a canopy height of 30 m with emergent trees that reach 40 m, (2) Floodplain Lowland Forest of White-Waters, characterized by periodical flooding with white-waters from large rivers, with the vegetation reaching 35 m in height, and several horizontal strata of vegetation, and (3) Lowland Forest of Palms and black-waters characterized by a canopy height of 30 m with dense understory and dominance of the palm *Mauritia flexuosa*.

Vegetation types at localities in Peru and Brazil include Napo Moist Forest (Peru), Southwest Amazon Moist Forest (Peru), and Uatuma-Trombetas Moist Forests (Brazil), according to the World Wildlife Fund [57]).

**Conservation status**. The distribution polygon of *Dendropsophus triangulum* has 923,514 km^2^ and overlaps with protected areas and large regions of pristine forest. Within this area, 52,604 km^2^ (5.7%) of its habitat has been degraded by human activities. For these reasons we suggest to assign *D*. *triangulum* to the Red List category Least Concern.

#### *Dendropsophus reticulatus* (Jiménez de la Espada, 1870) comb. nov.

*Hyla reticulata* Jiménez de la Espada, 1870: 61. Holotype MNCN 3474, a male from “Río Napo, Provincia Napo”, Ecuador.

*Hyla membranacea* Andersson, 1945: 107. Syntypes NHRM 1961 (4 specimens).

Type locality “Rio Pastaza, Watershed”, eastern Ecuador.

*Hyla laynei* Goin, 1957: 61. Holotype FSM 8503. Type locality “near Leticia, Amazonas Comisaria”, Colombia.

**Common name**. Proposed standard English name: Reticulate treefrog. Proposed standard Spanish name: ranita reticulada.

**Diagnosis**. A member of the genus *Dendropsophus* characterized by: (1) mean SVL 23.8 mm in males (range 20–29.6; *n* = 185), 33.1 mm in females (range 28–39.7; *n* = 66); (2) axillary membrane reaching arm halfway to elbow; (3) basal webbing on fingers; (4) webbing on feet; (5) palmar tubercle single; (6) pectoral patches moderate; (7) dorsal coloration varying from brown to reddish brown, white or bright yellow (in life) with or without a varying number of dark brown round marks on the dorsum; (8) a white long ovoid band covering the dorsal surface of the shank; (9) some individuals with a reticulated color pattern; (10) in life, webbing and ventral surfaces varying from red to salmon pink; (11) in life, iris dull bronze to coppery bronze.

**Variation**. Coloration in this section refers to preserved specimens unless otherwise stated. Variation in dorsal and ventral coloration of preserved specimens is shown in Fig 9. Dorsal coloration varies from brown to reddish brown (e.g., QCAZA 14834) or white (e.g., QCAZA 43767) with or without one to many brown round marks on the dorsum. In few individuals there are small black spots scattered on the dorsum (e.g., QCAZA 14836, 30955, 43665). Dorsal surfaces of the shanks and sometimes forearms, hands and feet have the same color as the dorsum (e.g., QCAZA 14834, 43085, 44668) or are dark brown (e.g., QCAZA 43665, 44588). In some specimens there are one or two white rounded spots on the dorsal surfaces of the forearms (e.g., QCAZA 43665, 44589). The tip of the snout, sides of the head, supra cloacal region and flanks are brown. Duellman (1974) identified seven color morphs based on the number and distribution of the dorsal dark marks. We observed the same color patterns with some variations: (1) immaculate dorsum (e.g., QCAZA 14834); (2) one round mark on the occipital region (e.g., QCAZA 7909); (3) two round marks, one located on the mid-dorsal region and the other on the occipital region (e.g., QCAZA 43085); (4) a wide dark band from the head to the mid-body (e.g., QCAZA 44589); (5) three round marks, one on the head and two on the mid-body; (6) one or two rows of round marks mid-dorsally (e.g., QCAZA 30956, 44669); and (7) round marks over the entire dorsum, similar to the reticulated pattern (e.g., CORBIDI 12253). Color morphs 6 and 7 have a similar row of round marks on each flank where the dorsal and ventral background colors meet; round marks are also present on the dorsal surfaces of the limbs and side of the head. Dorsal surfaces of thighs are brown or immaculate with or without a thin brown stripe along the dorsal surface. Ventral areas, webbing, and discs vary from creamy white (e.g., QCAZA 43085) to yellowish white (e.g., QCAZA 7909) with cream pectoral patches.

**Coloration in life** (based on digital photographs; Fig 11F–11J). Dorsal surfaces vary from brown (e.g., QCAZA 43759) to dark brown (e.g., QCAZA 48701), white (e.g., QCAZA 43084), or bright yellow (e.g., QCAZA 43758) with or without dark brown marks (e.g., QCAZA 43759, 43767). The tip of the snout, sides of the head, supra-cloacal region and flanks are brown (e.g., QCAZA 43759), dark brown (e.g., QCAZA 12541) or grayish (e.g., QCAZA 43084). At night the ventral surfaces of the limbs, anterior, and posterior surfaces of thighs and webbing are salmon pink; these surfaces change to red by day. The vocal sac and the belly are reddish white (e.g., QCAZA 43084) or yellowish (e.g., QCAZA 48701); the iris is coppery bronze (e.g., QCAZA 43767).

**Comparisons with other species**. *Dendropsophus reticulatus* can be distinguished from *D*. *leucophyllatus*, *D*. *triangulum*, and *D*. *arndti* sp. n. by having a uniform dorsal color pattern, sometimes punctuated by one or several dark brown round marks (an elliptical leaf-shaped sacral mark in *D*. *leucophyllatus*; a rounded clear sacral mark with irregular edges and scattered small spots in *D*. *arndti* sp. n.; a brown hour-glass-shaped or isosceles triangular mark on the nape in *D*. *triangulum*; Figs 9 vs. 6, 8 and 12). *Dendropsophus reticulatus* further differs from these three species in advertisement call (Fig 4A, 4C, 4E and 4G) and by its smaller size (all *P* values for *t* tests < 0.001; Table 3). The digits, webbing, axillary membranes, groin, and hidden surfaces of arms and legs are reddish in *D*. *reticulatus* (orange in *D*. *triangulum* and *D*. *arndti* sp. n.) This color fades to cream in preservative and becomes indistinguishable among the three species. The reticulated color morph of *D*. *reticulatus* can be distinguished from that of other species by having a thicker reticulum (compare Figs 9 vs. 8 and 12).

*Dendropsophus reticulatus* is similar to *D*. *bifurcus* and *D*. *sarayacuensis*, but it differs in having, in life, an orange venter (red in *D*. *reticulatus*). They also differ in dorsal coloration, as *D*. *bifurcus* has clear dorsolateral bands on a dark background (absent in *D*. *reticulatus*), and *D*. *sarayacuensis* has a bicolored head (clear anterior to the orbits, contrasting dark posteriorly), which differs from the coloration of *D*. *reticulatus* (Fig 9). *Dendropsophus reticulatus* can be distinguished from *D*. *rossalleni*, *D*. *manonegra* and *D*. *vraemi* sp. n. by having an orange venter in life (dull cream in *D*. *rossalleni* [56]; white in *D*. *manonegra* [8]; and yellowish in *D*. *vraemi* sp. n.). They also differ in dorsal coloration as *D*. *rossalleni* has pale marks on the anteromedial parts of the upper eyelids and adjacent part of the head [56] and *D*. *manonegra* and *D*. *vraemi* sp. n. have clear thin dorsolateral bands (absent in *D*. *reticulatus*; Figs 9 vs. 13). The holotype of *D*. *salli* (MNK-A 8445; see Discussion section) differs from *D*. *reticulatus* in having a clear dorsolateral bands and a medial rhomboidal sacral mark in *D*. *salli* [10] (absent in *D*. *reticulatus*).

**Fig 13:**
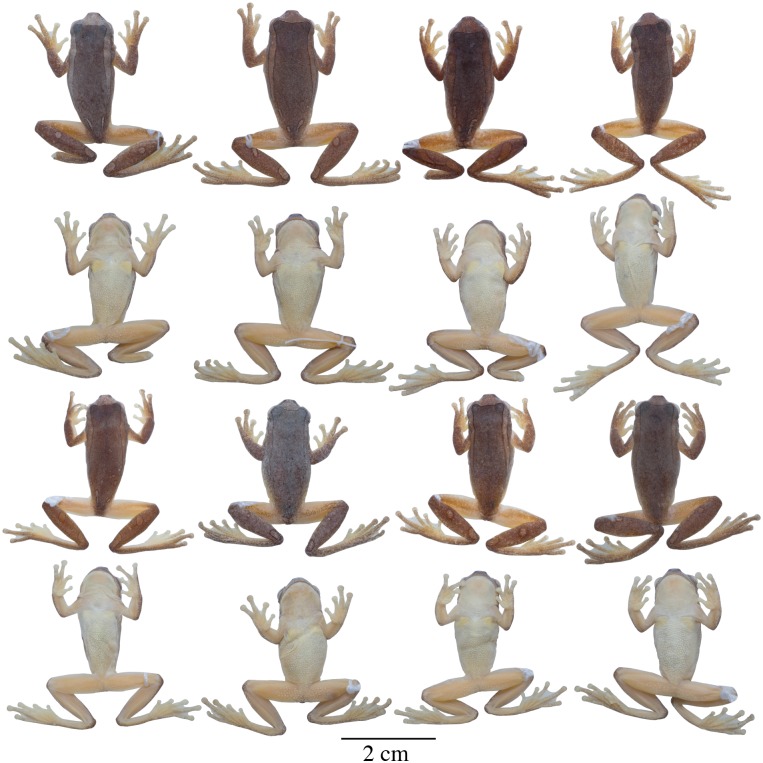
Adult preserved specimens of *Dendropsophus vraemi* sp. n. showing variation in dorsal and ventral coloration. From left to right, first and second rows: CORBIDI 9614 (holotype), 9640, 9613, 9644 (males); third and fourth rows: CORBIDI 9643, 9646, 9642, 9641 (males). See type-series for locality data. All specimens are shown at the same scale.

**Calls** (Fig 4E; Table 5). The advertisement call consists of one pulsed trill note (Type I, mean duration of 0.10 s, SD = 0.01 and 12–17 pulses/note) followed by two to four secondary notes (Type II) with less duration and pulses (mean 0.039 s, SD = 0.004 and 5–6 pulses/note). In most of the calls, a multiple pulse introductory note is present at the beginning of the call (mean duration 0.29 s, SD = 0.08) and the first two pulses of the Type II notes are more separated (mean 0.010 s, SD = 0,0003) from the rest of the pulses (mean 0.0023 s, SD = 0.0004). The mean distance between notes is 0.042 (SD = 0.004). The advertisement call has a mean duration of 0.35 s (SD = 0.05; range = 0.27–0.39 s; *n* = 4) with an average dominant frequency of 2992.3 Hz (SD = 100.80), mean rise time of 0.18 s (SD = 0.01), and mean frequency bandwidth of 705.98 Hz (SD = 112.50).

The aggressive call (Fig 4F; Table 6) consists of 3–5 Type II notes with more duration (mean 0.59 s, SD = 0.11) and mean rise time (0.38 s, SD = 0.13) than the advertisement call (mean duration 0.35 s, SD = 0.05; rise time 0.18 s; SD = 0.01), but with less pulses/call (mean 22.11, SD = 5.71; mean 30.39 s, SD = 2.75 for the advisement call). In most calls, a multiple pulse introductory note is present at the beginning of the call (mean duration 0.26 s, SD = 0.07) and the first two pulses of the Type II notes are more separated (mean 0.0076 s, SD = 0.0006) from the following pulses (mean 0.0029 s, SD = 0.0006). The average dominant frequency and the frequency bandwidth are 2832 Hz (SD = 106.24) and 735.46 Hz (SD = 120.51). Other call parameters are listed in Tables 5 and 6.

**Distribution and ecology**. *Dendropsophus reticulatus* occurs in the Amazon basin of Brazil, Colombia, Ecuador, Peru and Bolivia (Fig 2). The descriptions and pictures of *Hyla laynei* published by Goin [53] confirms its presence in Colombia. Localities with known elevation range from 40 to 1037 m above sea level. The elevation at Reserva Ecológica Río Anzu (1037 m) is the highest known locality for *D*. *reticulatus*, while Tabatinga (63 m) is the lowest.

*Dendropsophus reticulatus* occurs in *Terra Firme* forest, flooded forests and swamps. It is generally found next to streams, lakes and temporary ponds in clearings. Individuals have been recorded at night perching on vegetation 20 to 100 cm above the ground. Their occurrence in secondary forests and artificial open areas suggest at least some tolerance of anthropogenic habitat disturbance.

Vegetation types for Ecuadorian localities are: (1) Amazonian Lowland Evergreen Forest, characterized by high plant α-diversity and a canopy height of 30 m with emergent trees that reach 40 m; (2) Floodplain Lowland Forest of White-Waters, characterized by periodical flooding with white-waters from large rivers, the vegetation reaching 35 m in height, and having several horizontal strata of vegetation; (3) Lowland Forest of Palms and black-waters, swamps characterized by a canopy approximately 30 m high with dense understory and a dominance of the palm *Mauritia flexuosa*; (4) Amazonian Evergreen Foothill Forest, characterized by a mixture of Amazonian and Andean vegetation with a canopy of 30 m; and (5) Evergreen Lower Montane Forest of the East of the Northern and Central Andes, characterized by a canopy height of 25 to 30 m, with abundant epiphytes, and by the absence of species of trees characteristic of the lowlands (e.g. species from the family *Bombacaceae* and *Myristicaceae*).

Vegetation types at known localities from Peru and Brazil include Napo Moist Forest, Ucayali Moist Forests, Iquitos *Varzea*, and Southwest Amazon Moist Forest (according to the World Wildlife Fund [57]).

**Conservation status**. The distribution polygon of *Dendropsophus reticulatus* has 390,082 km^2^ of which 11,093 km^2^ (2.8%) have been degraded by human activities. Because its geographic range is relatively large and has a low proportion of degraded habitat, we assign *D*. *triangulum* to the Red List category Least Concern.

**Remarks**. Individuals from Nacebe in Bolivia were reported as “*Dendropsophus triangulum*” by Moravec and Aparicio [58]; however the collected specimens according to the description resemble *D*. *reticulatus* comb. nov. (dorsal surfaces cream to yellow with the presence or absence of a small spot in interorbital position).

#### *Dendropsophus arndti* sp. n.

urn:lsid:zoobank.org:act:D85489E7-046C-4EDC-862C-9055291EC88A

*Hyla leucophyllata*—De la Riva et al. [59], Márquez et al. [60–61], Moravec and Aparicio [62]

*Dendropsophus leucophyllatus*—Jansen and Schulze [63], Jansen et al. [64], Schulze et al. [65]

*Dendropsophus leucophyllatus* A—Jansen et al. [4], Schulze et al. [66]

**Common name**. Proposed standard English name: Arndts’ treefrog. Proposed standard Spanish name: ranita de Arndt.

**Holotype** (Figs 11K and 12) SMF 88389 (field no. MJ-1246), adult male from Bolivia, Department Santa Cruz, Province Ñuflo de Chávez, San Sebastián, Centro de Investigaciónes Ecológicas Chiquitos (16.3596° S, 62.0000° W), 529 m above sea level, collected by Martin Jansen on 11 January 2010.

**Paratopotypes** (Fig 12) field no. MJ 27, SMF 88383–84, 88387–88, adult males, 88386, adult female, collected by Martin Jansen on 29 November 2004 and 13 February 2010.

**Paratypes** (Fig 12) Bolivia: Department Beni, Province Yacuma, Los Lagos (12.7719° S, 65.8100° W), 148 m, field no. MJ 729, 820, SMF 88385, 88390, adult males, collected by M. Jansen on 9 January 2007 and 7 February 2010; Department Santa Cruz, Province Ichilo, Buenavista (17.4816° S, 63.6848° W), 333 m, SMF 88391–93, adult males, collected by M. Jansen on 9 March 2012. Specimens with only field no. MJ are stored at Museo de Historia Natural Noel Kempff Mercado (NKM) in Santa Cruz de la Sierra, Bolivia.

**Diagnosis**. A member of the genus *Dendropsophus* characterized by: (1) mean SVL 30 mm in males (range 28–32.4; *n* = 13); 33.2 mm in females (*n* = 1); (2) axillary membrane reaching arm halfway to elbow; (3) basal webbing on fingers; (4) webbing on feet; (5) palmar tubercle single; (6) pectoral patches moderate; (7) background dorsal coloration varying from brown to dark brown with white or bright yellow (in life) irregular dorsolateral bands extending to the head, white or bright yellow (in life) circular sacral mark; (8) light irregular rounded spots on the dorsal surfaces of the limbs (one to two on the forearm and one to three on the shank); (9) some individuals have a reticulated color pattern (Fig 12); (10) in life, webbing and ventral surfaces vary from orange to orange-yellow or pink; (11) in life, iris dull bronze to coppery bronze.

**Description of holotype**. Adult male, SVL 26.7 mm, foot length 12.6 mm, head length 9.2 mm, head width 9.3 mm, eye diameter 3.3 mm, tympanum diameter 2.3 mm, tibia length 14.1 mm, femur length 13 mm, arm length 6 mm, eye-nostril distance 3 mm: body about as wide as head, head broader than long; snout short and rounded in dorsal view, truncate in profile; distance from nostril to eye shorter than diameter of eye; canthus rostralis scarcely distinct, rounded; loreal region plain; internarial region subtly depressed; nostrils slightly protuberant, directed posterolaterally; interorbital area flat; eye large, protuberant; diameter of eye 1.4 times diameter of tympanic annulus; tympanum concealed beneath skin; tympanic annulus visible below skin, ovoid, longer dorsoventrally and concealed dorsally by supratympanic fold, separated from eye by ca. 76% of its diameter; faint supratympanic fold, extending posteriorly from posterior corner of eye to anterior border of arm insertion. Arm slender, axillary membrane reaching arm halfway to elbow; relative length of fingers I < II < IV < III; fingers bearing large oval discs, that of third finger about three fourths of tympanum diameter; subarticular tubercles prominent, round to ovoid, single; distal tubercle on finger IV bifid; supernumerary tubercles present; palmar tubercle indistinct; prepollical tubercle large, flat, elliptical; prepollex elliptical, enlarged; nuptial excrescences absent; webbing formula of fingers I basal II1^1^/_2_−2^1^/_2_III2^2^/_3_—2IV. Hindlimbs moderately long; toes bearing discs slightly wider than long, smaller than those of fingers; relative length of toes I < II < V < III < IV; outer metatarsal tubercle poorly defined, small, round; inner metatarsal tubercle large, elongated and elliptical; subarticular tubercles single, round, flat; supernumerary tubercles restricted to the soles; webbing formula of toes I1^+^—2II1^+^—2^-^III1^+^—2IV2^+^—1^+^V. Two glandular patches on the chest posterior to the clavicle, separated from each other by about half their width. Skin on dorsum, head, and dorsal surfaces of limbs smooth; skin on flanks smooth with weak longitudinal wrinkles posterior to arm; skin on venter coarsely granular except for the pectoral patches which are finely granular; skin on ventral surfaces of head and thighs granular, except for the vocal sac in the gular area to anterior edge of clavicle, which has longitudinal wrinkles; skin of shanks smooth. Cloacal opening at the level of upper edges of thighs; short, simple cloacal sheath covering cloacal opening. Tongue broadly cordiform, free laterally and posteriorly, widely attached to mouth floor; vomerine odontophores situated between choanae, in two slightly angled series, not in contact with each other, about as wide as choanae, each bearing 3–4 vomerine teeth; choanae ovoid.

*Color of holotype in preservative* (Fig 12). Dorsum brown with white dorsolateral bands extending to the head with irregular edges and scattered small brown spots, white round mark on sacrum with the same pattern as dorsolateral bands; tip of the snout, sides of the head, supra-cloacal region, flanks, and dorsal surfaces of limbs brown; two irregular white rounded spots with irregular edges and small brown spots on the dorsal surfaces of forearms, three similar spots on shanks; venter creamy white; ventral surfaces of limbs and webbing yellowish white; pectoral patches cream.

*Color of holotype in life* (Fig 11K). Dorsum background color was dark brown. White rounded spots and marks in the preserved specimen were bright yellow with scattered small dark brown spots in life; tip of the nose, sides of the head, supra-cloacal region, flanks, and dorsal surfaces of the limbs were brown; scattered, minute yellow spots were present on the limbs, flanks, and sides of the head; ventral surfaces of limbs and webbing were pink; these surfaces are orange by day; iris dull bronze.

**Variation**. Coloration in this section refers to preserved specimens unless otherwise stated. Variation in dorsal and ventral coloration of preserved specimens is shown in Fig 12. Background dorsal coloration varies from brown (e.g., SMF 88390, 88392) to dark brown (e.g., SMF 88383, 88386). In most specimens, the clear dorsolateral bands and rounded sacral mark have irregular edges and are spotted with brown (e.g., SMF 88387, 88384) while in others specimens the dorsolateral bands are smooth without spots (e.g., SMF 88388, 88390). The tip of the snout, sides of the head, supra-cloacal region, flanks, and dorsal surfaces of the limbs are brown to dark brown (e.g., SMF 88384, 88386). Irregular white rounded spots (sometimes with small brown spots) are present on the dorsal surfaces of the forearms (one to two for each one) and shanks (one or three on each one) (e.g., SMF 88383, 88387, 88392). In some individuals, similar but smaller stains are present on the feet and hands (one or two for each one) (e.g., SMF 88387–88). The thighs are immaculate with or without a thin brown stripe along the dorsal surface. Some specimens have the reticulated dorsal pattern, which consists of a fine network of white lines on the dorsal region, sides of the head and body with a dark brown to brown background (e.g., SMF 88391). However, despite years of intensive fieldwork at the type locality, reticulated dorsal pattern was never observed in such population. The interspaces are as large or larger than the eye, excepting on the sides of the head and body, where they are smaller. Ventral areas, webbing and discs vary from creamy white (e.g., SMF 88391, 88393) to yellowish white (e.g., SMF 88383, 88388) with cream pectoral patches.

**Coloration in life** (based on digital photographs; Fig 11K–11M). Dorsum and dorsal surfaces of the limbs vary from brown to dark brown, sometimes spotted with white (e.g., SMF 88386, 88388). White dorsolateral bands, rounded spots and sacral marks in preserved specimens are white or bright yellow in life (e.g., SMF 88392). In most specimens, these bands, spots and marks have scattered brown spots (e.g., SMF 88384, 88386). The tip of the snout, sides of the head, supra-cloacal region, flanks and dorsal surfaces of the limbs are brown to dark brown (e.g., SMF 88386). Individuals with the reticulate color pattern have a dark brown to brown background with white or bright yellow network lines (e.g., SMF 88391). At night the ventral surfaces of the limbs, anterior, and posterior surfaces of thighs and webbing are orange-yellow (e.g., SMF 88391) to pink; these surfaces change to orange by day (e.g., SMF 88384). The vocal sac is yellow, the belly is creamy white and the iris is dull bronze or coppery bronze.

**Comparisons with other species**. *Dendropsophus arndti* is most similar to *D*. *leucophyllatus* and *D*. *triangulum*. It differs from both species by having a rounded sacral mark with irregular edges and small dark spots (edges are smooth and dark spots are absent in *D*. *leucophyllatus* and *D*. *triangulum*; this mark is leaf-shaped in *D*. *leucophyllatus*). *Dendropsophus arndti* further differs from *D*. *leucophyllatus* and *D*. *triangulum* in advisement calls (lower duration and number of pulses of the Type I note in *D*. *leucophyllatus*; lower frequency bandwidth and dominant frequency in *D*. *triangulum*)

*Dendropsophus arndti* differs from *D*. *reticulatus* in advertisement call (Fig 4E vs. 4G) and by its larger size (male SVL range 28–32.4 mm vs. 20–29.6 mm in *D*. *reticulatus*; differences are significant: *t* = -14.04, df = 15, *P* < 0.001; Table 3); specimens of *D*. *reticulatus* have a more uniform dorsal color, sometimes punctuated by one or several brown marks (Fig 9); furthermore, ventral surfaces are red in *D*. *reticulatus* (orange in *D*. *arndti*). *Dendropsophus reticulatus* with the reticulated pattern differ from those of *D*. *arndti*, in having dark interspaces smaller than the eye (larger than the eye in *D*. *arndti*; compared CORBIDI 12253, Fig 9 vs. SMF 88391, Fig 12).

*Dendropsophus arndti* is larger (non-overlapping ranges in SVL) than *D*. *bifurcus* (male SVL range 28–32.4 mm vs. 23–28 mm in *D*. *bifurcus* [21]), *D*. *manonegra* (22.7–25.1 mm [8]), *D*. *rossalleni* (19–22.3 mm [56]), and *D*. *vraemi* sp. n. (25.1–27.6 mm). It can be distinguished from *D*. *manonegra*, *D*. *rossalleni*, *D*. *sarayacuensis*, and *D*. *vraemi* by having dorsolateral wide bands (dorsolateral bands are thin or absent in the four species). *Dendropsophus rossalleni* has pale creamy white spots on the upper eyelids and adjacent areas of the head [56] (pale mark on the head extends to the snout, the scapular marks are larger and more diagonal, and always depart from behind the orbits in *D*. *arndti*, Fig 12); *D*. *sarayacuensis* has dorsolateral bands that only extend to the midbody (bands extend to the sacral area in *D*. *arndti*) and frayed rounded spots on the dorsal surfaces of the limbs (well defined spots in *D*. *arndti*). *Dendropsophus arndti* further differs from *D*. *bifurcus* and the holotype of *D*. *salli* (MNK-A 8445; see Discussion section) in the shape of the clear mark on the sacrum (rounded with irregular edges and scattered small spots in *D*. *arndti*; rounded well defined and without spots in *D*. *bifurcus*; medial rhomboidal mark in *D*. *salli* [10]).

**Calls** (Fig 4G; Table 5). The advertisement call of *Dendropsophus arndti* consists of one pulsed trill note (Type I) with a mean duration of 0.19 s (SD = 0.02; range = 0.16–0.23 s; *n* = 7). The mean dominant frequency is 2655.4 Hz (SD = 169.42). The mean rise time is 0.12 (SD = 0.08) and has a frequency bandwidth of 487.67 Hz (SD = 75.06). Type I note has 15–19 pulses/note (mean 17.44, SD = 1.23). Type II notes are absent.

The aggressive call (Fig 4H; Table 6) consists of 3–4 Type II pulsed notes with shorter duration (mean 0.035 s, SD = 0.006) and fewer pulses (3–4 pulses/note) than the Type I note. The aggressive call has a mean dominant frequency of 2668.76 Hz (SD = 111.64), mean frequency bandwidth of 423.26 Hz (SD = 64.73), mean rise time of 0.07 s (SD = 0.02), and mean distance between notes of 0.020 s (SD = 0.011). Mean duration is 0.14 s (SD = 0.01). The aggressive call has fewer pulses (12–13) than the advertisement call (15–19). Other call parameters are listed in Tables 5 and 6.

**Tadpole**. For a detailed description of the tadpole (as *Dendropsophus leucophyllatus* A) see Schulze et al. [66].

**Distribution and ecology**. *Dendropsophus arndti* has been recorded in the Bolivian Amazon Basin (Beni and Santa Cruz departments) (Fig 2). Localities with known elevation range from 148 to 529 m above sea level. The elevation at San Sebastián (around 530 m) is the highest known locality while Los Lagos (around 150 m) is the lowest. Vegetation types at known localities are Beni Savanna, Chiquitano Dry Forests, and Bolivian *Yungas* (according to the World Wildlife Fund [57]).

Specimens of *Dendropsophus arndti* were found at night along river shores, edges of the forest, temporary swamplands, and artificial ponds, perching on vegetation from 0 to 140 cm above the ground or water surface [65]. Jansen and Schulze [63] observed a spider (*Ancylometes* sp.) preying on *D*. *arndti*.

**Conservation status**. The distribution polygon has an area of 66,397 km^2^ of which 3,834 km^2^ (5.8%) have been degraded by human activities. Because its distribution range is relatively large and has a low proportion of degraded habitat, we suggest assigning *D*. *arndti* to the Red List category Least Concern.

**Etymology**. The specific name is a patronym for Professor Emeritus Dr. Rudolf G. Arndt (Pomona, Galloway Township, New Jersey, USA) in recognition of his financial support for scientific research by M. Jansen and for nature conservation.

#### *Dendropsophus vraemi* sp. n.

urn:lsid:zoobank.org:act:A2E24960-FE61-49AA-8E1E-AA157A223C14

**Common name**. Proposed standard English name: Vraem’ treefrog. Proposed standard Spanish name: ranita del Vraem.

**Holotype**. (Figs 11N and 11O and 13) CORBIDI 9614 (field no. VD-013), adult male from Peru, Region of Ayacucho, La Mar, KP180, stream of the Apurímac river (12.9296° S, 73.5330° W), 694 m above sea level, collected by Germán Chávez and Vilma Durán on 23 July 2011.

**Paratopotypes**. (Fig 13) CORBIDI 9613, 9640–44, 9646, adult males, collected with the holotype.

**Diagnosis**. A member of the genus *Dendropsophus* characterized by: (1) mean SVL 26 mm in males (range 25.1–27.6; *n* = 8); (2) axillary membrane reaching arm halfway to elbow; (3) basal webbing on fingers; (4) webbing on feet; (5) palmar tubercle bifid; (6) pectoral patches moderate; (7) background dorsal coloration varying from brown to grayish brown with creamy white or light brown thin dorsolateral bands extending to the head; (8) rounded marks on the dorsal surfaces of the shanks and lateral stripes on the sacrum; (9) reticulated dorsal pattern absent; (10) in life, ventral surfaces yellowish with limbs and webbing orange or pink; (11) in life, iris gray bronze.

**Description of holotype**. Adult male, SVL 26.6 mm, foot length 13.2 mm, head length 7.4 mm, head width 8.6 mm, eye diameter 3.2 mm, tympanum diameter 2.5 mm, tibia length 13.9 mm, femur length 13.4 mm, arm length 5.1 mm, eye-nostril distance 2.1 mm; body about as wide as head, head slightly broader than long; snout short and rounded in dorsal view, truncate in profile; distance from nostril to eye shorter than diameter of eye; canthus rostralis scarcely distinct, rounded; loreal region plain; internarial region subtly depressed; nostrils slightly protuberant, directed posterolaterally; interorbital area flat; eye large, protuberant; diameter of eye 1.25 times diameter of tympanic annulus; tympanum concealed beneath skin; tympanic annulus visible below skin, ovoid, longer dorsoventrally and concealed dorsally by supratympanic fold, separated from eye by ca. 54% of its diameter; faint supratympanic fold, extending posteriorly from posterior corner of eye to anterior border of arm insertion. Arm slender, axillary membrane reaching arm halfway to the elbow; relative length of fingers I < II < IV < III; fingers bearing large oval discs, that of third finger about three fourths of tympanum diameter; subarticular tubercles prominent, round to ovoid, single; distal tubercle on finger IV bifid; prominent and abundant supernumerary tubercles; palmar tubercle small, bifid; prepollical tubercle large, flat, elliptical; prepollex elliptical, enlarged; nuptial excrescences absent; webbing formula of fingers I basal II1^1^/_2_−2^1^/_2_III2^1^/_2_−2^+^IV. Hindlimbs moderately long; toes bearing discs slightly wider than long, smaller than those of fingers; relative length of toes I < II < V < III < IV; outer metatarsal tubercle conspicuous, small, round; inner metatarsal tubercle large, elongated and elliptical; subarticular tubercles single, round, flat; prominent and abundant supernumerary tubercles restricted to the soles; webbing formula of toes I1^+^—2^-^II1^-^—2III1—2IV2^+^—1V. Two glandular patches visible on the chest posterior to the clavicle, separated from each other by about half their width. Skin on dorsum, head, and dorsal surfaces of limbs smooth; skin on flanks smooth with weak longitudinal wrinkles posterior to arm; skin on venter coarsely granular, the pectoral patches finely granular; skin on ventral surfaces of head and thighs granular, except for the vocal sac in the gular area to anterior edge of clavicle, which is longitudinally wrinkled; skin of shanks smooth. Cloacal opening at level of upper edges of thighs; short, simple cloacal sheath covering cloacal opening. Tongue broadly cordiform, free laterally and posteriorly, widely attached to mouth floor; vomerine odontophores between choanae, in two slightly angled series, but not in contact with each other, about as wide as choanae, with indistinguishable vomerine teeth; choanae ovoid.

*Color of holotype in preservative* (Fig 13). Dorsal surfaces of limbs, sides of head, flanks and dorsum grayish brown; top of head, anterior to the orbits, white cream; clear head coloration continuing posteriorly as creamy white, thin dorsolateral bands that reach the anterior portion of sacrum; two dorsolateral creamy white thin stripes present on the sacrum area; creamy white rounded to ovoid marks on dorsal surfaces of shanks (one on the heel and the other on the middle); ventral areas, webbing and discs creamy white with yellowish pectoral patches.

*Color of holotype in life*. (Fig 11N and 11O). Similar to coloration of the preserved specimen except that ventral surfaces of the head, vocal sac, and belly were yellowish; ventral surfaces of the limbs, webbing and discs orange; iris dull bronze.

**Etymology**. The specific epithet *vraemi* is a noun derived from VRAEM, abbreviated acronym of the Apurimac, Ene and Mantaro River Valleys, where the three rivers converge. All type specimens of *Dendropsophus vraemi* were collected in this zone, near to the San Antonio town in La Mar, Region of Ayacucho.

**Variation**. Coloration in this section refers to preserved specimens unless otherwise stated. Variation in dorsal and ventral coloration of preserved specimens is shown in Fig 13. Background dorsal coloration varies from brown to dark brown (e.g., CORBIDI 9613, 9642) or grayish brown (e.g., CORBIDI 9646) with creamy white (e.g., CORBIDI 9646) or light brown (e.g., CORBIDI 9613, 9642) on the dorsal surface of the head anterior to the orbits; white or light brown thin dorsolateral bands extended from the head to the sacrum (e.g., CORBIDI 9640–42). In most of the specimens, two or four dorsolateral creamy white or light brown unconnected stripes are present on the posterior half of the sacral area (e.g., CORBIDI 9613, 9640, 9643–44). The tip of the nose, sides of the head, supra cloacal region, flanks, and dorsal surfaces of the limbs are dark brown to grayish brown. Two creamy white or light brown rounded spots are present on the dorsal surfaces of the shanks, one on the heel and the other on the middle of the shank (e.g., CORBIDI 9640–43). The dorsal surfaces of the thighs are brown or immaculate with a thin brown stripe along the dorsal surface. Ventral areas, webbing and discs are creamy white pectoral patches yellowish (e.g., CORBIDI 9613, 9641).

**Comparisons with other species**. *Dendropsophus vraemi* is most similar to *D*. *bifurcus* and the holotype of *D*. *salli* (MNK-A 8445; see Discussion section), but it can be distinguished by the shape and number of the marks on the sacrum (two to four stripes in *D*. *vraemi* vs. one rounded or rhomboidal mark in *D*. *bifurcus* and *D*. *salli* [10]). *Dendropsophus reticulatus* can be distinguished from *D*. *vraemi* by having, in life, an orange venter (yellowish in *D*. *vraemi*). They also differ in dorsal coloration as *D*. *vraemi* has stripes on the sacrum with clear thin dorsolateral bands (absent in *D*. *reticulatus*; Figs 9 vs. 13).

*Dendropsophus vraemi* adult males (25.1–27.6 mm SVL) are smaller (non-overlapping ranges) than adult males of *D*. *arndti* (28–32.4 mm) and *D*. *triangulum* (28.6–34.4 mm) and it can be distinguished by having thin dorsolateral bands (dorsolateral bands are wide in both species and also in *D*. *leucophyllatus*; Figs 13 vs. 6, 8 and 12). *Dendropsophus rossalleni* can be distinguished of *D*. *vraemi* in having a dull cream venter (yellowish in *D*. *vraemi)* and pale creamy white spots on the anteromedial parts of the upper eyelids and adjacent part of the head [56] (a pale triangular mark on the tip of the head with its base in the interorbital area and its apex between the nostrils in *D*. *vraemi*; Fig 13); *D*. *manonegra* has, in life, bluish-black coloration on digits, webbing, axillary membranes, groin and hidden surfaces of arms and legs [8] (orange or pink in *D*. *vraemi*); *D*. *sarayacuensis* has frayed dorsolateral bands and rounded spots with irregular borders on the dorsal surfaces of the limbs (smooth and well defined in *D*. *vraemi*).

**Distribution and ecology**. *Dendropsophus vraemi* is known from a single locality, La Mar, in the Peruvian Amazon basin, elevation of 694 m (Region of Ayacucho) (Fig 2). Individuals were collected at night perching on pastures 50 cm above the ground in a small stream, which flows into the Apurímac river, near to the San Antonio town. Vegetation type at the type locality is Peruvian *Yungas* (according to the World Wildlife Fund [57]).

**Conservation status**. Given the limited available information on *D*. *vraemi*, and following IUCN (International Union for Conservation of Nature) (2001) criteria, we suggest placing this species in the Data Deficient category.

## Discussion

We assessed the systematics of *Dendropsophus leucophyllatus*, *D*. *triangulum* and related species based on a molecular phylogeny, morphology, and call characters. Our phylogeny is consistent with previous analyses [8,10,11,15–19] that included fewer species of the *Dendropsophus leucophyllatus* group.

We found qualitative and quantitative morphological differences between some clades (A to C and I) of the *Dendropsophus leucophyllatus-triangulum* complex. In contrast, frogs from clades D, E, F, G and H have similar morphology despite deep mtDNA differentiation (p-genetic distance range 2.5–6.8% for gene 16S). Our analyses of advertisement calls show differences between some of these clades (D and H vs. F and G), but in some cases calls are very similar (F vs. G). We also found that some of these clades are distributed sympatrically or at least in very close proximity (e.g., clades D, E and H are present at La Convención; D and E at Tambopata; D and G at Redenção). Phenotypically and genetically differentiated groups that maintain their genetic integrity in sympatry are probably reproductively isolated and can be considered as different species [67]. Nevertheless, phenotypically similar populations with divergent mtDNA lineages (such as clades D to H) could arise from secondary contact between populations that were previously separated but could not reach reproductive isolation [68]. The nuDNA results show low differentiation and species paraphyly, a common outcome when few loci are considered.

Given that clades D–G + H have similar morphologies they could be considered as Deep Conspecific Lineages (DCL). However, because we have few individuals and localities, we prefer to consider clades D–G as Unconfirmed Candidate Species (UCS) until more data become available. Further studies should include advertisement calls from localities where those UCS occur in sympatry. Although clade H was assigned to *Dendropsophus triangulum* due to its morphology and proximity to the type locality, it is possible that the binomen also applies to clades D, E, F and G.

We found high genetic distances among populations of *D*. *sarayacuensis* and *D*. *salli* (6% and 3%, respectively for gene 16S). These distances are lower than those reported between described species within the *D*. *leucophyllatus* and *D*. *parviceps* species groups ([69], but see [8] for one exception). This suggest that the differentiation within *D*. *sarayacuensis*, *D*. *salli* and between clades D–H is lower than typical interspecific distances in *Dendropsophus*. Nevertheless, detailed taxonomic reviews of each species are needed to reject conclusively the existence of cryptic species. This is especially necessary in *D*. *salli*, a species with extensive morphological variation in the type material [10]. Unfortunately, the holotype was not sequenced but the shape of its sacral mark resembles individuals of *D*. *leucophyllatus*. The paratypes have a color pattern distinct from the holotype and their sequences are sister to *D*. *vraemi* [10]. If the holotype is a different species from the paratypes, “*D*. *salli*” in our phylogeny could represent one or two undescribed species.

The discovery of additional new species in the *D*. *leucophyllatus-triangulum* complex is to be expected, especially in Colombia and Brazil, where further taxonomic work and molecular analysis are needed. This study, like similar others, highlights the importance of integrative approaches and international collaborations to clarify the status of taxonomically difficult species groups of the Neotropical frogs.

## Supporting information

S1 Appendix Additional specimens examined. (DOCX)

S1 Fig Adult preserved specimens of *Dendropsophus* sp. D and E showing variation in dorsal and ventral coloration. (TIF)

S1 Table Accession names of the sound archives used in the bioacoustics analysis. (DOCX)

S2 Table Primers used for DNA amplification. (DOCX)

S3 Table Genbank accession numbers for DNA sequences used in the phylogenetic analysis. (DOCX)

## References

[1] Brook BW, Bradshaw CJ, Lian LP, Navjot SS. Momentum drives the crash: mass extinction in the tropics. Biotropica. 2006; 38: 302–305.

[2] Jenkins CN, Pimm SL, Joppa LN. Global patterns of terrestrial vertebrate diversity and conservation. Proceedings of the National Academy of Sciences of the United States of America. 2013; 110: 2602–2610. 10.1073/pnas.1302251110 PMC3710798 23803854

[3] Fouquet A, Gilles A, Vences M, Marty C, Blanc M, Gemmell NJ. Underestimation of species richness in neotropical frogs revealed by mtDNA analyses. PLoS One. 2007; e1109 10.1371/journal.pone.0001109 17971872 PMC2040503

[4] Jansen M, Bloch R, Schulze A, Pfenninger M. Integrative inventory of Bolivia’s lowland anurans reveals hidden diversity. Zoologica Scripta. 2011; 40: 567–583.

[5] Gehara M, Crawford AJ, Orrico VGD, Rodríguez A, Lötters S, Fouquet A, et al High levels of diversity uncovered in a widespread nominal taxon: Continental phylogeography of the neotropical tree frog *Dendropsophus minutus*. PLoS One. 2014; 9 (9) e103958 10.1371/journal.pone.0103958 25208078 PMC4160190

[6] Frost, Darrel R. Amphibian Species of the World: an Online Reference. Version 6.0 (Date of access). Electronic Database accessible at http://research.amnh.org/herpetology/amphibia/index.html. American Museum of Natural History, New York, USA. 2016.

[7] Faivovich J, Haddad CFB, García PCA, Frost DR, Campbell JA, Wheeler WC. Systematic review of the frog family Hylidae, with special reference to Hylinae: phylogenetic analysis and taxonomic revision. Bulletin of the American Museum of Natural History. 2005; 294: 1–240.

[8] Rivera-Correa M, Orrico VGD. Description and phylogenetic relationships of a new species of treefrog of the *Dendropsophus leucophyllatus* group (Anura: Hylidae) from the Amazon basin of Colombia and with an exceptional color pattern. Zootaxa. 2013; 3686: 447–460. 26473232 10.11646/zootaxa.3686.4.3

[9] Duellman WE. A reassessment of the taxonomic status of some Neotropical hylid frogs. Occasional Papers of the Museum of Natural History University of Kansas. 1974; 27: 1–27.

[10] Jungfer KH, Reichle S, Piskurek O. Description of a new cryptic southwestern Amazonian species of leaf-gluing treefrog, genus *Dendropsophus* (Amphibia: Anura: Hylidae). Salamandra. 2010; 46: 204–213.

[11] Chek AA, Lougheed SC, Bogart JP, Boag PT. Perception and history: molecular phylogeny of a diverse group of neotropical frogs, the 30-chromosome *Hyla* (Anura: Hylidae). Molecular Phylogenetics and Evolution. 2001; 18: 370–85. 10.1006/mpev.2000.0889 11277631

[12] Salducci MD, Marty C, Chappaz R, Gilles A. Molecular phylogeny of French Guiana hylinae: implications for the systematic and biodiversity of the neotropical frogs. Comptes Rendus Biologies. 2002; 325: 141–153. 10.1016/S1631-0691(02)01423-3 11980175

[13] Salducci MD, Marty C, Fouquet A, Gilles A. Phylogenetic relationships and biodiversity in hylids (Anura: Hylidae) from French Guiana. Comptes Rendus Biologies. 2005; 328: 1009–1024. 10.1016/j.crvi.2005.07.005 16286090

[14] Fouquet A, Noonan BP, Blanc M, Orrico VGD. Phylogenetic position of *Dendropsophus gaucheri* (Lescure and Marty 2000) highlights the need for an in-depth investigation of the phylogenetic relationships of *Dendropsophus* (Anura: Hylidae). Zootaxa. 2011; 3035: 59–67.

[15] Wiens JJ, Kuczynski CA, Hua X, Moen DS. An expanded phylogeny of treefrogs (Hylidae) based on nuclear and mitochondrial sequence data. Molecular Phylogenetics and Evolution. 2010; 55: 871–882. 10.1016/j.ympev.2010.03.013 20304077

[16] Pyron RA, Wiens JJ. A large-scale phylogeny of Amphibia including over 2800 species, and a revised classification of extant frogs, salamanders, and caecilians. Molecular Phylogenetics and Evolution. 2011; 61: 543–583. 10.1016/j.ympev.2011.06.012 21723399

[17] Motta AP, Castroviejo-Fisher S, Venegas PJ, Orrico VGD, Padial JM. A new species of the *Dendropsophus parviceps* group from the western Amazon Basin (Amphibia: Anura: Hylidae). Zootaxa. 2012; 3249: 18–30.

[18] Ferreira RB, Faivovich J, Beard KH, Pombal JP Jr. The First Bromeligenous Species of *Dendropsophus* (Anura: Hylidae) from Brazil's Atlantic Forest. PLoS One. 2015; 10 (12): e0142893 10.1371/journal.pone.0142893 26650515 PMC4674083

[19] Lougheed SC, Austin JD, Bogart JP, Boag PT, Chek AA. Multicharacter perspectives on the evolution of intraspecific differentiation in a Neotropical hylid frog. BMC Evolutionary Biology. 2006; 6: 1–16. 16539709 10.1186/1471-2148-6-23 PMC1434785

[20] Duellman WE. The biology of an Equatorial herpetofauna in Amazonian Ecuador. University of Kansas Museum of Natural History. Miscellaneous Publication. 1978; 65: 1–352.

[21] Rodríguez LO, Duellman WE. Guide to the frogs of the Iquitos region, Amazonian Perú (Vol. 22). Lawrence, Kansas. Asociación de Ecología y Conservación, Amazon Center for Environmental Education and Research, and Natural History Museum, The University of Kansas. 1994.

[22] Duellman WE. Cusco Amazónico, the lives of amphibian and reptiles in an Amazonian rainforest. Cornell University Press, Ithaca 2005.

[23] Ron SR, Guayasamin JM, Yánez-Muñoz MH, Merino-Viteri A, Ortiz DA, Nicolalde DA. AmphibiaWebEcuador. Version 2016.0. http://zoologia.puce.edu.ec/Vertebrados/anfibios/AnfibiosEcuador. 2016.

[24] Titus TA, Hillis DM, Duellman WE. Color polymorphism in neotropical treefrogs: An allozymic investigation of the taxonomic status of *Hyla favosa* Cope. Herpetologica. 1989; 45: 17–23.

[25] McDiarmid R. Measuring and monitoring biological diversity Standard methods for amphibians: Preparing amphibians as scientific specimens. In: Heyer R, Donnelly M, McDiarmid R, Hayek L, Foster M, editors. Washington-London: Smithsonian Books; 1994.

[26] Duellman WE. Hylid frogs of Middle America. Monograph of the Museum of Natural History University of Kansas. 1970; 1: 1–753.

[27] Duellman WE, Pyles RA. Acoustic resource partitioning in anuran communities. Copeia. 1983; 639–649.

[28] Charif RA, Strickman LM, Waack AM. Raven Pro 1.4 User's Manual. The Cornell Lab of Ornithology, Ithaca, NY, USA 2010.

[29] Toledo LF, Martins IA, Bruschi DP, Passos MA, Alexandre C, Haddad CFB. The anuran calling repertoire in the light of social context. Acta Ethologica. 2014.

[30] SAS Institute. User guide. SAS Institute Cary Version 9.01 http://www.jmp.com/. 2010.

[31] Blair WF. Mating call in the speciation of anuran amphibians. The American Naturalist. 1958; 92: 27–51.

[32] ESRI. ArcMap 10.0. Environmental System Research Institute, In; 2010.

[33] Sierra R, Cerón C, Palacios W, Valencia R. Mapa de vegetación del Ecuador Continental 1:1’000.000. Proyecto INEFAN/GEF-BIRF, Wildlife Conservation Society and Ecociencia, Quito. 1999.

[34] Sambrook J, Fritsch EF, Maniatis T. Molecular Cloning: a Laboratory Manual. Cold Spring Harbor Laboratory Press, New York, USA 1989.

[35] Maddison WP, Maddison DR. Mesquite: a modular system for evolutionary analysis. Version 3.04. http://mesquiteproject.org/. 2015.

[36] Lanfear R, Calcott B, Ho SYW, Guindon S. PartitionFinder: combined selection of partitioning schemes and substitution models for phylogenetic analyses. Molecular Biology and Evolution. 2012; 29: 1695–1701. 10.1093/molbev/mss020 10.1093/molbev/mss020 22319168

[37] Rambaut A, Drummond A. TRACER. MCMC trace analysis tool, Version 1.4 University of Oxford http://tree.bio.ed.ac.uk/software/tracer. 2007.

[38] Ronquist F, Teslenko M, van der Mark P, Ayres DL, Darling A, Höhna S, et al MrBayes 3.2: Bayesian phylogenetic efficient inference and model choice across a large model space. Systematic Biology. 2012; 61: 539–542. 10.1093/sysbio/sys029 22357727 PMC3329765

[39] Zwickl DJ. Genetic algorithm approaches for the phylogenetic analysis of large biological sequence datasets under the maximum likelihood criterion. Ph.D. dissertation, University of Texas, Austin, 125 pp. 2006.

[40] Tamura K, Peterson D, Peterson N, Stecher G, Nei M, Kumar S. MEGA5: Molecular Evolutionary Genetics Analysis using Maximum Likelihood, Evolutionary Distance, and Maximum Parsimony Methods. Molecular Biology and Evolution. 2011; 28: 2731–2739. 10.1093/molbev/msr121 21546353 PMC3203626

[41] Vieites DR, Wollenberg KC, Andreone F, Köhler J, Glaw F, Vences M. Vast underestimation of Madagascar’s biodiversity evidenced by an integrative amphibian inventory. Proceedings of the National Academy of Sciences of the United States of America. 2009; 106: 8267–8272. 10.1073/pnas.0810821106 19416818 PMC2688882

[42] Coloma LA, Endara-Carvajal S, Dueñas JF, Paredes-Recalde A, Morales-Mite M, Almeida-Reinoso D, et al Molecular phylogenetics of stream treefrogs of the *Hyloscirtus larinopygion* group (Anura: Hylidae), and description of two new species from Ecuador. Zootaxa. 2012; 3364: 1–78.

[43] Caminer MA, Ron SR. Systematics of treefrogs of the *Hypsiboas calcaratus* and *Hypsiboas fasciatus* species complex (Anura, Hylidae) with the description of four new species. ZooKeys. 2014; 370, 1 10.3897/zookeys.370.6291 PMC3904076 24478591

[44] ICZN [International Commission on Zoological Nomenclature]. Amendment of Articles 8, 9, 10, 21 and 78 of the International Code of Zoological Nomenclautre to expand and refine methods of publication. Zootoaxa. 2012; 3450: 1–7. 10.3897/zookeys.219.3994 PMC3433695 22977348

[45] Böhme W. Zum Problem der Typisierung von *Rana leucophyllata* Bereis, 1783 (Salientia: Hylidae): Recherchen über ehemalige Sammlungen in Lüneburg und Helmstedt. Bonner Zoologische Beiträge. 1981; 32: 283–295.

[46] Beireis GC. Beschreibung eines bisher unbekannt gewesenen amerikanischen Froschen, welcher sich in der Naturaliensammlung des Herrn Hofraths Beireis in Helmstädt befindet. Schriften der Berlinischen Gesellschaft Naturforschender Freunde. 1783; 4: 178–182.

[47] Condit JM. A list of the types of hylid frogs in the collection of the British Museum (Natural History). Journal of the Ohio Herpetological Society. 1964; 4: 85–98.

[48] Günther ACLG. First account of species of tailless batrachians added to the collection of the British Museum. Proceedings of the Zoological Society of London. 1869 "1868"; 478–490.

[49] Darst CR, Cannatella DC. Novel relationships among hyloid frogs inferred from 12S and 16S mitochondrial DNA sequences. Molecular Phylogenetics and Evolution. 2004; 31: 462–475. 10.1016/j.ympev.2003.09.003 15062788

[50] Wiens JJ, Graham CH, Moen DS, Smith SA, Reeder TW. Evolutionary and ecological causes of the latitudinal diversity gradient in hylid frogs: treefrog trees unearth the roots of high tropical diversity. American Naturalist. 2006; 168: 579–596. 10.1086/507882 17080358

[51] Duellman WE. Liste der rezenten Amphibien und Reptilien. Hylidae, Centrolenidae, Pseudidae. Das Tierreich. 1977; 95: 1–225.

[52] Daudin FM. "An. XI". Histoire Naturelle des Rainettes, des Grenouilles et des Crapauds. Quarto version Paris: Levrault 1802.

[53] Goin CJ. Descriptions of two new frogs from Colombia. Journal of the Washington Academy of Sciences. 1957; 47: 60–63.

[54] Cochran DM, Goin CJ. Frogs of Colombia. Bulletin of the United States National Museum. 1970; 288: 1–655.

[55] Frank N, Ramus E. Complete Guide to Scientific and Common Names of Amphibians and Reptiles of the World. Pottsville, Pennsylvania: N. G. Publishing Inc 1995.

[56] De la Riva I, Duellman WE. The identity and distribution of *Hyla rossalleni* Goin. Amphibia-Reptilia. 1997; 18: 433–436.

[57] Fund W. Ecoregion. Retrieved from http://www.eoearth.org/view/article/151948. 2014.

[58] Moravec J, Aparicio J. First record of *Hyla triangulum* Günther, 1868 from Bolivia. Herpetozoa Wien. 2004; 17: 90.

[59] De la Riva I, Köhler J, Lötters S, Reichle S. Ten years of research on Bolivian amphibians: updated checklist, distribution, taxonomic problems, literature and iconography. Revista Española de Herpetología. 2000; 14: 19–164.

[60] Márquez R, De la Riva I, Bosch J. Advertisement calls of Bolivian species of *Hyla* (Amphibia, Anura, Hylidae). Biotropica. 1993; 25: 426–443.

[61] Márquez R, De la Riva I, Bosch J, Matheu E (eds). Guía sonora de las ranas y sapos de Bolivia—Sounds of frogs and toads in Bolivia. Alosa, Fonoteca Zoológica (CD and booklet). 2002.

[62] Moravec J, Aparicio J. Amphibians and reptiles recently recorded from the surroundings of Riberalta (Departamento Beni, Bolivia). Casopis Národního muzea Rada prirodovédná. 2000; 169(1–4): 1–15.

[63] Jansen M, Schulze A. *Dendropsophus leucophyllatus* (Bereis´ Tree Frog). Predation. Herpetological Review. 2008; 39:459.

[64] Jansen M, Schulze A, Werding L, Streit B. Effects of extreme drought in the dry season on an anuran community in the Bolivian Chiquitano region. Salamandra. 2009; 45:233–238.

[65] Schulze A, Jansen M, Köhler G. Diversity and ecology of an anuran community in San Sebastián, Bolivia. Salamandra. 2009; 45: 75–90.

[66] Schulze A, Jansen M, Köhler G. Tadpole diversity of Bolivia`s lowland anuran communities: molecular identification, morphological characterization, and ecological assignment. Zootaxa. 2015; 4016: 1–111. 10.11646/zootaxa.4016.1.1 26624024

[67] Mallet J. Hybridization, ecological races and the nature of species: empirical evidence for the ease of speciation. Philosophical Transactions of the Royal Society B-Biological Sciences. 2008; 363: 2971–2986. 10.1098/rstb.2008.0081 PMC2607318 18579473

[68] Hewitt GM. Quaternary phylogeography: the roots of hybrid zones. Genetica. 2011; 139: 617–638. 10.1007/s10709-011-9547-3 21234647

[69] Fouquet A, Orrico VGD, Ernst R, Blanc M, Martinez Q, Vacher JP, Rodrigues MT, Ouboter P, Jairam R, Ron S. A new *Dendropsophus* Fitzinger, 1843 (Anura: Hylidae) of the parviceps group from the lowlands of the Guiana Shield. Zootaxa. 2015; 4052(1): 39–64. 10.11646/zootaxa.4052.1.2 26624776

