# Correction: Systematics of the *Dendropsophus leucophyllatus* species complex (Anura: Hylidae): Cryptic diversity and the description of two new species

**DOI:** 10.1371/journal.pone.0176902

**Published:** 2017-04-27

**Authors:** Marcel A. Caminer, Borja Milá, Martin Jansen, Antoine Fouquet, Pablo J. Venegas, Germán Chávez, Stephen C. Lougheed, Santiago R. Ron

In Fig 6, the specimen MNHN2015.129 (neotype) appears incorrectly. Please see the correct Fig 6 here.

**Fig 6:**
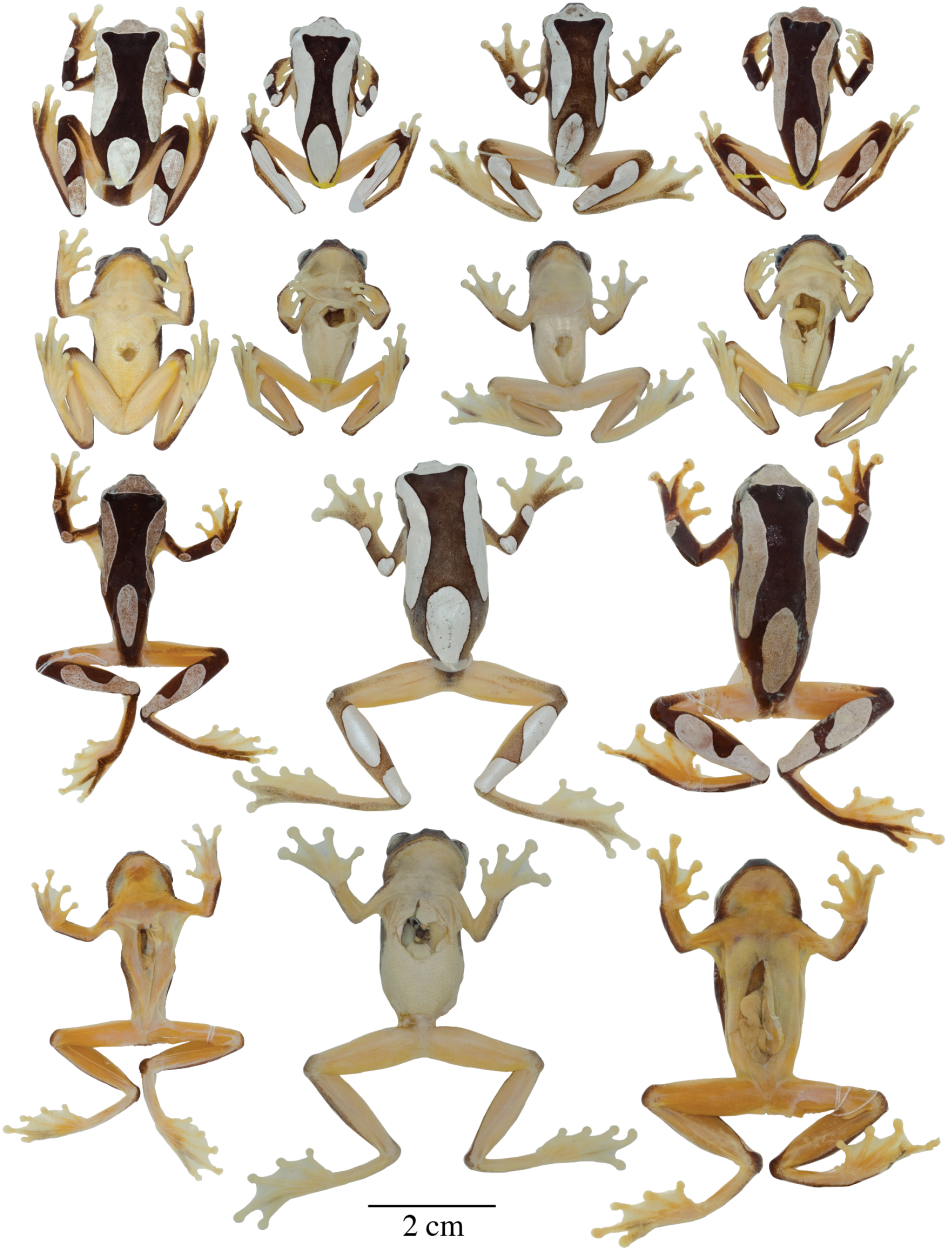
Adult preserved specimens of *Dendropsophus leucophyllatus* showing variation in dorsal and ventral coloration of preserved specimens. From left to right, first and second rows: MNHN2015.129 (neotype), MNHN2015.132, MNHN2015.134, MNHN2015.133 (males); third and fourth rows: MNHN2015.127 (male), MNHN2015.131, MNHN2015.128 (females). See S1 Appendix for locality data. All specimens are shown at the same scale.
