# Supplementary material for: Systematics of the Dendropsophus leucophyllatus species complex (Anura: Hylidae): Cryptic diversity and the description of two new species
Source: PLoS One. 2017 Mar 1;12(3):e0171785. doi: 10.1371/journal.pone.0171785 (PMC5332023; doi:10.1371/journal.pone.0171785)
Supplement: S1 Appendix — (DOCX) [file pone.0171785.s001.docx]

**S1 Appendix. Additional specimens examined.**

*Dendropsophus leucophyllatus.* BRAZIL: STATE AMAPÁ: Serra do Navio (0.9833°N, 52.05°W); 170 m (MZUSP 950143, 950156); STATE AMAZONAS: 100 km north of Manaus (3.1333°S, 60.0166°W), 23 m (MZUSP 950231–32, 950193); STATE PARÁ: Alter do Chão (2.5333°S, 54.9666°W), 73 m (MZUSP 950161); FRENCH GUIANA: Apatou (5.1666°N, 54.3333°W), 17 m (MC 191–92, 373); Aratai (3.9904 °N, 52.5766°W), 78 m (AF 1396–97); Awala (5.7233°N, 53.8894°W), 10 m (AF 1581); Chutes Voltaire, camp Voltaire (5.0522°N, 54.0890°W), 53 m (AF 1748); Inini Tolenga (5.0522°N, 54.0890°W), 118 m (MNHN2015.131); Kaw (4.7166°N, 52.13333°W), 5 m (MC 36, 99, 123–25); Lucifer (4.7666°N, 53.9166°W), 376 m (MC 404); Mana, intersection RN1/route Mana (5.5020°N, 53.5492°W), 37 m (AF 1814–15); Montagne tortue grande (4.2926°N, 52.3495°W), 475 m (AF 1341–44); Petit-saut (5.0666°N, 53.05°W), 15 m (MNHN 2015.127–28); Road to Apura (5.1833°N, 55.65°W), 77 m (BPN 918); Saul (3.6255°N, 53.2072°W), 267 m (MC 224); Saul Limonade, mare crique Nouvelle France (3.5625°N, 53.2104°W), 213 m (AF 1710–14); St Georges RN 2, PK 169 (4.0012°N, 51.9310°W), 78 m (MNHN 2015.132–35); St Laurent (5.4883°N, 54.0119°W), 22 m (AF 1594); Toponowini (3.0527°N, 52.7102°W), 153 m (MC 297, 300); GUYANA: Rupununi, Iwokrama Forest Reserve (USNM 531425–34); SURINAME: PARÁ: Zanderij (USNM 165755).

*Dendropsophus reticulatus.* ECUADOR: PROVINCE NAPO: Río Napo (MNCN 3474; holotype); Isla Anaconda, Río Napo, 25 km downstream from Misahuallí (1.062°S, 77.5366°W), 362 m (KU 202745); Reserva Yachana, Roadside pond off cattle ranch (0.8333°S, 77.1666°W), 300 m (QCAZA 48836, 48840); Comunidad Ita (0.9146°S, 77.7516°W), 703 m (47138, 47140, 47169); Jatun Sacha (1.0649°S, 77.6142°W), 420 (QCAZA 2878); Misahuallí (1.0343°S, 77.6685°W), 401 m (QCAZA 367–69); Reserva Yachana, Roadside pond off cattle ranch (0.8333°S, 77.1666°W), 300 m 48834–35, 48849); Santa Cecilia, 340 m (QCAZA 178); PROVINCE PASTAZA: Around Villano, oil camp of AGIP Villano B-II pool Campament (1.4529°S, 77.4425°W), 391 m (QCAZA 38747–48); Canelos (1.5757°S, 77.7352°W), 650 m (QCAZA 16003, 17411–23, 48701, 48711); Centro Fatima (1.4113°S, 78.00°W), 1023 m (QCAZA 49319, 49338, 49332); Finca km 6 on the road San Ramón-El Triunfo (1.3730°S, 77.86°W), 963 m (QCAZA 37887); Reserva Ecológica Río Anzu (1.4060°S, 78.0478°W), 1037 m (QCAZA 47228); Río Curaray (1.4830°S, 76.6290°W), 235 m (QCAZA 4235); PROVINCE SUCUMBÍOS: 2.5 km N of Lago Agrio (0.1097°S, 76.8746°W), 350 m (KU 217664, QCAZA 2806–10, 2811–20); 800 m East of Jivino Verde (0.1756°S, 76.8261°W), 278 m (QCAZA 47102); Estación Secoya (0.0110°S, 76.5910°W), 600 m (QCAZA 23088–92); Gueppicillo (0.1773°S, 75.6759°W), 220 m (CORBIDI 0137); Lago Agrio (0.10°S, 76.8833°W), 302 m (QCAZA 3158, 6472–73, 15155–15159, 27658); Laguna de Imuya (0.5831°S, 75.2508°W), 177 m (QCAZA 4536); Laguna de Mateocoha (0.0184°S, 76.2215°W), 220 m (QCAZA 26063); Puerto Bolívar (0.0886°S, 76.1420°W), 240 m (QCAZA 28312); Reserva de Producción Faunística Cuyabeno (0.01952°S, 76.2215°W), 225 m (QCAZA 4079); Hostería La Selva, Garza cocha (0.4981°S, 76.3738°W), 250 m (QCAZA 6379, 12008, 12011–12); Hostería La Selva,lado E of Mandi cocha (0.4869°S, 76.3735°W), 250 m (QCAZA 12010); Reserva Limoncocha (0.4079°S, 76.6206°W), 230 m (QCAZA 3625–41, 8015, 8020–23, 8027–28, 24675, 43081, 43084–93, 43116, 43128–30), Shushufindi, Sector Atahualpa (0.0841°S, 76.64°W), 262 m (QCAZA 33961); Tipishca (0.1959°S, 76.2014°W), 229 m (12537); Sacha Lodge, 300 m (QCAZA 4891); PROVINCE ORELLANA: Río Napo, Nuevo Rocafuerte (0.9192°S, 75.4010°W), 187 m (QCAZA 44814–16); Río Napo, Santa Teresita 4 km northwest of New Rocafuerte (0.9008°S, 75.4135°W), 186 m (QCAZA 44668–72); Nuevo Rocafuerte, Alta Florencia (0.8966°S, 75.4370°W), 177 m (QCAZA 39480–81, 39499, 39500); Río Napo, Huiririma (0.7116°S, 75.6239°W), 194 m (QCAZA 44587–96, 44642); Río Napo, San Vicente (0.6790°S, 75.6511°W), 196 m (QCAZA 44485–87); Río Napo, Chiroisla (0.5799°S, 75.9176°W), 207 m (QCAZA 44367–71); Río Napo, Edén (0.4983°S, 76.0711°W), 216 m (QCAZA 44211–20), At about 5 km S of Coca, near the Río Napo (0.4922°S, 77.0032°W), 261 m (QCAZA 43726, 43753, 43758–59, 43760–67); El Coca, on the road El Coca-Loreto E of Río Payanmino (0.4499°S, 77.0210°W), 258 m (QCAZA 43659); El Coca, Francisco de Orellana (0.4691°S, 76.9863°W), 256 m (QCAZA 373–74, 378, 457–61); La Selva (0.4981°S, 76.3738°W), 233 m (QCAZA 7454–55); Parque Nacinal Yasuní Añango (0.5333°S, 76.45°W), 243 m (QCAZA 5051, 5070, 14834–39); on the road El Coca-Loreto near the bridge over the Río Payamino (0.4563°S, 77.3738°W), 268 m (QCAZA 43665–78); Río Napo, la Primavera (0.4442°S, 76.7868°W), 244 m (QCAZA 43818–19); Comunidad Huino (0.6463°S, 77.1474°W), 274 m (QCAZA 30946, 30955–56); 6.2 km E of Loreto (0.72°S, 77.3570°W), 490 m (QCAZA 3079–82, 27650); 18.2 km Southwest of Loreto (0.7495°S, 77.4511°W), 600 m (QCAZA 2746–47, 27656); Huamayacu-Joya de los Sachas (0.3193°S, 76.95°W), 300 m (QCAZA 20308, 20429); Pompeya, Sacha Lodge (0.4705°S, 76.4597°W), 247 m (QCAZA 37872); Bridge over the Río Tiputini near the camp of the ELF (0.7417°S, 76.8611°W), 295 m (QCAZA 7908–09); km 57 on the road to Maxus (0.7554°S, 76.3461°W), 309 m (QCAZA 49199, 49174); Parque Nacional Yasuní, ECY (0.6781°S, 76.3965°W), 250 m (QCAZA 11210); Comuna Indiyama (0.4689°S, 76.5877°W), 243 m (QCAZA 5023); La Joya de los Sachas (0.3227°S, 76.8699°W), 280 m (QCAZA 30653); Comuna El Descanso (0.4310°S, 76.7864°W), 247 m (QCAZA 12541); Bloque 6, km 29 near to Río Tiputini (0.6055°S, 76.4763°W), 236 m (QCAZA 49744); Pompeya (0.4494°S, 76.6113°W), 244 m (QCAZA 33376); 53 km S of the road to Coca, Comunidad Shuar (0.8108°S, 76.8909°W), 292 m (QCAZA 3249); Río Napo, Sacha Lodge, 250 m (QCAZA 5042); Yuturi (0.5572°S, 76.0078°W), 230 m (QCAZA 10381); PROVINCE MORONA SANTIAGO: Napimias (2.8177°S, 77.9987°W), 543 m (QCAZA 46395–97); BRAZIL: STATE AMAZONAS: Sacado (6.7500°S, 70.8500°W), 193 m (INPA 2976); Tabatinga (4.25°S, 69.95°W), 63 m (MZUSP 960042–53); PERU: REGION LORETO: Alto Amazonas, Munichis, Yurimaguas (5.8980°S, 76.2319°W), 149 m (CORBIDI 1744, 1751–54); Maynas, Redondococha (0.5713°S, 75.2192°W), 192 m (CORBIDI 0069–79, 0091, 0097–98); Requena, Comunidad Nativa Galicia (5.4396°S, 73.8515°W), 138 m (CORBIDI 12252–55).

*Dendropsophus triangulum*. BRAZIL: Brazil (BMNH 68.11.15.2; holotype); ACRE: Igarapé Porangaba (8.6666°S, 72.7833°W), 258 m (INPA 4273); Lago Catalao, Ilha Xiborena (MJH 3844); Nova vida (8.3666°S, 72.9833°W), 236 m (INPA 4483); STATE AMAZONAS: Tabatinga (4.25°S, 69.95°W), 63 m (MZUSP 960009, 9600018–20, 960023, 960034, 960056); PARÁ: Obidios (1.9166°S, 55.5166°W), 34 m (MZUSP 950172, 950175–76, 950178); PERU: REGION LORETO: Requena, Comunidad Nativa Galicia (5.4396°S, 73.8515°W), 138 m (CORBIDI 12193–94, 12251); REGION CUZCO: La Convención, Saniri, Malvinas (11.6852°S, 72.9568°W), 387 m (CORBIDI 11204); ECUADOR: PROVINCE SUCUMBÍOS: Garzacocha, Laguna de Mandicocha (0.4981°S, 76.3738°W), 233 m (QCAZA 35504); Santa Cecilia (0.0805°S, 76.9961°W), 340 m (QCAZA 219); PROVINCE ORELLANA: Río Napo, San Vicente (0.6790°S, 75.6511°W), 196 m (QCAZA 44466–76); Río Napo, Chiroisla (0.5756°S, 75.8998°W), 203 m (QCAZA 44455–58, 44290–93); Río Napo, Huiririma (0.7116°S, 75.6239°W), 194 m (QCAZA 44539, 44552); Río Napo, Nuevo Rocafuerte (0.9165°S, 75.4230°W), 194 m (QCAZA 44826–27); Tambococha (0.9736°S, 75.4352°W), 191 m (QCAZA 55249, 55334–35, 55394, 55396).

*Dendropsophus* sp. D. BRAZIL: STATE PARÁ: 200 km west of Redenção (7.65°S, 51.35°W), 275 m (MZUSP 930044); STATE ACRE: Río Branco (9.9666°S, 67.8°W), 138 m (MZUSP 950253–54); PERU: REGION MADRE DE DIOS: 15 km E of Puerto Maldonado (12.5530°S, 69.0638°W), 184 m (KU 215274); Tambopata, La Concesión (12.8512°S, 70.1058°W), 243 m (CORBIDI13405); REGION CUZCO: La Convención, Pagoreni, Malvinas (11.6852°S, 72.9568°W), 478 m (CORBIDI 10045).

*Dendropsophus* sp. E. PERU: REGION CUZCO: La Convención, Pongo de Mainique, Santuar (12.2579°S, 72.8420°W), 643 m (CORBIDI 6631, 6639); La Convención, KP22, Shokoriairi (11.9979°S, 72.9873°W), 592 m (CORBIDI 9738); La Convención, Saniri, Malvinas (11.6852°S, 72.9568°W), 387 m (CORBIDI 11203); La Convención, Plantas Malvinas (11,8384°S, 72,9428°W), 386 m (CORBIDI 10402); REGION MADRE DE DIOS: Tambopata, La Concesión (12.8512°S, 70.1058°W), 243 m (CORBIDI13403).

*Dendropsophus* sp. F. BRAZIL: STATE PARÁ: Alter do Chão (2.5333°S, 54.9666°W), 73 m (MZUSP 950162–63).

*Dendropsophus* sp. G. BRAZIL: STATE PARÁ: 200 km west of Redenção (7.65°S, 51.35°W), 275 m (MZUSP 930042–43, 930045–46, 930049); Serra do Kukoinhokren (7.8333°S, 51.9166°W), 307 m (MTR 6315).

*Dendropsophus bifurcus*. ECUADOR: PROVINCE MORONA SANTIAGO: Río Yunganza (2.8927°S, 78.3852°W), 807 (QCAZA 17021); 4 Km N of Macas, on the road to Puyo (2.888°S, 78.0968°W), 1045 m (QCAZA 23802); 4.8 km N Rosario (2.8858°S, 78.3880°W), 841 m (QCAZA 26428); PROVINCE PASTAZA: Ponoma, Reserva de Bosque Tropical Fundación Hola Vida (1.625°S, 77.9072°W), 831m (QCAZA 37198).

*Dendropsophus brevifrons.* ECUADOR: PROVINCE ORELLANA: Estación Científica Yasuní Km 8 (0.6781°S, 76.3965°W), 240 m (QCAZA 17826); PROVINCE NAPO: Estación Biológica Jatun Sacha (1.0645°S, 77.6128°W), 393 m (QCAZA 18174); PROVINCE SUCUMBÍOS: Playas de Cuyabeno (0.2654°S, 75.8917°W), 230 m (QCAZA 28273).

*Dendropsophus carnifex.* ECUADOR: PROVINCE PICHINCHA: on the road Toachi-Chiriboga (0.3056°N, 78.8819°W), 1315 m (QCAZA 15342); PROVINCE IMBABURA: Santa Rosa, Reserva Biológica Alto Chocó (0.3693°N, 78.4494°W), 2104 m (QCAZA 31770); Santa Rosa, Reserva Siempre Verde (0.3716°N, 78.4218°W), 2468 m (QCAZA 39333).

*Dendropsophus ebraccatus.* ECUADOR: PROVINCE ESMERALDAS: 3 km from Durango (1.0643°N, 78.6443°W), 158 m (QCAZA 15519); Playón de San Francisco (0.8747°N, 78.4529°W), 503 m (QCAZA 40847).

*Dendropsophus parviceps.* ECUADOR: PROVINCE TUNGURAHUA: Río Verde (1.4026°S, 78.2979°W), 1486 m (QCAZA 24425); PROVINCE PASTAZA: Bobonaza, road to Tuculí (1.4945°S, 77.8696°W), 620 m (QCAZA 32555); PROVINCE ORELLANA: Parque Nacional Yasuní PUCE (0.6771°S, 76.4011°W), 251 m (QCAZA 35720); Bloque 15, Yanakinka (0.3373°S, 76.7385°W), 268 m (QCAZA 37692).

*Dendropsophus rhodopeplus.* ECUADOR: PROVINCE ORELLANA: Chiroisla (0.5799°S, 77.9176°W), 207 m (QCAZA 44328–29); Huiririma (0.7116°S, 75.6239°W), 194 m (QCAZA 44584).

*Dendropsophus sarayacuensis.* ECUADOR: PROVINCE NAPO: Río Salado (0.1992°S, 77.6978°W), 1381 m (QCAZA 36697); PROVINCE PASTAZA: Fátima Centro Experimental (1.4113°S, 78 °W), 1023 m (QCAZA 17429); PROVINCE TUNGURAHUA: Baños, Río Lagarto (1.4007°S, 78.2842°W), 1472 m (QCAZA 23030); PROVINCE MORONA SANTIAGO: Nueve de Octubre (1.3928°S, 78.0425°W), 1084 m (QCAZA 32637).
