# Supplementary figures and images for: Systematics of the Dendropsophus leucophyllatus species complex (Anura: Hylidae): Cryptic diversity and the description of two new species
Source: PLoS One. 2017 Mar 1;12(3):e0171785. doi: 10.1371/journal.pone.0171785 (PMC5332023; doi:10.1371/journal.pone.0171785)

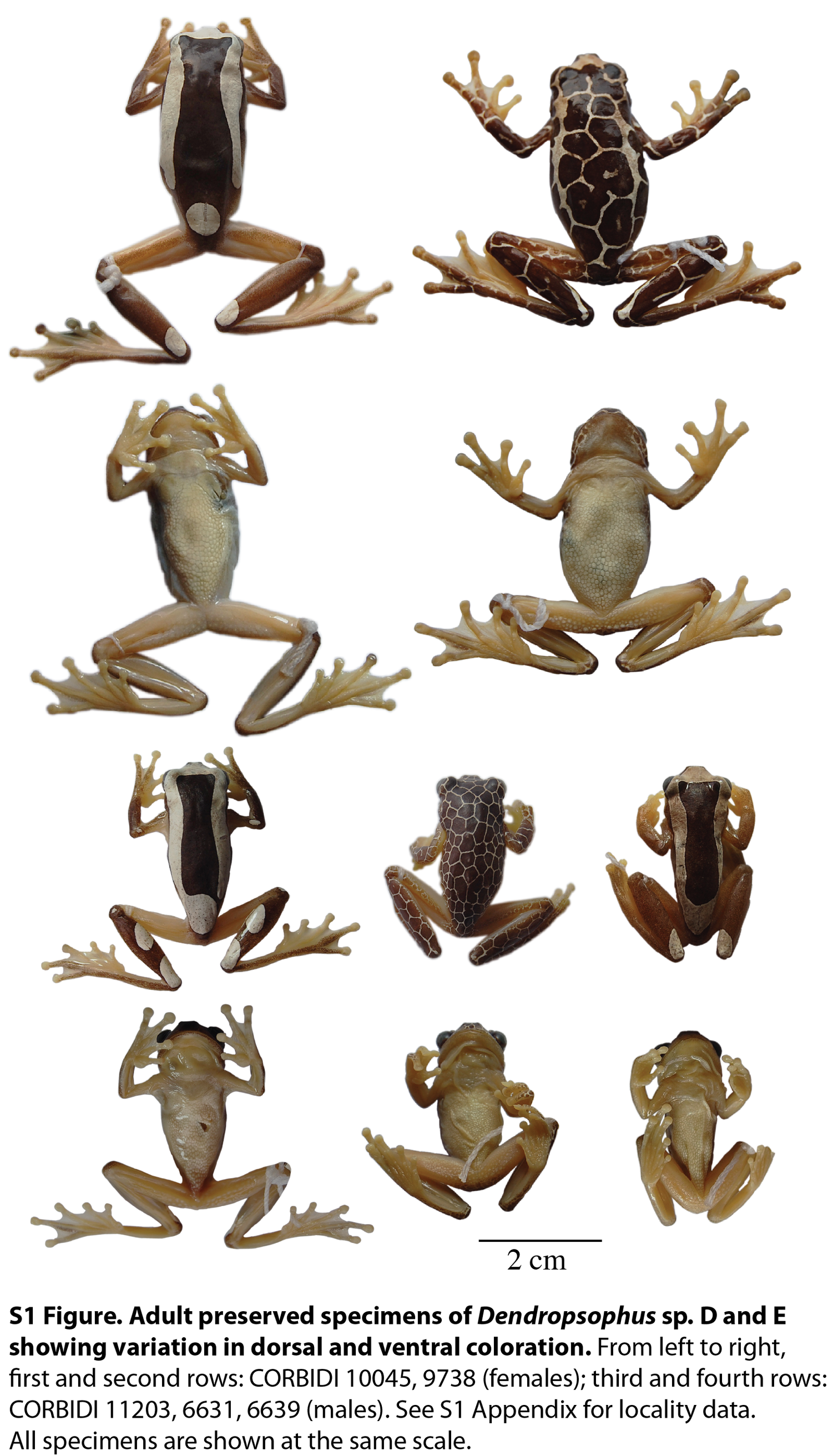

Supplement: S1 Fig — (TIF) [file pone.0171785.s002.tif]
