# Supplementary material for: Systematics of the Dendropsophus leucophyllatus species complex (Anura: Hylidae): Cryptic diversity and the description of two new species
Source: PLoS One. 2017 Mar 1;12(3):e0171785. doi: 10.1371/journal.pone.0171785 (PMC5332023; doi:10.1371/journal.pone.0171785)
Supplement: S1 Table — (DOCX) [file pone.0171785.s003.docx]

**S1 Table.** **Accession names of the sound archives used in the bioacoustics analysis.**

| **Museum No.** | **Sound archives names** | **Species** | **Sources** |
| --- | --- | --- | --- |
| -- | 44 PuertoAlmacen_SantaCruz_BO | *Dendropsophus arndti* | Fonoteca Zoológica of MNCN |
| -- | 45 Beni_BO | *D. arndti* | Fonoteca Zoológica of MNCN |
| SMF88384 | AS0399_NuflodeChavez_BO | *D. arndti* | Jansen et al. 2011 |
| SMF88388 | MJ-98_SanSebantian1_BO | *D. arndti* | Jansen et al. 2011 |
| SMF88389 | MJ-1246_NuflodeChavez_BO | *D. arndti* | Jansen et al. 2011 |
| SMF88390 | MJ-1360_Yucuma_BO | *D. arndti* | Jansen et al. 2011 |
| SMF88393 | MJ-1657_Ichilo_BO | *D. arndti* | Jansen et al. 2011 |
| -- | SanSebastian | *D. arndti* | Jansen et al. 2011 |
| MZUSP950152 | 950152 S,Nav_Mar27-1-AC | *D. leucophyllatus* | Lougheed et al. 2006 |
| MZUSP950153 | 950153 S,Nav_Mar29-5-AC | *D. leucophyllatus* | Lougheed et al. 2006 |
| MZUSP950156 | 950156 S,Nav_Mar29-4-ac | *D. leucophyllatus* | Lougheed et al. 2006 |
| MZUSP950157 | 950157 S,Nav_Mar29-2-AC | *D. leucophyllatus* | Lougheed et al. 2006 |
| MZUSP950158 | 950158 S,Nan_Mar29-1-AC | *D. leucophyllatus* | Lougheed et al. 2006 |
| MZUSP950161 | 950161 A,Chao_Apr4-1-AC | *D. leucophyllatus* | Lougheed et al. 2006 |
| -- | Kaw mountain | *D. leucophyllatus* | AmphibiaWebEcuador website |
| -- | Kaw2 | *D. leucophyllatus* | AmphibiaWebEcuador website |
| -- | Toponowini | *D. leucophyllatus* | AmphibiaWebEcuador website |
| KU143179 | 7695_KU143179_Sucumbios_SantaCecilia_EC | *D. reticulatus* | Fonoteca Zoológica of MNCN |
| KU126484 | 7823_KU126484_Sucumbios_SantaCecilia_EC | *D. reticulatus* | Fonoteca Zoológica of MNCN |
| KU126485 | 7824_KU126485_Sucumbios_SantaCecilia_EC | *D. reticulatus* | Fonoteca Zoológica of MNCN |
| KU143145 | 8063_KU143145_Sucumbios_SantaCecilia_EC | *D. reticulatus* | Fonoteca Zoológica of MNCN |
| -- | LS100450_Loracachi_EC | *D. reticulatus* | AmphibiaWebEcuador website |
| QCAZA267-9 | QCAZ367-9_Misahualli_EC | *D. reticulatus* | AmphibiaWebEcuador website |
| QCAZ49174 | QCAZ49174_Indillama_ViaPompeya_EC | *D. reticulatus* | AmphibiaWebEcuador website |
| -- | 39040_44k_Madre_de_Dios_PE | *D.* sp. D | The Cornell Lab of Ornithology |
| -- | 39053_44k_Madre_de_Dios_PE | *D.* sp. D | The Cornell Lab of Ornithology |
| -- | 39054_44k_Madre_de_Dios_PE | *D.* sp. D | The Cornell Lab of Ornithology |
| -- | 39055_44k_Madre_de_Dios_PE | *D.* sp. D | The Cornell Lab of Ornithology |
| MZUSP950253 | 950253 R,Bran_Apr26-1-DP | *D.* sp. D | Lougheed et al. 2006 |
| MZUSP950254 | 950254 R,Bran_Apr26-2-AC | *D.* sp. D | Lougheed et al. 2006 |
| MZUSP950163 | 950163 A,Chao_Apr4-5-AC | *D.* sp. F | Lougheed et al. 2006 |
| MZUSP950164 | 950164 A,Chao_Apr4-3-AC | *D.* sp. F | Lougheed et al. 2006 |
| MZUSP930042 | 930042_hapl3_AUkre_BR | *D.* sp. G | Lougheed et al. 2006 |
| MZUSP930045 | 930045_hapl3_AUkre_BR | *D.* sp. G | Lougheed et al. 2006 |
| MZUSP930046 | 930046_hapl3_AUkre_BR | *D.* sp. G | Lougheed et al. 2006 |
| MZUSP930047 | 930047_hapl3_AUkre_BR | *D.* sp. G | Lougheed et al. 2006 |
| MZUSP930050 | 930050_hapl3_AUkre_BR | *D.* sp. G | Lougheed et al. 2006 |
| KU143152 | 7702_KU143152_Sucumbios_SantaCecilia_EC | *D. triangulum* | Fonoteca Zoológica of MNCN |
| KU143153 | 7703_KU143153_Sucumbios_SantaCecilia_EC | *D. triangulum* | Fonoteca Zoológica of MNCN |
| KU143154 | 7704_KU143154_Sucumbios_SantaCecilia_EC | *D. triangulum* | Fonoteca Zoológica of MNCN |
| -- | 7832_Sucumbios_SantaCecilia_EC | *D. triangulum* | Fonoteca Zoológica of MNCN |
| KU126421 | 7833_KU126421_Sucumbios_SantaCecilia_EC | *D. triangulum* | Fonoteca Zoológica of MNCN |
| -- | 7834_Sucumbios_SantaCecilia_EC | *D. triangulum* | Fonoteca Zoológica of MNCN |
| -- | 8009_Sucumbios_SantaCecilia_EC | *D. triangulum* | Fonoteca Zoológica of MNCN |
| MZUSP950187 | 950187 Obd_Apr7-4-AC | *D. triangulum* | Lougheed et al. 2006 |
| MZUSP950192 | 950192 Obd_Apr7-1-AC | *D. triangulum* | Lougheed et al. 2006 |
| QCAZA44290 | Chiroisla_QCAZ44290_EC | *D. triangulum* | AmphibiaWebEcuador website |
| -- | Tambococha | *D. triangulum* | AmphibiaWebEcuador website |
