# Supplementary material for: Systematics of the Dendropsophus leucophyllatus species complex (Anura: Hylidae): Cryptic diversity and the description of two new species
Source: PLoS One. 2017 Mar 1;12(3):e0171785. doi: 10.1371/journal.pone.0171785 (PMC5332023; doi:10.1371/journal.pone.0171785)
Supplement: S2 Table — (DOCX) [file pone.0171785.s004.docx]

**S2 Table. Primers used for DNA amplification.**

| **Gen** | **Primer** | **Primer sequence** | **Authors** |
| --- | --- | --- | --- |
| 12S  12S  12S  12S  12S  12S | t-Phe-frog  t-Val-frog  tRNA-val  tPhe2-frog  MVZ59  MVZ50 | ATAGCRCTGAARAYGCTRAGATG  TGTAAGCGARAGGCTTTKGTTAAGCT  GGTGTAAGCGARAGGCTTTKGTTAAG  GCRCTGAARATGCTGAGATGARCCC  ATAGCACTGAAAAYGCTDAGATG  TCTCGGTGTAAGCGAGAGGCTT | Wiens et al. (2005)  Wiens et al. (2005)  Goebel et al. (1999)  Goebel et al. (1999)  Goebel et al. (1999)  Goebel et al. (1999) |
| 16S  16S  16S | 16Sbr-H  16SC-16L  16H47 | CCGGTCTGAACTCAGA TCACGT  GTRGGCCTAAAAGCAGCCAC  AAAGRGCTTAGRTCTTTYGCA | Palumbi et al. (1991)  Pauly et al. (2004)  Heinicke et al. (2007) |
| ND1  ND1  ND1  ND1 | WL384  WL379  16S-frog  tMet-frog | GAGATWGTTTGWGCAACTGCTCG  GCAATAATYATYTGAACMCC  TTACCCTRGGGATAACAGCGCAA  TTGGGGTATGGGCCCAAAAGCT | Moen and Wiens (2008)  Moen and Wiens (2008)  Wiens et al. (2005)  Wiens et al. (2005) |
| CO1  CO1 | LCO1490  dgHCO2198 | GGTCAACAAATCATAAAGATATTGG  TAAACTTCAGGGTGACCAAAR | Folmer et al. (1994)  Folmer et al. (1994) |
| POMC  POMC  POMC  POMC | POMC1  POMC2  POMC3  POMC4 | GAATGTATYAAAGMMTGCAAGATGGWCCT  TAYTGRCCCTTYTTGTGGGCRTT  TCTGCMGARTCWCCYGTGTTTCC  TGGCA TTYTTGAAAAGAGTCA T | Wiens et al. (2005)  Wiens et al. (2005)  Wiens et al. (2005)  Wiens et al. (2005) |
| BDNF  BDNF | BDNFAmp-F1  BDNFAmp-R1 | ACCATCCTTTTCCTKACTATGG  CTATCTTCCCCTTTTAATGGTC | Santos and Cannatella (2011)  Santos and Cannatella (2011) |
| RAG1  RAG1 | R1-GFF  R1-GFR | GAGAAGTCTACAAAAAVGGCAAAG  GAAGCGCCTGAACAGTTTATTAC | Faivovich et al. (2005)  Faivovich et al. (2005) |

**Reference**

Faivovich J, Haddad CFB, García PCA, Frost DR, Campbell, JA, Wheeler WC. Systematic review of the frog family Hylidae, with special reference to Hylinae: phylogenetic analysis and taxonomic revision. Bulletin of the American Museum of Natural History. 2005; 294: 1–240.

Folmer O, Black M, Hoeh W, Lutz R, Vrijenhoek R. DNA primers for amplification of mitochondrial cytochrome c oxidase subunit I from diverse metazoan invertebrates. Molecular Marine Biology and Biotechnology. 1994; 3: 294–299.

Goebel AM, Donnelly JM, Atz ME. PCR primers and amplification methods for 12S ribosomal DNA, the control region, cytochrome oxidase I, and cytochrome b in bufonids and other frogs, and an overview of PCR primers which have amplified DNA in amphibians successfully. Molecular Phylogenetics and Evolution. 1999; 11: 163–199.

Heinicke MP, Duellman WE, Hedges SB. Major Caribbean and Central American frog faunas originated by oceanic dispersal. Proceedings of the National Academy of Sciences of the United States of America. 2007; 104: 10092–10097.

Moen DS, Wiens JJ. Phylogenetic evidence for competitively driven divergence: body-size evolution in Caribbean treefrogs (Hylidae: *Osteopilus*). Evolution. 2008; 63:195–214

Palumbi SR, Martin A, Romano S, McMillan WO, Stice L, Grabowski G. The simple fool’s guide to PCR, version 2.0. Privately published document compiled by S. Palumbi, Department of Zoology, University of Hawaii, Honolulu. 1991.

Pauly GB, Hillis DM, Cannatella DC. The History of a Nearctic Colonization: Molecular Phylogenetics and Biogeography of the Nearctic Toads (*Bufo*). Evolution. 2004; 58: 2517–2535.

Santos JC, Cannatella DC. Phenotypic integration emerges from aposematism and scale in poison frogs. Proceedings of the National Academy of Sciences of the United States of America. 2011; 108: 6175–6180.

Wiens JJ, Fetzner JW, Parkinson CL, Reeder TW. Hylid frog phylogeny and sampling strategies for speciose clades. Systematic Biology. 2005; 54: 719–748.
