# Supplementary material for: Systematics of the Dendropsophus leucophyllatus species complex (Anura: Hylidae): Cryptic diversity and the description of two new species
Source: PLoS One. 2017 Mar 1;12(3):e0171785. doi: 10.1371/journal.pone.0171785 (PMC5332023; doi:10.1371/journal.pone.0171785)
Supplement: S3 Table — (DOCX) [file pone.0171785.s005.docx]

**S3 Table. Genbank accession numbers for DNA sequences used in the phylogenetic analysis.**

| **Museum No.** | **Species** | **Genbank Accession No.** | | | | | | | **Reference** |
| --- | --- | --- | --- | --- | --- | --- | --- | --- | --- |
|  |  | ***12S*** | ***16S*** | ***ND1*** | ***COI*** | ***RAG1*** | ***POMC*** | ***BDNF*** |  |
| SMF88385 | *Dendropsophus arndti* | -- | KY406350 | KY406468 | KY406584 | -- | -- | -- | This study |
| SMF88391 | *D. arndti* | KY406348 | KY406351 | KY406469 | -- | -- | -- | -- | This study |
| SMF88383 | *D. arndti* | KY406269 | KY406352 | KY406470 | KY406585 | KY406712 | KY406692 | KY406723 | This study |
| SMF88384 | *D. arndti* | KY406346 | KY406353 | KY406467 | KY406586 | -- | -- | -- | This study |
| SMF88389 | *D. arndti* | KY406270 | KY406354 | KY406471 | KY406587 | KY406711 | KY406697 | KY406724 | This study |
| SMF88390 | *D. arndti* | KY406347 | KY406355 | KY406472 | KY406588 | -- | KY406696 | -- | This study |
| MNHN2015.127 | *D. leucophyllatus* | KY406255 | KY406356 | -- | -- | -- | -- | -- | This study |
| MNHN2015.128 | *D. leucophyllatus* | KY406256 | KY406357 | -- | -- | -- | -- | -- | This study |
| AF1341 | *D. leucophyllatus* | KY406271 | KY406358 | KY406473 | KY406583 | -- | KY406694 | -- | This study |
| AF1396 | *D. leucophyllatus* | KY406272 | KY406359 | KY406474 | KY406589 | -- | -- | -- | This study |
| AF1397 | *D. leucophyllatus* | KY406273 | KY406360 | KY406475 | KY406590 | -- | -- | -- | This study |
| AF1710 | *D. leucophyllatus* | KY406274 | KY406361 | KY406476 | KY406591 | -- | -- | -- | This study |
| AF1713 | *D. leucophyllatus* | KY406237 | KY406349 | KY406477 | KY406592 | -- | -- | -- | This study |
| AF1748 | *D. leucophyllatus* | KY406275 | KY406362 | KY406478 | KY406593 | -- | KY406695 | -- | This study |
| AF1814 | *D. leucophyllatus* | KY406238 | KY406363 | KY406479 | -- | -- | -- | -- | This study |
| AF1815 | *D. leucophyllatus* | KY406239 | KY406364 | KY406480 | -- | -- | KY406693 | -- | This study |
| MC123 | *D. leucophyllatus* | KY406245 | KY406367 | KY406483 | -- | -- | -- | -- | This study |
| MC124 | *D. leucophyllatus* | KY406284 | KY406368 | KY406484 | KY406596 | -- | -- | -- | This study |
| MC125 | *D. leucophyllatus* | KY406246 | KY406369 | KY406485 | KY406597 | -- | -- | -- | This study |
| MC191 | *D. leucophyllatus* | KY406247 | KY406370 | KY406486 | KY406598 | -- | -- | -- | This study |
| MC192 | *D. leucophyllatus* | KY406248 | KY406371 | KY406487 | KY406599 | -- | -- | -- | This study |
| MC224 | *D. leucophyllatus* | KY406249 | KY406372 | KY406488 | KY406600 | -- | -- | -- | This study |
| MC297 | *D. leucophyllatus* | KY406285 | KY406373 | KY406489 | KY406601 | -- | -- | -- | This study |
| MC300 | *D. leucophyllatus* | KY406250 | KY406374 | KY406490 | KY406602 | -- | -- | -- | This study |
| MC36 | *D. leucophyllatus* | KY406251 | KY406366 | KY406482 | KY406595 | -- | -- | -- | This study |
| MC373 | *D. leucophyllatus* | KY406252 | KY406376 | KY406492 | KY406604 | -- | -- | -- | This study |
| MC404 | *D. leucophyllatus* | KY406253 | KY406375 | KY406491 | KY406603 | -- | -- | -- | This study |
| MC99 | *D. leucophyllatus* | KY406254 | KY406365 | KY406481 | KY406594 | -- | -- | -- | This study |
| MZUSP950143 | *D. leucophyllatus* | -- | AF308087/DQ393417 | -- | -- | -- | -- | -- | Chek et al. 2001; Lougheed et al. 2006 |
| MZUSP950156 | *D. leucophyllatus* | AF308068 | AF308088 | -- | -- | -- | -- | -- | Chek et al. 2001 |
| MZUSP950161 | *D. leucophyllatus* | -- | DQ393416 | -- | -- | -- | -- | -- | Lougheed et al. 2006 |
| MZUSP950193 | *D. leucophyllatus* | -- | DQ393428 | -- | -- | -- | -- | -- | Lougheed et al. 2006 |
| MZUSP950231 | *D. leucophyllatus* | -- | AF308091 | -- | -- | -- | -- | -- | Chek et al. 2001 |
| MZUSP950232 | *D. leucophyllatus* | -- | AF308092/DQ393427 | -- | -- | -- | -- | -- | Chek et al. 2001; Lougheed et al. 2006 |
| BPN918 | *D. leucophyllatus* | JN690789 | JN691396 | -- | -- | -- | -- | -- | Fouquet et al. 2012 |
| INPA2976 | *D. reticulatus* | AF308077 | AF308106/DQ393438 | -- | -- | -- | -- | -- | Chek et al. 2001; Lougheed et al. 2006 |
| MZUSP960042 | *D. reticulatus* | AF308078 | AF308107/DQ393437 | -- | -- | -- | -- | -- | Chek et al. 2001; Lougheed et al. 2006 |
| MZUSP960053 | *D. reticulatus* | -- | AF308108/DQ393439 | -- | -- | -- | -- | -- | Chek et al. 2001; Lougheed et al. 2006 |
| KU202745 | *D. reticulatus* | AY326053 | AY326053 | -- | -- | -- | -- | -- | Darst and Cannatella 2004 |
| KU217664 | *D. reticulatus* | DQ380377 | -- | -- | -- | -- | -- | -- | Wiens et al. 2006 |
| QCAZA12537 | *D. reticulatus* | KY406286 | KY406377 | KY406493 | KY406605 | -- | -- | -- | This study |
| QCAZA17411 | *D. reticulatus* | KY406290 | KY406378 | KY406494 | KY406606 | -- | -- | -- | This study |
| QCAZA28312 | *D. reticulatus* | KY406295 | KY406379 | KY406495 | KY406607 | -- | -- | -- | This study |
| QCAZA37872 | *D. reticulatus* | KY406300 | KY406380 | KY406496 | KY406608 | -- | -- | -- | This study |
| QCAZA37887 | *D. reticulatus* | KY406301 | KY406381 | KY406497 | KY406609 | -- | -- | -- | This study |
| QCAZA39480 | *D. reticulatus* | KY406302 | KY406382 | KY406498 | KY406610 | -- | -- | -- | This study |
| QCAZA43085 | *D. reticulatus* | KY406304 | KY406383 | KY406499 | KY406611 | KY406714 | KY406698 | KY406725 | This study |
| QCAZA43086 | *D. reticulatus* | KY406305 | KY406384 | KY406500 | KY406612 | -- | -- | -- | This study |
| QCAZA43665 | *D. reticulatus* | KY406264 | KY406385 | KY406501 | KY406613 | -- | -- | -- | This study |
| QCAZA43666 | *D. reticulatus* | KY406306 | KY406386 | KY406502 | KY406614 | -- | -- | -- | This study |
| QCAZA43667 | *D. reticulatus* | KY406307 | KY406387 | KY406503 | KY406615 | -- | -- | -- | This study |
| QCAZA43726 | *D. reticulatus* | KY406308 | KY406388 | KY406504 | KY406616 | -- | -- | -- | This study |
| QCAZA43753 | *D. reticulatus* | KY406265 | KY406389 | KY406505 | KY406617 | -- | -- | -- | This study |
| QCAZA43758 | *D. reticulatus* | KY406309 | KY406390 | KY406506 | KY406618 | -- | -- | -- | This study |
| QCAZA43759 | *D. reticulatus* | KY406310 | KY406391 | KY406507 | KY406619 | -- | -- | -- | This study |
| QCAZA43767 | *D. reticulatus* | KY406311 | KY406409 | KY406525 | KY406637 | -- | -- | -- | This study |
| QCAZA44211 | *D. reticulatus* | KY406312 | KY406392 | KY406508 | KY406620 | -- | KY406699 | -- | This study |
| QCAZA44212 | *D. reticulatus* | KY406313 | KY406393 | KY406509 | KY406621 | -- | -- | -- | This study |
| QCAZA44213 | *D. reticulatus* | KY406314 | KY406394 | KY406510 | KY406622 | -- | -- | -- | This study |
| QCAZA44367 | *D. reticulatus* | KY406319 | KY406395 | KY406511 | KY406623 | -- | -- | -- | This study |
| QCAZA44368 | *D. reticulatus* | KY406320 | KY406396 | KY406512 | KY406624 | -- | -- | -- | This study |
| QCAZA44485 | *D. reticulatus* | KY406325 | KY406397 | KY406513 | KY406625 | -- | -- | -- | This study |
| QCAZA44486 | *D. reticulatus* | KY406326 | KY406398 | KY406514 | KY406626 | -- | -- | -- | This study |
| QCAZA44487 | *D. reticulatus* | KY406327 | KY406399 | KY406515 | KY406627 | -- | -- | -- | This study |
| QCAZA44587 | *D. reticulatus* | KY406331 | KY406400 | KY406516 | KY406628 | -- | -- | -- | This study |
| QCAZA44588 | *D. reticulatus* | KY406332 | KY406401 | KY406517 | KY406629 | -- | -- | -- | This study |
| QCAZA44589 | *D. reticulatus* | KY406333 | KY406402 | KY406518 | KY406630 | -- | -- | -- | This study |
| QCAZA44668 | *D. reticulatus* | KY406334 | KY406403 | KY406519 | KY406631 | -- | -- | -- | This study |
| QCAZA44669 | *D. reticulatus* | KY406335 | KY406404 | KY406520 | KY406632 | -- | -- | -- | This study |
| QCAZA44814 | *D. reticulatus* | KY406336 | KY406405 | KY406521 | KY406633 | KY406715 | KY406702 | KY406726 | This study |
| QCAZA44815 | *D. reticulatus* | KY406337 | KY406406 | KY406522 | KY406634 | -- | -- | -- | This study |
| QCAZA44816 | *D. reticulatus* | KY406338 | KY406407 | KY406523 | KY406635 | -- | -- | -- | This study |
| QCAZA46396 | *D. reticulatus* | KY406340 | KY406408 | KY406524 | KY406636 | -- | -- | -- | This study |
| QCAZA46397 | *D. reticulatus* | KY406341 | KY406410 | KY406526 | KY406638 | -- | -- | -- | This study |
| QCAZA48836 | *D. reticulatus* | KY406342 | KY406411 | KY406527 | KY406639 | -- | -- | -- | This study |
| QCAZA48840 | *D. reticulatus* | KY406343 | KY406412 | KY406528 | KY406640 | -- | -- | -- | This study |
| QCAZA49319 | *D. reticulatus* | KY406344 | KY406414 | KY406530 | KY406642 | -- | -- | -- | This study |
| QCAZA49338 | *D. reticulatus* | KY406345 | KY406413 | KY406529 | KY406641 | -- | -- | -- | This study |
| CORBIDI0091 | *D. reticulatus* | -- | KY406415 | KY406531 | KY406643 | -- | -- | -- | This study |
| CORBIDI12252 | *D. reticulatus* | KY406278 | KY406417 | KY406533 | KY406645 | -- | KY406700 | -- | This study |
| CORBIDI12254 | *D. reticulatus* | KY406279 | KY406416 | KY406532 | KY406644 | -- | -- | -- | This study |
| CORBIDI12255 | *D. reticulatus* | KY406280 | KY406418 | KY406534 | KY406646 | -- | KY406701 | -- | This study |
| MZUSP930044 | *D.* sp. D | -- | DQ393421 | -- | -- | -- | -- | -- | Lougheed et al. 2006 |
| MZUSP950253 | *D.* sp. D | AF308072 | AF308097/DQ393432 | -- | -- | -- | -- | -- | Chek et al. 2001; Lougheed et al. 2006 |
| MZUSP950254 | *D.* sp. D | -- | DQ393433 | -- | -- | -- | -- | -- | Lougheed et al. 2006 |
| KU215274 | *D.* sp. D | DQ380360 | -- | -- | -- | -- | -- | -- | Wiens et al. 2006 |
| CORBIDI10045 | *D.* sp. D | KY406240 | KY406419 | KY406535 | KY406647 | KY406713 | KY406703 | KY406727 | This study |
| CORBIDI13405 | *D.* sp. D | KY406282 | -- | -- | KY406648 | -- | -- | -- | This study |
| CORBIDI6631 | *D.* sp. E | KY406241 | KY406424 | KY406540 | KY406650 | KY406716 | KY406704 | KY406728 | This study |
| CORBIDI6639 | *D.* sp. E | KY406242 | KY406425 | KY406541 | KY406651 | KY406717 | KY406705 | KY406729 | This study |
| CORBIDI9738 | *D.* sp. E | KY406244 | KY406423 | KY406539 | KY406649 | -- | -- | -- | This study |
| CORBIDI10402 | *D.* sp. E | KY406276 | KY406421 | KY406537 | -- | -- | -- | -- | This study |
| CORBIDI11203 | *D.* sp. E | KY406234 | KY406422 | KY406538 | -- | -- | -- | -- | This study |
| CORBIDI13403 | *D.* sp. E | KY406281 | KY406420 | KY406536 | -- | -- | -- | -- | This study |
| MZUSP950162 | *D.* sp. F | -- | DQ393422 | -- | -- | -- | -- | -- | Lougheed et al. 2006 |
| MZUSP950163 | *D.* sp. F | AF308069 | AF308089/DQ393419 | -- | -- | -- | -- | -- | Chek et al. 2001; Lougheed et al. 2006 |
| MTR6315 | *D.* sp. G | JN690790 | JN691397 | -- | -- | -- | -- | -- | Fouquet et al. 2012 |
| MZUSP930042 | *D.* sp. G | -- | DQ393418 | -- | -- | -- | -- | -- | Lougheed et al. 2006 |
| MZUSP930043 | *D.* sp. G | -- | DQ393418 | -- | -- | -- | -- | -- | Lougheed et al. 2006 |
| MZUSP930045 | *D.* sp. G | -- | AF308085 | -- | -- | -- | -- | -- | Chek et al. 2001 |
| MZUSP930046 | *D.* sp. G | AF308067 | AF308086 | -- | -- | -- | -- | -- | Chek et al. 2001 |
| MZUSP930049 | *D.* sp. G | -- | DQ393420 | -- | -- | -- | -- | -- | Lougheed et al. 2006 |
| MJH 3844 | *D. triangulum* | AY843680 | AY843680 | -- | -- | -- | -- | -- | Faivovich et al. 2005 |
| INPA4273 | *D. triangulum* | AF308071 | AF308095/DQ393431 | -- | -- | -- | -- | -- | Chek et al. 2001; Lougheed et al. 2006 |
| INPA4483 | *D. triangulum* | -- | AF308096/DQ393430 | -- | -- | -- | -- | -- | Chek et al. 2001; Lougheed et al. 2006 |
| MZUSP950172 | *D. triangulum* | -- | DQ393426 | -- | -- | -- | -- | -- | Lougheed et al. 2006 |
| MZUSP950175 | *D. triangulum* | -- | DQ393423 | -- | -- | -- | -- | -- | Lougheed et al. 2006 |
| MZUSP950176 | *D. triangulum* | -- | AF308090/DQ393424 | -- | -- | -- | -- | -- | Chek et al. 2001; Lougheed et al. 2006 |
| MZUSP950178 | *D. triangulum* | -- | DQ393425 | -- | -- | -- | -- | -- | Lougheed et al. 2006 |
| MZUSP960009 | *D. triangulum* | -- | DQ393435 | -- | -- | -- | -- | -- | Lougheed et al. 2006 |
| MZUSP960018 | *D. triangulum* | AF308070 | AF308093 | -- | -- | -- | -- | -- | Chek et al. 2001 |
| MZUSP960019 | *D. triangulum* | -- | DQ393436 | -- | -- | -- | -- | -- | Lougheed et al. 2006 |
| MZUSP960020 | *D. triangulum* | -- | DQ393436 | -- | -- | -- | -- | -- | Lougheed et al. 2006 |
| MZUSP960023 | *D. triangulum* | -- | DQ393429 | -- | -- | -- | -- | -- | Lougheed et al. 2006 |
| MZUSP960034 | *D. triangulum* | -- | DQ393434 | -- | -- | -- | -- | -- | Lougheed et al. 2006 |
| MZUSP960056 | *D. triangulum* | -- | AF308094 | -- | -- | -- | -- | -- | Chek et al. 2001 |
| QCAZA35504 | *D. triangulum* | KY406296 | KY406426 | KY406542 | KY406652 | -- | -- | -- | This study |
| QCAZA44290 | *D. triangulum* | KY406315 | KY406427 | KY406543 | KY406653 | -- | -- | -- | This study |
| QCAZA44291 | *D. triangulum* | KY406316 | KY406428 | KY406544 | KY406654 | -- | -- | -- | This study |
| QCAZA44292 | *D. triangulum* | KY406317 | KY406429 | KY406545 | KY406655 | -- | -- | -- | This study |
| QCAZA44455 | *D. triangulum* | KY406321 | KY406430 | KY406546 | KY406656 | -- | -- | -- | This study |
| QCAZA44456 | *D. triangulum* | KY406322 | KY406431 | KY406547 | KY406657 | -- | -- | -- | This study |
| QCAZA44457 | *D. triangulum* | KY406323 | KY406432 | KY406548 | KY406658 | KY406718 | KY406707 | KY406730 | This study |
| QCAZA44466 | *D. triangulum* | KY406324 | KY406433 | KY406549 | KY406659 | -- | -- | -- | This study |
| QCAZA44467 | *D. triangulum* | KY406267 | KY406434 | KY406550 | KY406660 | -- | -- | -- | This study |
| QCAZA44468 | *D. triangulum* | KY406268 | KY406435 | KY406551 | KY406661 | -- | -- | -- | This study |
| QCAZA44539 | *D. triangulum* | KY406328 | KY406436 | KY406552 | KY406662 | -- | KY406708 | -- | This study |
| QCAZA44552 | *D. triangulum* | KY406329 | KY406437 | KY406553 | KY406663 | -- | -- | -- | This study |
| QCAZA44826 | *D. triangulum* | KY406339 | KY406438 | KY406554 | KY406664 | KY406719 | KY406706 | KY406731 | This study |
| CORBIDI11204 | *D. triangulum* | KY406232 | KY406440 | KY406556 | -- | -- | -- | -- | This study |
| CORBIDI12194 | *D. triangulum* | KY406233 | KY406441 | KY406557 | KY406666 | -- | -- | -- | This study |
| CORBIDI12251 | *D. triangulum* | KY406277 | KY406439 | KY406555 | KY406665 | -- | -- | -- | This study |
| CORBIDI9613 | *D. vraemi* | KY406283 | KY406452 | KY406568 | KY406677 | KY406720 | KY406709 | KY406732 | This study |
| CORBIDI9614 | *D. vraemi* | KY406243 | KY406453 | KY406569 | KY406678 | KY406721 | KY406710 | KY406722 | This study |
| CFBH 5797 | *D. anceps* | AY843597 | AY843597 | -- | -- | -- | -- | -- | Faivovich et al. 2005 |
| QCAZA17021 | *D. bifurcus* | KY406289 | KY406448 | KY406564 | KY406673 | -- | -- | -- | This study |
| QCAZA23802 | *D. bifurcus* | KY406293 | KY406446 | KY406562 | KY406671 | -- | -- | -- | This study |
| QCAZA26482 | *D. bifurcus* | KY406236 | KY406447 | KY406563 | KY406672 | -- | -- | -- | This study |
| QCAZA37198 | *D. bifurcus* | KY406298 | KY406449 | KY406565 | KY406674 | -- | -- | -- | This study |
| QCAZA15519 | *D. ebraccatus* | KY406288 | KY406450 | KY406566 | KY406675 | -- | -- | -- | This study |
| QCAZA40847 | *D. ebraccatus* | KY406303 | KY406451 | KY406567 | KY406676 | -- | -- | -- | This study |
| LM3135 | *D. elegans* | DQ380355 | -- | -- | -- | -- | -- | -- | Wiens et al. 2006 |
| MZUSP95029 | *D. elegans* | -- | AF308102 | -- | -- | -- | -- | -- | Chek et al. 2001 |
| MZUSP95033 | *D. elegans* | AF308075 | AF308103 | -- | -- | -- | -- | -- | Chek et al. 2001 |
| MHUA-A 7336 | *D. manonegra* | KF009943 | KF009943 | -- | -- | -- | -- | -- | Rivera-Correa and Orrico 2013 |
| MHUA-A 7337 | *D. manonegra* | KF009942 | KF009942 | -- | -- | -- | -- | -- | Rivera-Correa and Orrico 2013 |
| -- | *D. salli* | AY362976 | AY362976 | -- | -- | -- | -- | -- | Jungfer et al. 2010 |
| MNKA 9446 | *D. salli* | -- | JF790043 | -- | -- | -- | -- | -- | Jansen et al. 2011 |
| MNKA 9447 | *D. salli* | -- | JF790044 | -- | -- | -- | -- | -- | Jansen et al. 2011 |
| MNKA 9448 | *D. salli* | -- | JF790045 | -- | -- | -- | -- | -- | Jansen et al. 2011 |
| MNKA 9671 | *D. salli* | -- | JF790039 | -- | -- | -- | -- | -- | Jansen et al. 2011 |
| MNKA 9673 | *D. salli* | -- | JF790040 | -- | -- | -- | -- | -- | Jansen et al. 2011 |
| MNKA 9674 | *D. salli* | -- | JF790041 | -- | -- | -- | -- | -- | Jansen et al. 2011 |
| MNKA 9677 | *D. salli* | -- | JF790042 | -- | -- | -- | -- | -- | Jansen et al. 2011 |
| QCAZA17429 | *D. sarayacuensis* | KY406257 | KY406442 | KY406558 | KY406667 | -- | -- | -- | This study |
| QCAZA23030 | *D. sarayacuensis* | KY406292 | KY406444 | KY406560 | KY406669 | -- | -- | -- | This study |
| QCAZA32637 | *D. sarayacuensis* | KY406261 | KY406443 | KY406559 | KY406668 | -- | -- | -- | This study |
| QCAZA36697 | *D. sarayacuensis* | KY406297 | KY406445 | KY406561 | KY406670 | -- | -- | -- | This study |
| QCAZA18174 | *D. brevifrons* | KY406291 | KY406458 | KY406574 | KY406683 | -- | -- | -- | This study |
| QCAZA17826 | *D. brevifrons* | KY406235 | KY406457 | KY406573 | KY406682 | -- | -- | -- | This study |
| QCAZA28273 | *D. brevifrons* | KY406294 | KY406459 | KY406575 | KY406684 | -- | -- | -- | This study |
| QCAZA31770 | *D. carnifex* | KY406259 | KY406455 | KY406571 | KY406680 | -- | -- | -- | This study |
| QCAZA39333 | *D. carnifex* | KY406263 | KY406456 | KY406572 | KY406681 | -- | -- | -- | This study |
| QCAZA15342 | *D. carnifex* | KY406287 | KY406454 | KY406570 | KY406679 | -- | -- | -- | This study |
| QCAZA37692 | *D. parviceps* | KY406299 | KY406463 | KY406579 | KY406688 | -- | -- | -- | This study |
| QCAZA32555 | *D. parviceps* | KY406260 | KY406460 | KY406576 | KY406685 | -- | -- | -- | This study |
| QCAZA35720 | *D. parviceps* | KY406262 | KY406462 | KY406578 | KY406687 | -- | -- | -- | This study |
| QCAZA24425 | *D. parviceps* | KY406258 | KY406461 | KY406577 | KY406686 | -- | -- | -- | This study |
| QCAZA44328 | *D. rhodopeplus* | KY406266 | KY406464 | KY406580 | KY406689 | -- | -- | -- | This study |
| QCAZA44329 | *D. rhodopeplus* | KY406318 | KY406465 | KY406581 | KY406690 | -- | -- | -- | This study |
| QCAZA44584 | *D. rhodopeplus* | KY406330 | KY406466 | KY406582 | KY406691 | -- | -- | -- | This study |

**Reference**

Chek AA, Lougheed SC, Bogart JP, Boag PT. Perception and history: molecular phylogeny of a diverse group of neotropical frogs, the 30-chromosome *Hyla* (Anura: Hylidae). Molecular Phylogenetics and Evolution. 2001; 18: 370–85.

Darst CR, Cannatella DC. Novel relationships among hyloid frogs inferred from 12S and 16S mitochondrial DNA sequences. Molecular Phylogenetics and Evolution. 2004; 31: 462–475. doi: 10.1016/j.ympev.2003.09.003

Faivovich J, Haddad CFB, García PCA, Frost DR, Campbell, JA, Wheeler WC. Systematic review of the frog family Hylidae, with special reference to Hylinae: phylogenetic analysis and taxonomic revision. Bulletin of the American Museum of Natural History. 2005; 294: 1–240.

Jansen M, Bloch R, Schulze A, Pfenninger M. Integrative inventory of Bolivia’s lowland anurans reveals hidden diversity. Zoologica Scripta. 2011; 40: 567–583. doi: 10.1111/j.1463-6409.2011.00498.x

Jungfer KH, Reichle S, Piskurek O. Description of a new cryptic southwestern Amazonian species of leaf-gluing treefrog, genus *Dendropsophus* (Amphibia: Anura: Hylidae). Salamandra. 2010; 46: 204–213.

Lougheed SC, Austin JD, Bogart JP, Boag PT, Chek AA. Multicharacter perspectives on the evolution of intraspecific differentiation in a Neotropical hylid frog. BMC Evolutionary Biology. 2006; 6: 1–16.

Rivera-Correa M, Orrico VGD. Description and phylogenetic relationships of a new species of treefrog of the *Dendropsophus leucophyllatus* group (Anura: Hylidae) from the Amazon basin of Colombia and with an exceptional color pattern. Zootaxa. 2013; 3686: 447–460.

Wiens JJ, Graham CH, Moen DS, Smith SA, Reeder TW. Evolutionary and ecological causes of the latitudinal diversity gradient in hylid frogs: treefrog trees unearth the roots of high tropical diversity. American Naturalist. 2006; 168: 579–596. doi: 10.1086/507882
